# Comparative efficacy and safety of 20 intravenous pharmaceutical intervention for prevention of etomidate-induced myoclonus: a systematic review and Bayesian network meta-analysis

**DOI:** 10.3389/fphar.2024.1507616

**Published:** 2025-01-23

**Authors:** Lu Chen, Pengxiang Zhou, Zhengqian Li, Ziyang Wu, Suodi Zhai

**Affiliations:** ^1^ Department of Pharmacy, Peking University Third Hospital, Beijing, China; ^2^ Department of Pharmacy, Yantai Yuhuangding Hospital, Yantai, Shandong, China; ^3^ Institute for Drug Evaluation, Peking University Health Science Center, Beijing, China; ^4^ Department of Anesthesiology, Peking University Third Hospital, Beijing, China; ^5^ Department of Pharmacy, The First Affiliated Hospital of Soochow University, Suzhou, China

**Keywords:** Bayesian analysis, etomidate, myoclonus, randomized controlled trials, network meta-analysis

## Abstract

**Objective:**

To compare the efficacy and safety of pharmaceutical interventions to prevent etomidate-induced myoclonus (EIM), providing the optimal intervention for clinical practice.

**Methods:**

PubMed, Embase, the Cochrane Central Register of Controlled Trials, ClinicalTrials.gov, Chinese National Knowledge Infrastructure, WanFang database, and SinoMed database were searched from the inception to sixth May 2024. We included randomized controlled trials (RCTs) comparing intravenous pharmaceutical interventions to prevent EIM with placebo, no intervention, or another pharmaceutical intervention.

**Results:**

Forty-eight RCTs involving 4,768 participants randomly assigned to 20 intravenous pharmaceutical interventions and normal saline were included. Granisetron (odds ratio [OR]: 0.01, 95% confidence interval [CI]: 0.00 to 0.06; one study, moderate certainty) and oxycodone (OR: 0.01, 95% CI: 0.00 to 0.05; three studies, low certainty) was found to be the most effective intervention in reducing the risk of EIM and ranked highest in terms of surface under the cumulative ranking values (94.4% and 89.7% probability), followed by sufentanil (76.5% probability) and remifentanil (74.8% probability). Further subgroup analysis of EIM at mild, moderate-to-severe levels highlighted granisetron and oxycodone as the favorable interventions for reducing EIM. For safety outcomes, the synthesized results indicated that opioids were associated with a higher risk of adverse events (AEs), while no severe AEs were observed.

**Conclusion:**

Moderate-to-low certainty evidence indicated that granisetron and oxycodone may represent the optimal intervention for reducing the risk of overall and moderate-to-severe EIM with a reasonable safety profile, providing the potential interventions for clinical practice.

**Systematic Review Registration:**

https://www.crd.york.ac.uk/PROSPERO/display_record.php?RecordID=291275.

## 1 Introduction

Etomidate is a commonly used intravenous sedative. As a γ-aminobutyric acid (GABA) type A receptor agonist, it is widely used for the rapid induction of anesthesia owing to its rapid onset and favorable hemodynamic properties (Forman, 2011; Komatsu et al., 2013). More than 60% of patients with emergency airway intubation receive intravenous etomidate as an induction agent (Sivilotti et al., 2003). However, etomidate-induced myoclonus (EIM) remains a major problem in clinical practice, with an incidence rate of 50%–80% after induction (Morel et al., 2011). Myoclonus can increase oxygen consumption and accelerate metabolism, posing a vital threat to patients with conditions such as open globe injuries, emergency surgery without fasting (i.e., risk of regurgitation and aspiration), coronary heart disease, epilepsy, or intracranial aneurysm (Gultop et al., 2010; Zhu et al., 2017). In addition, clinicians may face certain intraoperative challenges and risks when treating patients with EIM.

Several published systematic reviews and pairwise meta-analyses have summarized several options for preventing EIM, including remifentanil (Lang et al., 2019), butorphanol (Hua et al., 2019; Zhu et al., 2019), lidocaine (Lang et al., 2018), dexmedetomidine (Du et al., 2017), and midazolam (Zhou et al., 2017). However, there are no guideline or consensus to standardize this issue, and the order in which these interventions should be prioritized remains unknown. Network meta-analysis (NMA) enables simultaneous comparisons among multiple treatments based on direct and indirect evidence, in turn allowing researchers to rank the relative effectiveness of multiple treatment options (González-Xuriguera et al., 2021). Although one previous NMA investigated preventive intervention strategies for EIM, its results provide limited insight into the true value of each individual intervention because it only evaluated efficacy by drug type such as μ opioid agonists, κ opioid agonists and NMDA-R antagonists, without safety evaluations (Zhang et al., 2022). The result of the such comparison was not convincing, and the efficacy of the specific drug remains unknown, leaving clinicians uncertain of the most suitable pharmaceutical intervention choice. Multiple clinical trials were recently published; hence, updated evidence regarding the optimal intervention for EIM is essential for advancing this area of research.

Therefore, we conducted a systematic review and NMA to comprehensively estimate and rank the comparative efficacy and safety of individual pharmaceutical interventions to prevent EIM and identify the optimal clinical strategy.

## 2 Materials and methods

The NMA was performed in accordance with Preferred Reporting Items for Systematic Review and Meta-Analysis extension statement for NMA (Supplementary Table S1) (Hutton et al., 2015). This study was registered in PROSPERO (CRD42021291275).

### 2.1 Eligibility criteria

Inclusion criteria: studies including patients with American Society of Anesthesiologists (ASA) physical status I to III who underwent etomidate anesthesia before surgery or invasive intervention. All studies were randomized controlled trials (RCTs) comparing intravenous pharmaceutical interventions to prevent EIM with either placebo, no intervention, or another pharmaceutical intervention of interest, which included but were not limited to fentanyl, remifentanil, midazolam, dexmedetomidine, lidocaine, magnesium sulfate, butorphanol, or low-dose etomidate. The primary outcome was the overall risk of EIM. The secondary outcomes included the risk of myoclonus at mild, moderate-to-severe intensity levels, as well as the duration of myoclonus. Mild myoclonus was defined as short contraction of some muscle fibers (e.g., on the finger or shoulder), moderate myoclonus referred to contraction of different groups of muscles (e.g., on the face and leg), and severe myoclonus was determined as an intense clonic movement in two or more muscle groups (e.g., fast adduction of a limb or whole-body movements) (Lang et al., 2018). The secondary outcomes included the risk of drug-related adverse events (AEs). No restrictions on publication status, year, language, or patient age were applied.

Exclusion criteria: we excluded patients who met any one of the following criteria: (1) severe cardiopulmonary or neuropsychological diseases, surgical contraindications, adrenal cortex dysfunction, renal or hepatic diseases, history of seizure disorder, or allergic reaction to etomidate; (2) intolerance to study drugs; (3) sedative, analgesic, or opioid drug use on the day of surgery; (4) pregnancy or lactation; and (5) current use of steroid medication. Conference abstracts, editorials, animal experiments, and studies for which the outcomes of interest were not reported or calculable based on the published reports were also excluded.

### 2.2 Data sources and searches

PubMed, Embase, the Cochrane Central Register of Controlled Trials, ClinicalTrials. gov, China National Knowledge Infrastructure, WanFang database, and SinoMed database were searched from inception to sixth May 2024. The search terms included combinations of “etomidate,” “myoclonus,” “randomized controlled trial,” and their synonyms. In addition, a manual search of the reference lists of relevant studies was performed to identify additional eligible studies. The detailed search strategy is shown in Supplementary Table S2.

### 2.3 Study selection and data extraction

Each trial was independently evaluated by two reviewers (C.L. and Z.P.X.) for screening and data extraction. After checking for duplicate studies, irrelevant studies were eliminated by reviewing the titles and abstracts correspondence with the eligibility criteria. Subsequently, the full texts were screened to confirm eligible studies. A pre-designed form was used to extract trial characteristics, including (1) publication information (publication year, first author, countries), (2) participant information (sample size, age, gender, type of surgery, ASA physical status, and dose of etomidate for anesthesia induction), (3) intervention/comparator (active drugs, dosages), and (4) outcomes. Any discrepancies were resolved through discussion, or by a third reviewer (Z.S.D.).

### 2.4 Quality assessment

Two reviewers (C.L. and Z.P.X.) independently assessed the risk of bias using the Cochrane Collaboration’s Risk of Bias 2 (RoB V.2.0) tool, which includes the following domains: randomization process, deviations from intended interventions, missing outcome data, outcome measurement, and selection of the reported result (Sterne et al., 2019). Each study was classified as low risk, some concerns, or high risk. The same reviewers assessed the quality of evidence regarding the primary outcome using the Grading of Recommendations Assessment, Development and Evaluation (GRADE) framework. The quality of evidence was classified into four levels (high, moderate, low, and very low) according to the following dimensions: risk of bias, indirectness, inconsistency, imprecision, and publication bias (Palmer et al., 2021). Any discrepancies were resolved through discussion or by a third reviewer (Z.S.D.).

### 2.5 Data synthesis and analysis

For dichotomous outcomes, we estimated the results using odds ratio (OR) with 95% confidence intervals (CIs). For continuous outcomes, the mean differences (MD) with 95% CIs were used. The Q test and I^2^ statistic were used to assess heterogeneity among studies. Heterogeneity was considered significant when p < 0.10 and I^2^ > 50% (Higgins et al., 2003). For both direct and indirect comparisons between any pair of comparators (existing closed loops), the node-splitting approach was used to examine the consistency between the direct and indirect evidence. We performed a traditional pairwise meta-analysis and generated network plots for different outcomes among studies to illustrate the geometries using Stata software (version 15.0) and clarify which interventions were compared directly or indirectly in the included studies (Chaimani et al., 2013).

A random-effects NMA was performed within a Bayesian framework using the Markov chain Monte Carlo simulation method in OpenBUGS (version 3.2.3). Model convergence was evaluated via visual inspection of four chains after considering the Brooks-Gelman-Rubin diagnostic as well as trace and density plots (Brooks and Gelman, 1998). Within the Bayesian framework, all interventions were ranked using the surface under the cumulative ranking (SUCRA) curve (Salanti et al., 2011). The SUCRA values are 0 and 1 when interventions are certain to be the worst and best, respectively (Riley et al., 2017).

Subgroup analyses were performed by drug dosages when sufficient information was available. A meta-regression method was used to analyze differences in baseline characteristics (Salanti, 2012). In addition, a sensitivity analysis was performed to examine the robustness of our results. A comparison-adjusted funnel plot and Egger test were used to evaluate the small-study effects for the individual outcome when no less than 10 eligible studies were available (Sterne et al., 2011). Statistical significance was set at p < 0.05.

## 3 Results

### 3.1 Study selection and characteristics

**FIGURE 1:**
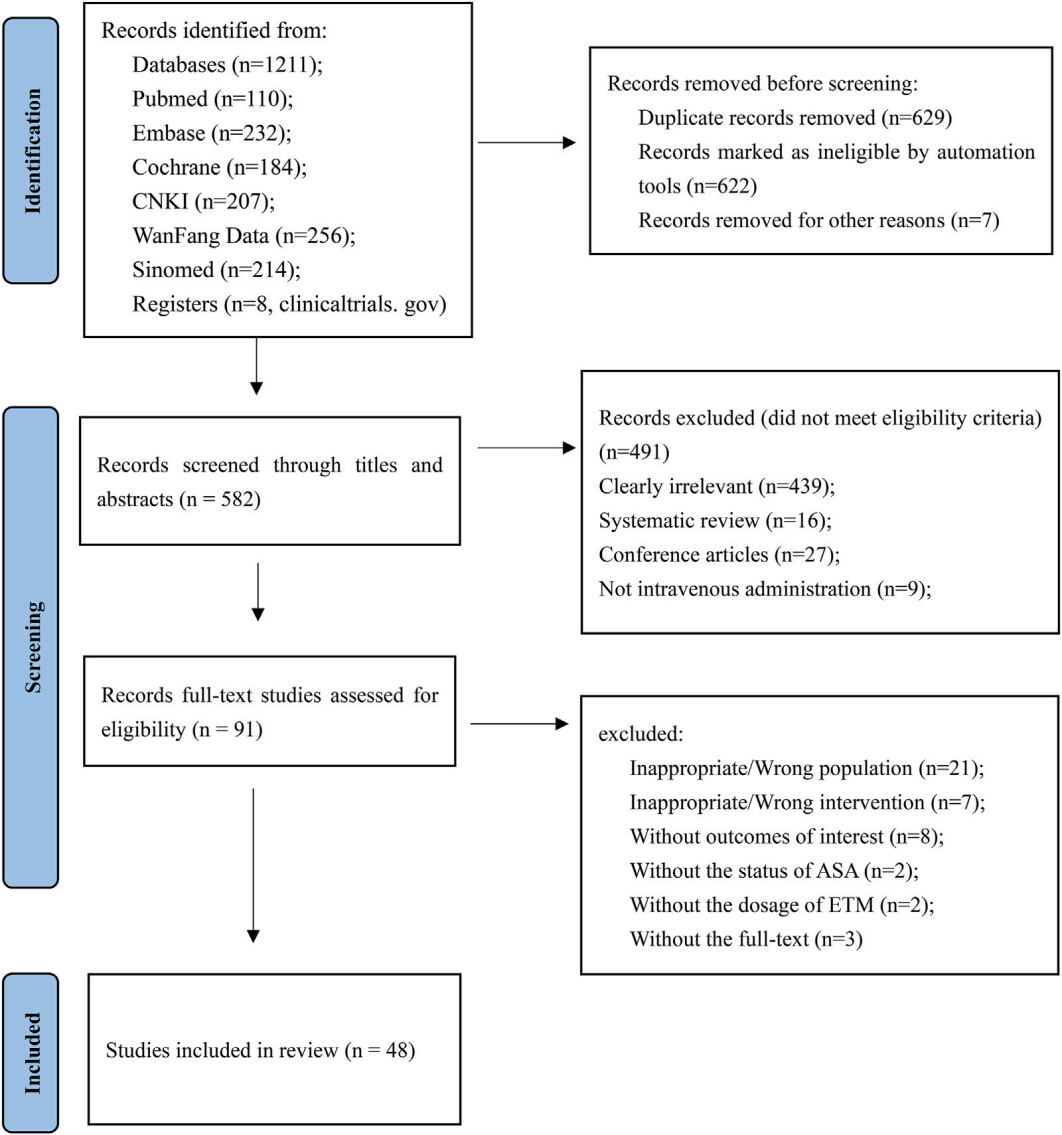
Study selection process.

We identified 1,211 studies, of which 91 potentially eligible studies underwent review of the full text. After applying the eligibility criteria, 48 studies were included in the NMA. The study selection process is illustrated in Figure 1.

The NMA was performed based on 48 RCTs with a total of 4,768 patients enrolled to receive 20 interventions, including alfentanil, fentanyl, sufentanil, remifentanil, oxycodone, midazolam, dexmedetomidine, lignocaine, nalbuphine, butorphanol, magnesium sulfate, tramadol, rocuronium, vecuronium, thiopental, ketamine, low-dose etomidate, low-dose propofol, granisetron, nalmefene, and normal saline (NS) (Kwon MS et al., 2002; Guler et al., 2005; Aissaoui et al., 2006; Cho SY et al., 2008; Choi et al., 2008; Hwang et al., 2008; Chen et al., 2009; Lee et al., 2009; Yu et al., 2009; Gultop et al., 2010; Mizrak et al., 2010; Un et al., 2011; Ko et al., 2013; Ren et al., 2013; He et al., 2014; Singh et al., 2014; Luan et al., 2015; Ma et al., 2015; Malay et al., 2015; Wang et al., 2015; Zhang et al., 2015a; Zhang et al., 2015b; Alipour et al., 2016; Chen et al., 2016; Sedighinejad et al., 2016; Wu et al., 2016; Xie et al., 2016; An et al., 2017; Chen et al., 2017; Fu et al., 2018; Gupta and Gupta, 2018a; Gupta and Gupta, 2018b; Lv et al., 2018; Mullick et al., 2018; Wang and Wang, 2018; Wang et al., 2018b; Miao et al., 2019; Ren et al., 2019; Zhang et al., 2021; Yan et al., 2022; Agrawal et al., 2023; Alipour et al., 2023; RAJKUMAR et al., 2023; Rani et al., 2023; Rautela et al., 2023; Shan et al., 2023; Siddiqui et al., 2023a; Siddiqui et al., 2023b). The baseline characteristics of the included studies are summarized in Supplementary Table S3. Two studies were four-arm trials, 13 studies were three-arm trials, and the remaining were double-arm trials. In total, 41 (85.4%) studies were NS-controlled, 20 (41.7%) studies included more than one active drug, 23 (47.9%) studies were conducted in China, and only 11 (22.9%) studies mentioned the specific types of elective surgery. The mean age was 46.2 years (standard deviation: 10.1), and the median observation time for myoclonus was 2 min after etomidate administration. The median sample size in the individual studies was 90 participants (range: 30 - 284).

The results of the risk of bias assessment are summarized in Supplementary Supplementary Figure S1 and Supplementary Table S4. Overall, 23 (47.9%) studies had low risk of bias and 13 (27.1%) studies were evaluated as having some concerns, primarily owing to a lack of allocation concealment. In this NMA, the majority of primary outcomes were subjective indicators, and 12 (25.0%%) studies were assessed as high risk due to insufficient information regarding the blinding of assessors to outcomes. All studies were assessed as having a low risk of bias for missing outcome data.

### 3.2 Network meta-analysis

**FIGURE 2:**
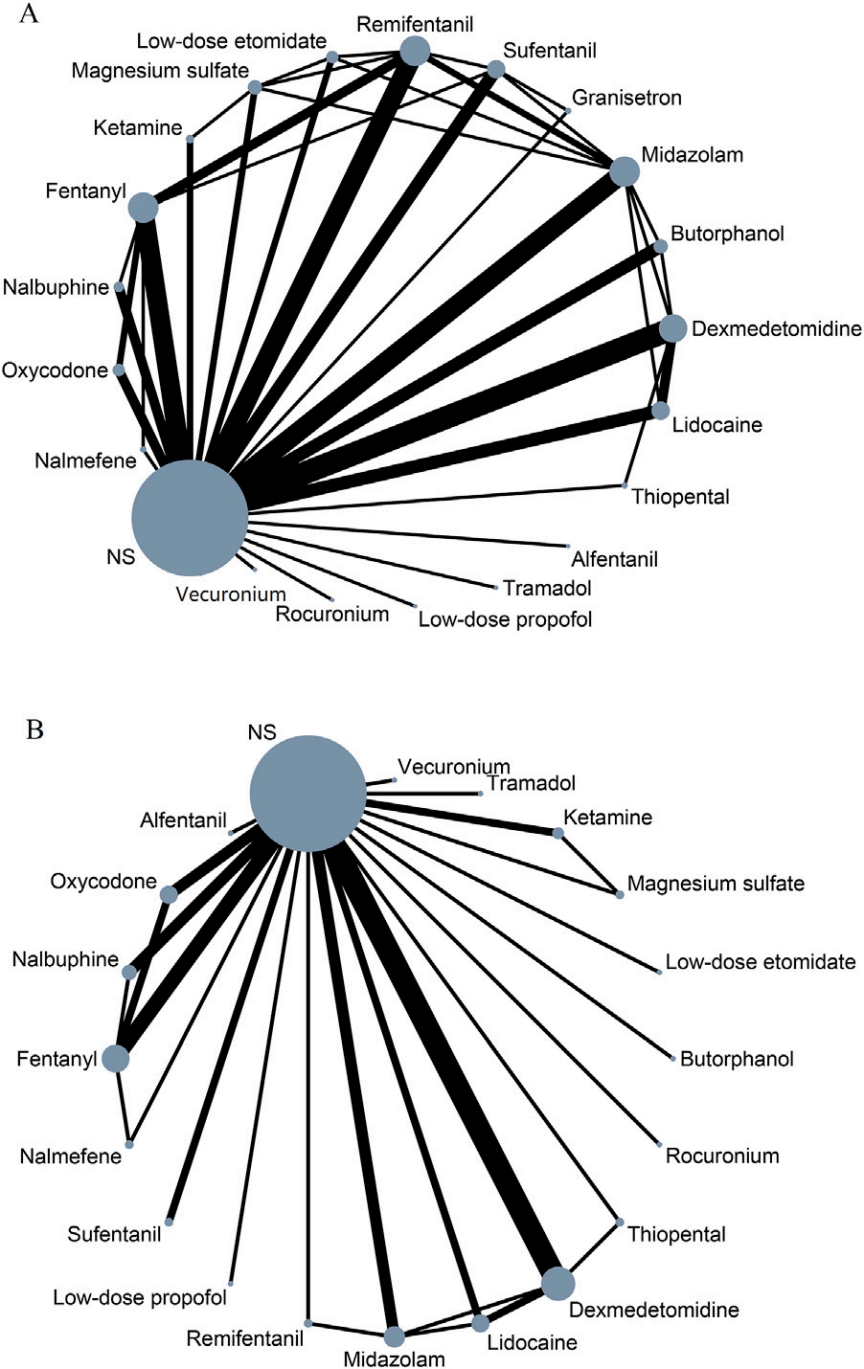
Network meta-analysis of eligible comparisons for primary outcome **(A)** and safety **(B)**. NS, normal saline.

In NMA, each unique node represents an intervention. The size of the node corresponds to the number of patients for each intervention. Lines indicate direct head-to-head comparisons, and the line width corresponds to the number of trials in the comparison (Cipriani et al., 2018; Kim et al., 2020; Burry et al., 2021). Figure 2 shows the network of eligible comparisons for primary outcome and safety.

#### 3.2.1 Overall risk of etomidate-induced myoclonus

**FIGURE 3:**
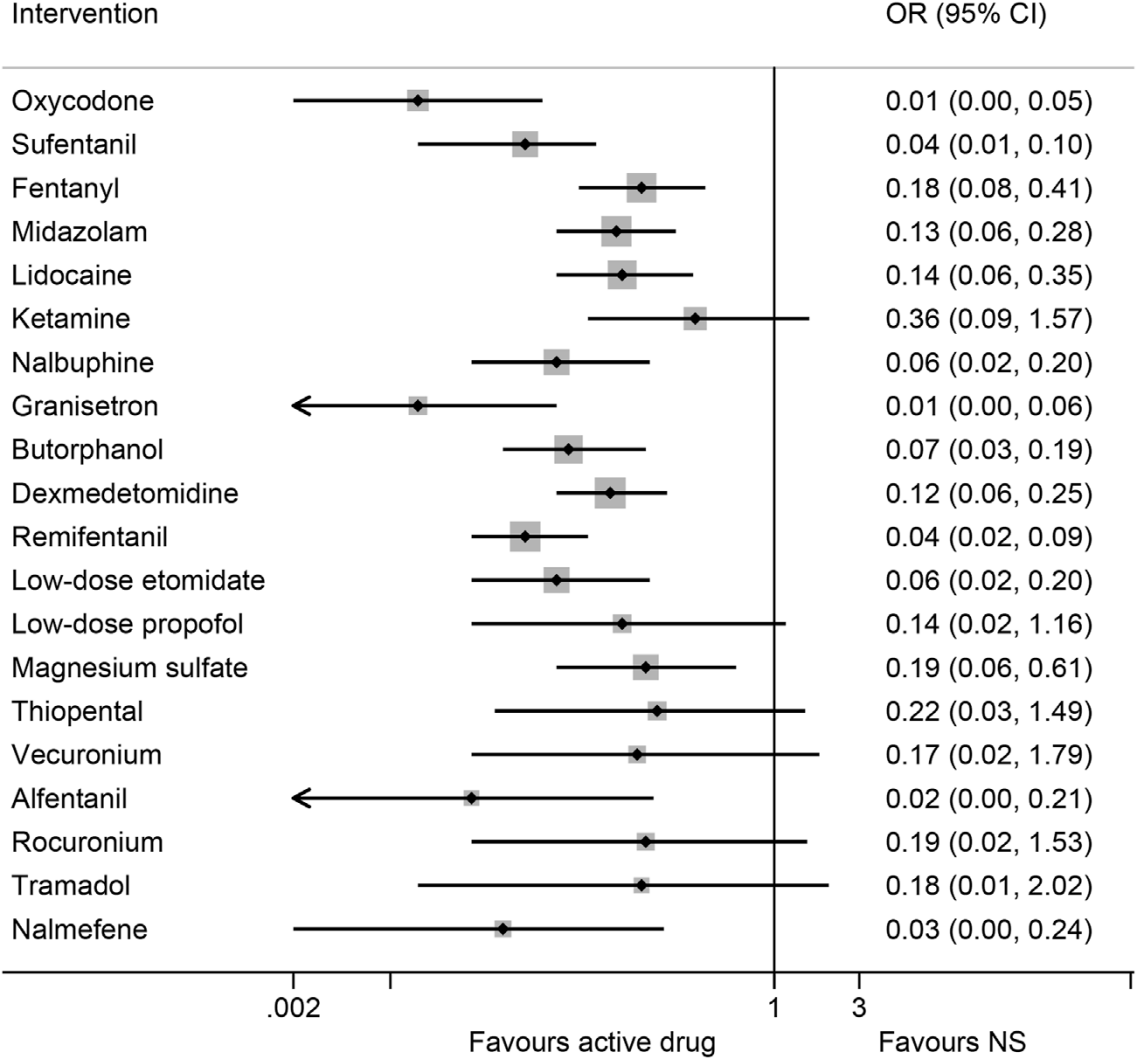
Forest plot of network meta-analysis of all trials for primary outcome. OR: odds ratio, OR < 1 indicated a lower risk of etomidate-induced myoclonus than normal saline (NS). Cl, confidence interval.

Data were analyzed from 48 studies on the risk of EIM, consisting of 4,768 patients with 20 interventions (Kwon MS et al., 2002; Guler et al., 2005; Aissaoui et al., 2006; Cho SY et al., 2008; Choi et al., 2008; Hwang et al., 2008; Chen et al., 2009; Lee et al., 2009; Yu et al., 2009; Gultop et al., 2010; Mizrak et al., 2010; Un et al., 2011; Ko et al., 2013; Ren et al., 2013; He et al., 2014; Singh et al., 2014; Luan et al., 2015; Ma et al., 2015; Malay et al., 2015; Wang et al., 2015; Zhang et al., 2015a; Zhang et al., 2015b; Alipour et al., 2016; Chen et al., 2016; Sedighinejad et al., 2016; Wu et al., 2016; Xie et al., 2016; An et al., 2017; Chen et al., 2017; Fu et al., 2018; Gupta and Gupta, 2018a; Gupta and Gupta, 2018b; Lv et al., 2018; Mullick et al., 2018; Wang and Wang, 2018; Wang et al., 2018b; Miao et al., 2019; Ren et al., 2019; Zhang et al., 2021; Yan et al., 2022; Agrawal et al., 2023; Alipour et al., 2023; RAJKUMAR et al., 2023; Rani et al., 2023; Rautela et al., 2023; Shan et al., 2023; Siddiqui et al., 2023a; Siddiqui et al., 2023b). According to the synthesized results of the traditional pairwise comparison (Supplementary Table S5A), all interventions, except for ketamine (OR 0.28, 95% CI 0.04–2.23) and tramadol (OR: 0.21, 95% CI: 0.05–1.01), were associated with lower myoclonus rates than NS. In the network analysis, 14 drugs (oxycodone, remifentanil, sufentanil, fentanyl, alfentanil, midazolam, lidocaine, nalbuphine, butorphanol, dexmedetomidine, low-dose etomidate, granisetron, nalmefene, and magnesium sulfate) significantly reduced the overall risk of myoclonus compared with NS, except for ketamine, tramadol, thiopental, rocuronium, low-dose propofol, and vecuronium (Figure 3; Table 1). Granisetron (OR: 0.01, 95% CI: 0.00–0.06) and oxycodone (OR: 0.01, 95% CI: 0.00–0.05) was associated with the most significant reduction in myoclonus, followed by alfentanil (OR: 0.02, 95% CI 0.00–0.21), and sufentanil (OR: 0.04, 95% CI: 0.01–0.10). In addition, the ranking of treatments based on SUCRA values (Supplementary Figure S3A; Supplementary Table S8) with a probability of more than 75% was as follows: granisetron (94.4%), oxycodone (89.7%%), alfentanil (82.8%%), and sufentanil (76.5%). The node-splitting method revealed no inconsistency between direct and indirect evidence (Supplementary Table S7A). The comparison-adjusted funnel plot for the overall risk of myoclonus indicated possible asymmetry, whereas Egger’s test (p = 0.07) revealed no significant difference among the studies, suggesting a low risk of publication bias (Supplementary Figure S4A).

**TABLE 1:** Network league table for primary outcome (upper-right portion) and safety (lower-left portion).

NS	0.01 (0.00, 0.05)	0.18 (0.08, 0.41)	0.13 (0.06, 0.28)	0.04 (0.02, 0.09)	0.04 (0.01, 0.10)	0.12 (0.06, 0.25)	0.22 (0.03, 1.49)	0.14 (0.06, 0.35)	0.07 (0.03, 0.19)	0.36 (0.09, 1.57)	0.19 (0.06, 0.61)	0.06 (0.02, 0.20)	0.06 (0.02, 0.20)	0.19 (0.02, 1.53)	0.02 (0.00, 0.21)	0.18 (0.01, 2.02)	0.17 (0.02, 1.79)	0.01 (0.00, 0.06)	0.03 (0.00, 0.24)	0.14 (0.02, 1.16)
0.08 (0.00, 0.85)	oxycodone	12.18 (2.94, 65.59)	9.22 (2.06, 52.71)	2.69 (0.54, 15.74)	2.42 (0.43, 16.27)	8.25 (1.84, 46.66)	14.78 (1.45, 187.20)	9.57 (1.92, 57.91)	5.06 (0.99, 32.21)	25.18 (3.69, 212.06)	13.11 (2.27, 96.59)	4.11 (0.70, 30.12)	4.42 (0.80, 30.12)	13.16 (1.10, 182.22)	1.21 (0.06, 22.06)	12.20 (0.60, 232.45)	11.85 (0.77, 202.98)	0.41 (0.02, 7.20)	2.09 (0.19, 27.02)	9.28 (0.76, 139.98)
0.28 (0.02, 1.74)	3.44 (0.33, 47.97)	fentanyl	0.74 (0.25, 2.32)	0.22 (0.07, 0.68)	0.20 (0.05, 0.73)	0.67 (0.22, 2.05)	1.20 (0.14, 9.79)	0.78 (0.22, 2.72)	0.41 (0.11, 1.47)	2.04 (0.40, 10.75)	1.06 (0.26, 4.39)	0.33 (0.08, 1.38)	0.36 (0.10, 1.33)	1.05 (0.11, 10.11)	0.10 (0.01, 1.34)	0.99 (0.05, 13.57)	0.96 (0.08, 11.43)	0.03 (0.00, 0.41)	0.17 (0.02, 1.34)	0.75 (0.08, 7.59)
1.91 (0.20, 16.78)	_	6.75 (0.43, 211.75)	midazolam	0.29 (0.10, 0.83)	0.26 (0.08, 0.88)	0.89 (0.33, 2.39)	1.60 (0.20, 13.07)	1.04 (0.34, 3.14)	0.55 (0.17, 1.77)	2.71 (0.55, 14.07)	1.42 (0.38, 5.20)	0.44 (0.12, 1.63)	0.48 (0.12, 1.85)	1.42 (0.15, 12.91)	0.13 (0.01, 1.78)	1.31 (0.07, 17.05)	1.26 (0.10, 15.18)	0.04 (0.00, 0.53)	0.23 (0.02, 1.96)	1.01 (0.10, 9.98)
0.00 (0.00, 0.05)	0.00 (0.00, 0.92)	0.00 (0.00, 0.24)	0.00 (0.00, 0.03)	remifentanil	0.90 (0.23, 3.69)	3.05 (0.99, 10.07)	5.57 (0.63, 46.82)	3.55 (1.03, 13.07)	1.88 (0.51, 7.23)	9.30 (1.79, 53.11)	4.88 (1.26, 19.32)	1.52 (0.40, 6.27)	1.64 (0.38, 7.24)	4.85 (0.50, 48.03)	0.44 (0.03, 6.46)	4.51 (0.25, 64.35)	4.37 (0.34, 55.29)	0.15 (0.01, 1.97)	0.78 (0.08, 7.33)	3.45 (0.35, 37.12)
0.49 (0.02, 6.26)	5.86 (0.14, 374.33)	1.72 (0.05, 60.84)	0.25 (0.00, 7.19)	—	sufentanil	3.40 (0.93, 12.62)	6.16 (0.64, 57.32)	3.97 (0.98, 16.55)	2.09 (0.50, 9.02)	10.45 (1.72, 63.71)	5.42 (1.11, 26.06)	1.69 (0.34, 8.41)	1.83 (0.38, 8.81)	5.40 (0.49, 56.77)	0.50 (0.03, 7.56)	5.03 (0.25, 74.43)	4.79 (0.33, 65.10)	0.17 (0.01, 1.78)	0.87 (0.08, 8.79)	3.84 (0.36, 42.49)
1.03 (0.20, 3.86)	12.33 (0.79, 435.97)	3.61 (0.35, 60.82)	0.54 (0.05, 4.91)	—	2.11 (0.10, 65.57)	dexmedetomidine	1.79 (0.25, 12.72)	1.17 (0.44, 3.09)	0.61 (0.20, 1.88)	3.04 (0.62, 15.50)	1.59 (0.40, 6.31)	0.50 (0.12, 1.98)	0.53 (0.13, 2.14)	1.58 (0.17, 14.82)	0.15 (0.01, 1.98)	1.47 (0.08, 19.15)	1.42 (0.12, 16.57)	0.05 (0.00, 0.60)	0.25 (0.03, 2.29)	1.13 (0.12, 11.27)
1.65 (0.07, 31.77)	—	5.81 (0.19, 334.67)	0.86 (0.02, 32.01)	—	3.40 (0.07, 301.19)	1.61 (0.08, 36.52)	thiopental	0.65 (0.08, 5.52)	0.34 (0.04, 2.98)	1.70 (0.15, 19.56)	0.88 (0.09, 8.44)	0.28 (0.03, 2.77)	0.30 (0.03, 2.98)	0.89 (0.05, 15.75)	0.08 (0.00, 1.94)	0.82 (0.03, 19.46)	0.79 (0.04, 16.61)	0.03 (0.00, 0.61)	0.14 (0.01, 2.40)	0.63 (0.03, 11.78)
2.00 (0.24, 14.18)	—	7.11 (0.49, 185.32)	1.05 (0.08, 12.59)	—	4.14 (0.15, 185.78)	1.96 (0.27, 15.93)	1.22 (0.04, 39.86)	lidocaine	0.53 (0.15, 1.90)	2.62 (0.48, 14.69)	1.37 (0.31, 5.98)	0.43 (0.10, 1.89)	0.46 (0.10, 2.04)	1.37 (0.14, 13.34)	0.13 (0.01, 1.81)	1.27 (0.07, 17.72)	1.22 (0.09, 15.62)	0.04 (0.00, 0.55)	0.22 (0.02, 2.12)	0.98 (0.09, 10.12)
0.00 (0.00, 0.06)	0.00 (0.00, 1.11)	0.00 (0.00, 0.28)	0.00 (0.00, 0.04)	—	0.00 (0.00, 0.17)	0.00 (0.00, 0.07)	0.00 (0.00, 0.05)	0.00 (0.00, 0.03)	butorphanol	4.94 (0.89, 28.88)	2.59 (0.58, 11.40)	0.81 (0.18, 3.73)	0.87 (0.20, 3.93)	2.62 (0.25, 26.19)	0.24 (0.01, 3.47)	2.40 (0.13, 33.36)	2.32 (0.17, 30.10)	0.08 (0.00, 1.05)	0.41 (0.04, 4.00)	1.85 (0.18, 19.32)
0.51 (0.04, 6.68)	6.25 (0.22, 495.85)	1.81 (0.09, 78.44)	0.27 (0.01, 8.36)	—	1.05 (0.03, 73.96)	0.50 (0.03, 11.38)	0.31 (0.01, 17.85)	0.25 (0.01, 7.46)	—	ketamine	0.53 (0.10, 2.68)	0.16 (0.03, 1.00)	0.18 (0.03, 1.10)	0.52 (0.04, 6.50)	0.05 (0.00, 0.87)	0.49 (0.02, 8.35)	0.46 (0.03, 7.38)	0.02 (0.00, 0.26)	0.08 (0.01, 1.01)	0.37 (0.03, 4.95)
2.11 (0.08, 56.87)	—	7.47 (0.21, 580.80)	1.10 (0.02, 60.98)	—	4.36 (0.08, 504.74)	2.05 (0.07, 88.15)	1.28 (0.02, 120.98)	1.05 (0.02, 55.21)	—	4.15 (0.16, 109.77)	magnesium sulfate	0.31 (0.07, 1.45)	0.34 (0.06, 1.71)	1.00 (0.09, 10.81)	0.09 (0.00, 1.44)	0.92 (0.04, 14.25)	0.88 (0.06, 12.20)	0.03 (0.00, 0.45)	0.16 (0.01, 1.64)	0.71 (0.06, 8.24)
3.15 (0.10, 101.38)	—	—	1.64 (0.03, 107.08)	—	6.50 (0.10, 876.12)	3.08 (0.08, 157.22)	1.90 (0.02, 209.48)	1.56 (0.03, 97.29)	—	6.18 (0.08, 463.42)	1.50 (0.01, 176.92)	low-dose etomidate	1.08 (0.21, 5.55)	3.22 (0.29, 34.75)	0.29 (0.02, 4.82)	2.96 (0.15, 44.84)	2.84 (0.19, 40.32)	0.10 (0.01, 1.45)	0.51 (0.04, 5.43)	2.28 (0.19, 27.30)
0.72 (0.10, 4.15)	8.81 (0.58, 312.26)	2.55 (0.31, 37.31)	0.38 (0.02, 6.42)	—	1.48 (0.06, 60.25)	0.70 (0.07, 7.91)	0.44 (0.01, 14.87)	0.36 (0.02, 5.57)	—	1.42 (0.05, 30.69)	0.34 (0.01, 13.46)	0.23 (0.00, 10.49)	nalbuphine	2.98 (0.26, 32.44)	0.27 (0.01, 4.23)	2.78 (0.14, 41.77)	2.64 (0.18, 37.64)	0.09 (0.01, 1.33)	0.48 (0.04, 4.70)	2.10 (0.19, 24.41)
2.41 (0.07, 81.08)	—	8.58 (0.20, 794.10)	1.27 (0.02, 84.21)	—	5.02 (0.08, 682.03)	2.36 (0.06, 125.39)	1.46 (0.02, 165.74)	1.20 (0.02, 77.35)	—	4.76 (0.06, 367.78)	1.15 (0.01, 139.01)	0.77 (0.01, 105.93)	3.35 (0.07, 196.51)	rocuronium	0.09 (0.00, 2.37)	0.91 (0.03, 23.05)	0.88 (0.04, 22.07)	0.03 (0.00, 0.80)	0.16 (0.01, 2.97)	0.71 (0.04, 15.52)
0.00 (0.00, 0.08)	0.00 (0.00, 1.48)	0.00 (0.00, 0.36)	0.00 (0.00, 0.05)	—	0.00 (0.00, 0.24)	0.00 (0.00, 0.09)	0.00 (0.00, 0.07)	0.00 (0.00, 0.05)	—	0.00 (0.00, 0.20)	0.00 (0.00, 0.05)	0.00 (0.00, 0.04)	0.00 (0.00, 0.13)	0.00 (0.00, 0.05)	alfentanil	10.29 (0.25, 380.06)	9.80 (0.29, 340.85)	0.34 (0.01, 12.76)	1.74 (0.07, 50.64)	7.92 (0.28, 246.22)
0.00 (0.00, 0.02)	0.00 (0.00, 0.34)	0.00 (0.00, 0.09)	0.00 (0.00, 0.01)	—	0.00 (0.00, 0.06)	0.00 (0.00, 0.02)	0.00 (0.00, 0.02)	0.00 (0.00, 0.01)	—	0.00 (0.00, 0.05)	0.00 (0.00, 0.01)	0.00 (0.00, 0.01)	0.00 (0.00, 0.03)	0.00 (0.00, 0.01)	—	tramadol	0.95 (0.03, 33.25)	0.03 (0.00, 1.37)	0.17 (0.01, 5.13)	0.78 (0.03, 24.80)
1.72 (0.04, 84.42)		6.23 (0.11, 757.64)	0.91 (0.01, 81.75)	—	3.63 (0.04, 637.98)	1.70 (0.03, 123.84)	1.05 (0.01, 156.23)	0.86 (0.01, 75.83)	—	3.42 (0.03, 360.53)	0.82 (0.01, 131.96)	0.55 (0.00, 98.92)	2.41 (0.04, 191.30)	0.71 (0.00, 133.21)	—	—	vecuronium	0.04 (0.00, 1.02)	0.18 (0.01, 4.27)	0.81 (0.03, 20.95)
																		granisetron	5.09 (0.21, 139.20)	22.79 (0.92, 680.36)
4.63 (0.16, 88.85)	—	16.33 (0.86, 467.58)	2.41 (0.04, 97.10)	—	9.51 (0.16, 780.82)	4.50 (0.14, 138.18)	2.80 (0.03, 195.62)	2.29 (0.05, 87.49)	—	9.08 (0.13, 441.83)	2.19 (0.02, 172.18)	1.47 (0.01, 129.70)	6.40 (0.18, 188.03)	1.92 (0.01, 169.25)	—	—	2.65 (0.02, 309.67)	—	nalmefene	4.44 (0.24, 93.06)
1.02 (0.01, 129.20)	—	3.73 (0.02, 972.95)	0.54 (0.00, 111.99)	—	2.17 (0.01, 780.57)	1.01 (0.01, 175.52)	0.62 (0.00, 196.15)	0.51 (0.00, 104.06)	—	2.00 (0.01, 478.51)	0.48 (0.00, 164.42)	0.32 (0.00, 121.09)	1.42 (0.01, 269.01)	0.42 (0.00, 159.20)	—	—	0.58 (0.00, 275.79)	—	0.22 (0.00, 80.11)	low-dose propofol

Effect sizes represent summary odds ratio and 95% confidence intervals. For the upper triangle (the overall risk of EIM), and the lower triangle (the risk of AEs), OR<1 favor the column intervention, which values are in bold. NS, normal saline; EIM, etomidate-induced myoclonus; AEs, adverse events. “—” Indicate the effect size with very width credible interval owing to the small sample sizes, which were not shown in this table.

#### 3.2.2 Different intensity levels of myoclonus

A total of 47 studies reported the risk of myoclonus at mild and moderate-to-severe levels among 4,668 patients who had received 20 interventions (Supplementary Figure S2A) (Kwon MS et al., 2002; Guler et al., 2005; Aissaoui et al., 2006; Cho SY et al., 2008; Choi et al., 2008; Hwang et al., 2008; Chen et al., 2009; Lee et al., 2009; Yu et al., 2009; Gultop et al., 2010; Mizrak et al., 2010; Ko et al., 2013; Ren et al., 2013; He et al., 2014; Singh et al., 2014; Luan et al., 2015; Ma et al., 2015; Malay et al., 2015; Wang et al., 2015; Zhang et al., 2015a; Zhang et al., 2015b; Alipour et al., 2016; Chen et al., 2016; Sedighinejad et al., 2016; Wu et al., 2016; Xie et al., 2016; An et al., 2017; Chen et al., 2017; Fu et al., 2018; Gupta M. and Gupta P., 2018; Gupta P. and Gupta M., 2018; Lv et al., 2018; Mullick et al., 2018; Wang and Wang, 2018; Wang W. et al., 2018; Miao et al., 2019; Ren et al., 2019; Zhang et al., 2021; Yan et al., 2022; Agrawal et al., 2023; Alipour et al., 2023; RAJKUMAR et al., 2023; Rani et al., 2023; Rautela et al., 2023; Shan et al., 2023; Siddiqui et al., 2023a; Siddiqui et al., 2023b).

In terms of the risk of myoclonus at a mild level, the traditional pairwise comparison (Supplementary Table S5B) showed that seven drugs were associated with a lower risk of myoclonus than NS, including granisetron (OR: 0.04, 95% CI: 0.01–0.20), oxycodone (OR: 0.17, 95% CI: 0.08–0.38), dexmedetomidine (OR: 0.49, 95% CI: 0.31–0.77), remifentanil (OR: 0.25, 95% CI: 0.13–0.48), fentanyl (OR: 0.44, 95% CI: 0.25–0.75), sufentanil (OR: 0.24, 95% CI: 0.06–0.99), and butorphanol (OR: 0.42, 95% CI: 0.22–0.79). In contrast, tramadol increased the mild myoclonus rates when compared with NS (OR: 6.00, 95% CI: 1.29–27.91). In the NMA, the synthesized results demonstrated that granisetron (OR: 0.05, 95% CI: 0.00–0.42) and oxycodone (OR: 0.10, 95% CI: 0.02–0.37) was the most effective in reducing the risk of mild myoclonus compared with NS, followed by magnesium sulfate (OR: 0.18, 95% CI: 0.04–0.75), nalbuphine (OR: 0.29, 95% CI 0.09–0.91), sufentanil (OR: 0.30, 95% CI 0.10–0.88), remifentanil (OR: 0.29, 95% CI 0.09–0.91), lidocaine (OR: 0.38, 95% CI 0.16–0.2), and butorphanol (OR: 0.38, 95% CI 0.15–0.99), while there were no statistically significant differences between the other interventions (Supplementary Table S6A). Based on SUCRA values (Supplementary Figure S3B; Supplementary Table S8), the top three interventions were granisetron (93.1%), oxycodone (89.2%), and magnesium sulfate (78.6%). The node-splitting analysis indicated that seven of the 28 comparisons were inconsistent (Supplementary Table S7B). The comparison-adjusted funnel plot and Egger test (p = 0.51) showed no asymmetry (Supplementary Figure S4B).

In terms of the risk of myoclonus at moderate-to-severe level, the traditional pairwise comparison (Supplementary Table S5C) revealed that 19 drugs were associated with a lower risk of moderate-to-severe myoclonus than NS, except for thiopental (OR: 0.30, 95% CI 0.09–1.00). In the NMA, 13 drugs were associated with a statistically significant reduction in the risk of moderate-to-severe myoclonus when compared with NS, with granisetron, nalmefene, oxycodone, and remifentanil exerting the most significant effects (Supplementary Table S6B). According to SUCRA values (Supplementary Figure S3C; Supplementary Table S8), the top four interventions were nalmefene (97.6%), granisetron (96.8%), oxycodone (81.5%), and remifentanil (79.7%). In the node-splitting analysis, 4 of the 27 comparisons were inconsistent. The comparison-adjusted funnel plot and Egger’s test (p = 0.12) indicated a low risk of publication bias (Supplementary Table S7C; Supplementary Figure S4C).

#### 3.2.3 Adverse events

AEs were reported in 28 studies including a total of 2,704 patients and 19 interventions (Guler et al., 2005; Aissaoui et al., 2006; Cho SY et al., 2008; Choi et al., 2008; Hwang et al., 2008; Yu et al., 2009; Mizrak et al., 2010; He et al., 2014; Singh et al., 2014; Luan et al., 2015; Malay et al., 2015; Wang et al., 2015; Chen et al., 2016; Wu et al., 2016; An et al., 2017; Chen et al., 2017; Fu et al., 2018; Gupta M. and Gupta P., 2018; Lv et al., 2018; Wang W. et al., 2018; Miao et al., 2019; Ren et al., 2019; Zhang et al., 2021; Yan et al., 2022; Agrawal et al., 2023; Shan et al., 2023; Siddiqui et al., 2023a; Siddiqui et al., 2023b). Commonly reported AEs included nausea, vomiting, dizziness, coughing, headache, injection pain, bradycardia, hypotension, respiratory depression, and myalgia. In the traditional pairwise analysis and NMA (Supplementary Table S5D; Table 1), five drugs exhibited significant associations with an increased risk of AEs when compared with NS, including oxycodone, remifentanil, tramadol, alfentanil, and butorphanol. Nevertheless, these findings showed significant point estimates, although with small sample sizes and the absence of any AEs occurring in the NS groups resulting in wide CIs. Common AEs in patients treated with oxycodone, butorphanol, remifentanil, and fentanyl were dizziness, bradycardia, and hypotension, respectively. Details regarding other AEs are listed in Supplementary Table S10. Additionally, two studies reported a head-to-head comparison of doses among tramadol and alfentanil, which found that respiratory depression and bradycardia occurred only in the 2.0 mg/kg tramadol groups and 10.0 μg/kg alfentanil groups. Six studies comparing dexmedetomidine reported an increased risk of bradycardia in the 1.0 μg/kg groups, whereas there were no significant differences between the other doses.

Further analyses were not performed for the remaining active drugs because of the small number of included studies. Nevertheless, only one study reported that one participant in the remifentanil group had withdrawn owing to chest rigidity (Hwang et al., 2008). Other AEs did not appear to affect the results of each study. In the node-splitting analysis, 1 of the 9 comparisons were inconsistent. (Supplementary Table S7E). A comparison-adjusted funnel plot demonstrated possible asymmetry, while Egger’s linear regression (p = 0.26) indicated no significant difference among studies, which indicated a low risk of publication bias (Supplementary Figure S4D).

#### 3.2.4 Duration of myoclonus

Only five studies reported the duration of EIM, including one three-arm trial for midazolam, lidocaine, and NS. The remaining four studies were double-arm trials (Supplementary Figure S2B) (Kwon MS et al., 2002; Cho SY et al., 2008; Lee et al., 2009; Singh et al., 2014; Alipour et al., 2016). In the traditional pairwise comparison (Supplementary Table S5E), remifentanil (MD: −57.80, 95% CI: −81.18 to −34.42) and alfentanil (MD: −37.20, 95% CI: −69.94 to −4.46) were associated with a shorter duration of myoclonus than NS. However, the synthesized results of the NMA indicated that no intervention significantly reduced the duration of myoclonus when compared with NS or another intervention (Supplementary Table S6C). There was insufficient information to perform analyses of consistency and publication bias.

### 3.3 Heterogeneity, subgroup, and sensitivity analysis

Pairwise comparisons of heterogeneity in primary outcome estimates are presented in Supplementary Table S5. Briefly, significant heterogeneity was detected in the subgroup comparisons of NS with oxycodone, sufentanil, lidocaine, ketamine, and low-dose etomidate. Further subgroup analyses were performed for active drugs at different dosages, which revealed evident reductions in I^2^ values, with most reaching less than 50%. Other sources of heterogeneity may have included differences in the time of observation and the induction dose of etomidate, without sufficient information for further analysis. According to the meta-regression results, similarities in clinical characteristics were observed across all the included studies (Supplementary Table S11). In addition, to assess the robustness of the pooled results, we conducted sensitivity analyses by excluding trials evaluated as having a high risk of bias overall, which yielded no material change in the results or conclusions (Supplementary Table S9; Supplementary Figure S3D, Supplementary Figure S5).

### 3.4 Quality of evidence

Among 32 mixed comparisons (i.e., combining direct and indirect evidence), the confidence in the estimates for primary outcomes was rated as very low in 3 comparisons, low in 16, and moderate in 13. Among the nine direct comparisons, the confidence in the estimate was moderate in five comparisons, low in three, and very low in one. Among the 169 indirect comparisons, the confidence in the estimate was very low in 104 comparisons and low in 60 (Supplementary Table S12). The major reason for downgrading the certainty of evidence was the imprecision of the results with wide CIs and sample sizes, risk of bias, and heterogeneity. Most comparisons yielded low-certainty evidence due to these concerns.

## 4 Discussion

This NMA was based on 48 RCTs included 4,768 patients randomly assigned to 20 pharmaceutical interventions and NS to assess their ability to prevent EIM. To the best of our knowledge, this is the largest comprehensive systematic review to summarize the comparative efficacy and safety of all available pharmaceutical interventions for EIM prevention using NMA approach. As such, our study provides the strongest evidence regarding optimal selection of interventions for anesthetists to manage EIM in clinical practice by synthesizing all available direct and indirect evidence. In addition, the NMA provides evidence-based information that will aid researchers with further clinical investigations.

In this study, we focused on the role of interventions in reducing the overall risk of EIM. According to the synthesized results, oxycodone, remifentanil, sufentanil, fentanyl, alfentanil, midazolam, lidocaine, nalbuphine, butorphanol, dexmedetomidine, low-dose etomidate, granisetron, nalmefene, and magnesium sulfate were associated with lower myoclonus rates compared with NS. In contrast, ketamine, tramadol, thiopental, rocuronium, low-dose propofol, and vecuronium showed effective point estimates, although the results did not reach statistical significance because of the low number of patients included, resulting in wide CIs. Subgroup analyses of myoclonus at mild and moderate-to- severe intensities revealed that granisetron, oxycodone, sufentanil, and remifentanil were effective in reducing the risk of EIM in each of these subgroups. Based on SUCRA rankings for the overall risk of EIM and for EIM at mild, moderate-to-severe intensities, granisetron was ranked first (one study, 92 patients, moderate certainty), followed by oxycodone (three studies, 340 patients, low certainty) and remifentanil (six studies, 359 patients, low certainty). However, due to limited up-to-date studies focusing on these drugs, the conclusions should be interpreted cautiously, considering the sparse data available. Furthermore, the heterogeneity analysis results indicated that the nature of the effect sizes remained unchanged, and there was no inconsistency between direct and indirect evidence and no significant publication bias in terms of efficacy and safety outcomes. Sensitivity analyses further revealed that the pooled results remained stable after excluding studies with a high risk of bias.

Although active drugs that prevent EIM may increase the risk of AEs, no severe AEs were reported for the 20 active drugs, which may have affected our results. However, opioids were associated with more AEs than NS and other drugs, possibly representing a dose-response effect. Commonly observed AEs in patients treated with oxycodone, remifentanil, and fentanyl included dizziness, bradycardia, and hypotension, respectively. One study reported that alfentanil (10 μg/kg) was associated with the highest risk of respiratory depression and bradycardia (Cho SY et al., 2008). Another study reported that tramadol (2.0 mg/kg) had the highest probability of causing respiratory depression and dizziness (Fu et al., 2018). The results were consistent with those of previous meta-analyses (Qiu et al., 2016; Wang J. et al., 2018; Lang et al., 2019). In clinical practice, smaller doses of active drugs with comparable efficacy are preferred when attempting to reduce EIM risk; however, the number of studies was insufficient for analyzing dose–response relationships, highlighting the need for additional well-designed trials with large sample sizes.

At present, EIM exerts effects via GABA receptors, inhibiting the function of the brainstem reticular structures, which in turn leads to disinhibition of subcortical structures and other low-level centers, ultimately causing myoclonus (Doenicke et al., 1999; Hueter et al., 2003). Activation of the ҡ receptor has been reported to produce strong anticonvulsant effects, affecting N-methyl D-aspartate channels, BZD-GABA(A) chloride channel complexes, and GABA receptors. Additionally, µ receptors can activate GABA(A) receptors in the basal ganglia region, thereby reducing myoclonus (Manocha et al., 2003a; Manocha et al., 2003b; Honar et al., 2004; Loacker et al., 2007). Opioids, particularly oxycodone or remifentanil respectively mainly act both on µ and ҡ receptors or µ receptors, which may explain their effectiveness in reducing EIM risk (Staahl et al., 2006). Nevertheless, the functional mechanism of granisetron on reducing EIM remain uncertain. A previous study showed that the risk of propofol-induced myoclonus with granisetron was only 5.5% and most of the patients (94.5%) experienced myoclonic movements with grade zero (without myoclonus) (Alipour et al., 2014). Further well-designed RCTs with larger sample sizes and further explore the mechanism of action, especially in terms of pharmaceutical, cellular, and molecular properties can be required to verify the new and valuable pretreatment.

Notably, this NMA represents a substantial improvement in the context of the current literature as it provides insights more appropriate to clinical practice than the previously published systematic review. While the previous review indicated that low-dose etomidate was the best intervention for preventing EIM only based on two small sample-size trials among nine drug types, and evaluated as providing high-quality evidence. However, the synthesized results of the study were a combination of intravenous and oral administration trials, lacking the further heterogeneity and similarity analysis (Zhang et al., 2022). Our NMA included both efficacy and safety information for 20 individual intravenous drugs to prevent EIM. In contrast, low-dose etomidate was not ranked higher than oxycodone (0.05–1.0 mg/kg) and granisetron (40 μg/kg) based on SUCRA values in this NMA, and the confidence level was rated as low owing to the heterogeneity and high risk of bias among relevant studies, as detailed in Supplementary Table S12.

## 5 Limitation

This study had some limitations to consider. First, 25% of the studies were evaluated as having a high risk of bias owing to part of the outcomes were subjective indicators with insufficient information on the blinding, but the results remained stable by excluding these trials in sensitivity analysis. Second, most studies with small sample sizes resulted in wide CIs, which probably leading to a general downgrade in the GRADE assessment. Third, the optimal recommended dose may not be determined because of the limited number of studies in which comparisons were performed among various interventions.

## 6 Conclusion

In this NMA, moderate-to-low certainty evidence indicated that granisetron and oxycodone may represent the optimal intervention for reducing the risk of overall and moderate-to-severe EIM with a reasonable safety profile. Further well-designed RCTs with larger sample sizes and detailed dosage information are required to verify these findings.

## Data Availability

The original contributions presented in the study are included in the article/Supplementary Material, further inquiries can be directed to the corresponding author.

## References

[B1] Agrawal M. Tambey R. Patil V. (2023). A prospective randomized controlled study to compare effectiveness of dexmedetomidine and lignocaine pre-treatment for prevention of etomidate induced myoclonus. J. Cardiovasc. Dis. Res. 14 (10), 321–331.

[B2] Aissaoui Y. Belyamani L. Wali A. E. Hajjouji S. Kamili N. D. (2006). Prevention of myoclonus after etomidate using a priming dose. Ann. Franaises Danesthèsie De Rèanimation 25 (10), 1041–1045. 10.1016/j.annfar.2006.07.079 17005362

[B3] Alipour M. Abdi N. Zaj P. Mashhadi L. (2023). Efficacy of granisetron versus sufentanil on reducing myoclonic movements following etomidate: double-blind, randomised clinical trial. Sultan Qaboos Univ. Med. J. 23 (3), 380–386. 10.18295/squmj.1.2023.009 37655076 PMC10467562

[B4] Alipour M. Tabari M. Alipour M. (2014). Paracetamol, ondansetron, granisetron, magnesium sulfate and lidocaine and reduced propofol injection pain. Iran. Red. Crescent Med. J. 16 (3), e16086. 10.5812/ircmj.16086 24829787 PMC4005449

[B5] Alipour M. Tabari M. Azad A. M. (2016). Comparative study evaluating efficacy of sufentanil versus midazolam in preventing myoclonic movements following etomidate. J. Anaesthesiol. Clin. Pharmacol. 32 (1), 29–32. 10.4103/0970-9185.173382 27006537 PMC4784209

[B6] An X. Li C. Sahebally Z. Wen X. Zhao B. Fang X. (2017). Pretreatment with oxycodone simultaneously reduces etomidate-induced myoclonus and rocuronium-induced withdrawal movements during rapid-sequence induction. Med. Sci. Monit. 23, 4989–4994. 10.12659/msm.902652 29046518 PMC5659139

[B7] Brooks S. P. Gelman A. (1998). General methods for monitoring convergence of iterative simulations. J. Comput. Graph. Statistics 7 (4), 434. 10.2307/1390675

[B8] Burry L. D. Cheng W. Williamson D. R. Adhikari N. K. Egerod I. Kanji S. (2021). Pharmacological and non-pharmacological interventions to prevent delirium in critically ill patients: a systematic review and network meta-analysis. Intensive Care Med. 47 (9), 943–960. 10.1007/s00134-021-06490-3 34379152 PMC8356549

[B9] Chaimani A. Higgins J. P. Mavridis D. Spyridonos P. Salanti G. (2013). Graphical tools for network meta-analysis in STATA. PLoS One 8 (10), e76654. 10.1371/journal.pone.0076654 24098547 PMC3789683

[B10] Chen L. Huang H. Xiong L. Chen X. (2017). Effect of sufentanil administration on myoclonus induced by etomidate. Guangxi Med. J. 39 (6), 2. 10.11675/j.issn.0253-4304.2017.06.43

[B11] Chen Y. Wang G. Yu Y. (2009). Effects of different opioids on myoclonus and bispectral index during etomidate induction. Tianjin Med. J. 37 (5), 3.

[B12] Chen Z. Cheng C. Li D. Yuan Y. Shen J. (2016). Efficacy of dexmedetomidine in reducing etomidate-induced myoclonus. Jiangsu Med. J. 42 (21), 3. 10.19460/j.cnki.0253-3685.2016.21.011

[B13] Cho S. Y. Jeon W. J. Nam Y. M. Yeom J. H. Kh K. (2008). The optimal dosage of alfentanil pretreatment for prevention of myoclonus after injection of etomidate. Korean J. Anesthesiol. 55 (3), 320–325. 10.4097/kjae.2008.55.3.320

[B14] Choi J. M. Choi I. C. Yong B. J. Kim T. H. Hahm K. D. (2008). Pretreatment of rocuronium reduces the frequency and severity of etomidate-induced myoclonus. J. Clin. Anesth. 20 (8), 601–604. 10.1016/j.jclinane.2008.06.010 19100933

[B15] Cipriani A. Furukawa T. A. Salanti G. Chaimani A. Atkinson L. Z. Ogawa Y. (2018). Comparative efficacy and acceptability of 21 antidepressant drugs for the acute treatment of adults with major depressive disorder: a systematic review and network meta-analysis. Lancet 391 (10128), 1357–1366. 10.1016/s0140-6736(17)32802-7 29477251 PMC5889788

[B16] Doenicke A. W. Roizen M. F. Kugler J. Kroll H. Foss J. Ostwald P. (1999). Reducing myoclonus after etomidate. Anesthesiology 90 (1), 113–119. 10.1097/00000542-199901000-00017 9915320

[B17] Du X. Zhou C. Pan L. Li C. (2017). Effect of dexmedetomidine in preventing etomidate-induced myoclonus: a meta-analysis. Drug Des. Devel Ther. 11, 365–370. 10.2147/dddt.s121979 PMC5308599 28223779

[B18] Forman S. A. (2011). Clinical and molecular pharmacology of etomidate. Anesthesiology 114 (3), 695–707. 10.1097/ALN.0b013e3181ff72b5 21263301 PMC3108152

[B19] Fu X. Liu Z. Liu Y. Hu G. (2018). Tramadol pretreatment reduces myoclonus induced by etomidate. Med J Wuhan Univ. 39 (2), 4. 10.14188/j.1671-8852.2017.0434

[B20] González-Xuriguera C. G. Vergara-Merino L. Garegnani L. Ortiz-Muñoz L. Meza N. (2021). Introduction to network meta-analysis for evidence synthesis. Medwave 21 (6), e8315. 10.5867/medwave.2021.06.8315 34292922

[B21] Guler A. Satilmis T. Akinci S. B. Celebioglu B. Kanbak M. (2005). Magnesium sulfate pretreatment reduces myoclonus after etomidate. Anesth. Analg. 101 (3), 705–709. 10.1213/01.ane.0000160529.95019.e6 16115978

[B22] Gultop F. Akkaya T. Bedirli N. Gumus H. (2010). Lidocaine pretreatment reduces the frequency and severity of myoclonus induced by etomidate. J. Anesth. 24 (2), 300–302. 10.1007/s00540-010-0869-6 20108006

[B23] Gupta M. Gupta P. (2018a). Nalbuphine pretreatment for prevention of etomidate induced myoclonus: a prospective, randomized and double-blind study. J. Anaesthesiol. Clin. Pharmacol. 34 (2), 200–204. 10.4103/joacp.JOACP_210_16 30104829 PMC6066906

[B24] Gupta P. Gupta M. (2018b). Comparison of different doses of intravenous lignocaine on etomidate-induced myoclonus: a prospective randomised and placebo-controlled study. Indian J. Anaesth. 62 (2), 121–126. 10.4103/ija.IJA_563_17 29491517 PMC5827478

[B25] He L. Ding Y. Chen H. Qian Y. Li Z. (2014). Butorphanol pre-treatment prevents myoclonus induced by etomidate: a randomised, double-blind, controlled clinical trial. Swiss Med. Wkly. 144, w14042. 10.4414/smw.2014.14042 25317545

[B26] Higgins J. P. Thompson S. G. Deeks J. J. Altman D. G. (2003). Measuring inconsistency in meta-analyses. Bmj 327 (7414), 557–560. 10.1136/bmj.327.7414.557 12958120 PMC192859

[B27] Honar H. Riazi K. Homayoun H. Sadeghipour H. Rashidi N. Ebrahimkhani M. R. (2004). Ultra-low dose naltrexone potentiates the anticonvulsant effect of low dose morphine on clonic seizures. Neuroscience 129 (3), 733–742. 10.1016/j.neuroscience.2004.08.029 15541894

[B28] Hua J. Miao S. Shi M. Tu Q. Wang X. Liu S. (2019). Effect of butorphanol on etomidate-induced myoclonus: a systematic review and meta-analysis. Drug Des. Devel Ther. 13, 1213–1220. 10.2147/DDDT.S191982 PMC6489683 31114161

[B29] Hueter L. Schwarzkopf K. Simon M. Bredle D. Fritz H. (2003). Pretreatment with sufentanil reduces myoclonus after etomidate. Acta Anaesthesiol. Scand. 47 (4), 482–484. 10.1034/j.1399-6576.2003.00081.x 12694150

[B30] Hutton B. Salanti G. Caldwell D. M. Chaimani A. Schmid C. H. Cameron C. (2015). The PRISMA extension statement for reporting of systematic reviews incorporating network meta-analyses of health care interventions: checklist and explanations. Ann. Intern Med. 162 (11), 777–784. 10.7326/m14-2385 26030634

[B31] Hwang J. Y. Kim J. H. Oh A. Y. Do S. H. Jeon Y. T. Han S. H. (2008). A comparison of midazolam with remifentanil for the prevention of myoclonic movements following etomidate injection. J. Int. Med. Res. 36 (1), 17–22. 10.1177/147323000803600103 18230263

[B32] Kim W. J. Hwang T. H. Hwang T. H. (2020). Comparative efficacy and safety of pharmacological interventions for the treatment of COVID-19: a systematic review and network meta-analysis. PLoS Med. 17 (12), e1003501. 10.1371/journal.pmed.1003501 33378357 PMC7794037

[B33] Ko B. J. Oh J. N. Lee J. H. Choi S. R. Lee S. C. Chung C. J. (2013). Comparison of effects of fentanyl and remifentanil on hemodynamic response to endotracheal intubation and myoclonus in elderly patients with etomidate induction. Korean J. Anesthesiol. 64 (1), 12–18. 10.4097/kjae.2013.64.1.12 23372880 PMC3558642

[B34] Komatsu R. You J. Mascha E. J. Sessler D. I. Kasuya Y. Turan A. (2013). Anesthetic induction with etomidate, rather than propofol, is associated with increased 30-day mortality and cardiovascular morbidity after noncardiac surgery. Anesth. Analg. 117 (6), 1329–1337. 10.1213/ANE.0b013e318299a516 24257383

[B35] Kwon M. S. Kim J. H. Baik H. (2002). The effect of midazolam for reducing myoclonus after etomidate. Korean J. Anesthesiol. 43 (4), 395–400. 10.4097/kjae.2002.43.4.395

[B36] Lang B. Zhang L. Li F. Lin Y. Zhang W. Yang C. (2019). Comparison of the efficacy and safety of remifentanil versus different pharmacological approaches on prevention of etomidate-induced myoclonus: a meta-analysis of randomized controlled trials. Drug Des. Devel Ther. 13, 1593–1607. 10.2147/dddt.s200200 PMC6512956 31190739

[B37] Lang B. Zhang L. Yang C. Lin Y. Zhang W. Li F. (2018). Pretreatment with lidocaine reduces both incidence and severity of etomidate-induced myoclonus: a meta-analysis of randomized controlled trials. Drug Des. Devel Ther. 12, 3311–3319. 10.2147/DDDT.S174057 PMC6174893 30323563

[B38] Lee S. W. Gill H. J. Park S. C. Kim J. Y. Kim J. H. Lee J. Y. (2009). The effect of remifentanil for reducing myoclonus during induction of anesthesia with etomidate. Korean J. Anesthesiol. 57 (4), 438–443. 10.4097/kjae.2009.57.4.438 30625903

[B39] Loacker S. Sayyah M. Wittmann W. Herzog H. Schwarzer C. (2007). Endogenous dynorphin in epileptogenesis and epilepsy: anticonvulsant net effect via kappa opioid receptors. Brain 130 (Pt 4), 1017–1028. 10.1093/brain/awl384 17347252

[B40] Luan H. F. Zhao Z. B. Feng J. Y. Cui J. Z. Zhang X. B. Zhu P. (2015). Prevention of etomidate-induced myoclonus during anesthetic induction by pretreatment with dexmedetomidine. Braz J. Med. Biol. Res. 48 (2), 186–190. 10.1590/1414-431x20144100 25351237 PMC4321226

[B41] Lv Y. He H. Xie J. Jin W. Shou C. Pan Y. (2018). Effects of transcutaneous acupoint electrical stimulation combined with low-dose sufentanil pretreatment on the incidence and severity of etomidate-induced myoclonus: a randomized controlled trial. Med. Baltim. 97 (23), e10969. 10.1097/md.0000000000010969 PMC5999512 29879048

[B42] Ma T. Wang W. Li G. Sui B. Zhang Y. (2015). Effect of remifentanil pretreatment on myoclonus after etomidate injection. Chin. J. Postgrad. Med. 38 (2), 3. 10.3760/cma.j.issn.1673-4904.2015.02.007

[B43] Malay H. P. Rajesh C. Monal N. R. Seema G. (2015). A comparison of dexmedetomidine and midazolam for the prevention of myoclonic movements and pain following etomidate injection. Res. J. Pharm. Biol. Chem. Sci. 6 (5), 161–168.

[B44] Manocha A. Mediratta P. K. Sharma K. K. (2003a). Studies on the anticonvulsant effect of U50488H on maximal electroshock seizure in mice. Pharmacol. Biochem. Behav. 76 (1), 111–117. 10.1016/s0091-3057(03)00218-1 13679223

[B45] Manocha A. Sharma K. K. Mediratta P. K. (2003b). Possible mechanism involved in the anticonvulsant action of butorphanol in mice. Pharmacol. Biochem. Behav. 74 (2), 343–350. 10.1016/s0091-3057(02)01004-3 12479953

[B46] Miao S. Zou L. Wang G. Wang X. Liu S. Shi M. (2019). Effect of dexmedetomidine on etomidate-induced myoclonus: a randomized, double-blind controlled trial. Drug Des. Dev. Ther. 13, 1803–1808. 10.2147/DDDT.S194456 PMC6554000 31239638

[B47] Mizrak A. Koruk S. Bilgi M. Kocamer B. Erkutlu I. Ganidagli S. (2010). Pretreatment with dexmedetomidine or thiopental decreases myoclonus after etomidate: a randomized, double-blind controlled trial. J. Surg. Res. 159 (1), e11–e16. 10.1016/j.jss.2009.07.031 20018300

[B48] Morel J. Salard M. Castelain C. Bayon M. C. Lambert P. Vola M. (2011). Haemodynamic consequences of etomidate administration in elective cardiac surgery: a randomized double-blinded study. Br. J. Anaesth. 107 (4), 503–509. 10.1093/bja/aer169 21685487

[B49] Mullick P. Talwar V. Aggarwal S. Prakash S. Pawar M. (2018). Comparison of priming versus slow injection for reducing etomidate-induced myoclonus: a randomized controlled study. Korean J. Anesthesiol. 71 (4), 305–310. 10.4097/kja.d.18.27168 30071713 PMC6078874

[B50] Palmer S. C. Tendal B. Mustafa R. A. Vandvik P. O. Li S. Hao Q. (2021). Sodium-glucose cotransporter protein-2 (SGLT-2) inhibitors and glucagon-like peptide-1 (GLP-1) receptor agonists for type 2 diabetes: systematic review and network meta-analysis of randomised controlled trials. BMJ 372, m4573. 10.1136/bmj.m4573 33441402 PMC7804890

[B51] Qiu P. Qiu S. Dong Y. (2016). Effect of opiod pretreatment on etomidate induced myoclonus: a meta-analysis. J China Med Univ. 45 (4), 6. 10.12007/j.issn.0258⁃4646.2016.04.008

[B52] Rajkumar G. Shammy N. Thokchom R. S. Singh T. H. Dhayanithy M. Devi K. R. (2023). Dexmedetomidine versus lignocaine in the prevention of etomidate-induced myoclonus-A randomised double-blinded study. J. Clin. and Diagnostic Res. 17 (2). 10.7860/JCDR/2023/60034.17439

[B53] Rani A. Narwa Y. Arora G. (2023). Evaluating the comparative efficacy of various dosages of midazolam in preventing etomidate-induced myoclonus. A Hosp. based Study 15 (9), 834–841.

[B54] Rautela R. S. Gulabani M. Kumar P. Salhotra R. Mohta M. Verma K. (2023). Comparative assessment of dexmedetomidine and butorphanol for attenuation of etomidate-induced myoclonus: a double-blind, randomised controlled study. Indian J. Anaesth. 67 (9), 815–820. 10.4103/ija.ija_414_23 37829775 PMC10566664

[B55] Ren J. Lan P. Yuan R. (2013). Effect of butorphanol pretreatment on myoclonus induced by etomidate during general anesthesia. Shandong Med. J. 53 (48), 3.

[B56] Ren Y. Shi W. Chen C. Li H. Niu C. Zheng X. (2019). Effect of preinjection of Nalbuphine on Etomidate - induced myoclonus during induction of general anesthesia. CHINA Med. Her. 16 (25), 4.

[B57] Riley R. D. Dan J. Salanti G. Burke D. L. White I. R. (2017). Multivariate and network meta-analysis of multiple outcomes and multiple treatments: rationale, concepts, and examples. BMJ Clin. Res. 358, j3932. 10.1136/bmj.j3932 PMC5596393 28903924

[B58] Salanti G. (2012). Indirect and mixed-treatment comparison, network, or multiple-treatments meta-analysis: many names, many benefits, many concerns for the next generation evidence synthesis tool. Res. Synth. Methods 3 (2), 80–97. 10.1002/jrsm.1037 26062083

[B59] Salanti G. Ades A. E. Ioannidis J. P. (2011). Graphical methods and numerical summaries for presenting results from multiple-treatment meta-analysis: an overview and tutorial. J. Clin. Epidemiol. 64 (2), 163–171. 10.1016/j.jclinepi.2010.03.016 20688472

[B60] Sedighinejad A. Naderi Nabi B. Haghighi M. Biazar G. Imantalab V. Rimaz S. (2016). Comparison of the effects of low-dose midazolam, magnesium sulfate, remifentanil and low-dose etomidate on prevention of etomidate-induced myoclonus in orthopedic surgeries. Anesth. Pain Med. 6 (2), e35333. 10.5812/aapm.35333 27247915 PMC4885461

[B61] Shan G. Lu H. Dai F. Liu Y. Yin D. Cao H. (2023). Low-dose nalmefene pretreatment reduces etomidate-induced myoclonus: a randomized, double-blind controlled trial. Medicine 102 (36), e35138. 10.1097/MD.0000000000035138 37682124 PMC10489433

[B62] Siddiqui A. Agrawal A. Shaikh M. Siddiqui F. (2023a). Efficacy of nalbuphine pre-treatment in attenuation of etomidate induced myoclonus: a placebo controlled trial from Malwa region. J. Cardiovasc. Dis. Res. 14 (5), 1385–1389.

[B63] Siddiqui A. Dhamnani R. Sinha J. Mandloi P. (2023b). Comparison between fentanyl and nalbuphine pretreatment in prevention of etomidate induced myoclonus. Int. J. Pharm. Clin. Res. 15 (4), 1137–1143.

[B64] Singh K. Ruchi G. Singh A. Kaur B. (2014). Efficacy of lignocaine versus midazolam in controlling etomidate-induced myoclonus: a randomized placebo-controlled study. Ain-Shams J. Anaesthesiol. 7 (3), 460. 10.4103/1687-7934.139597

[B65] Sivilotti M. L. Filbin M. R. Murray H. E. Slasor P. Walls R. M. NEAR Investigators (2003). Does the sedative agent facilitate emergency rapid sequence intubation? Acad. Emerg. Med. 10 (6), 612–620. 10.1111/j.1553-2712.2003.tb00044.x 12782521

[B66] Staahl C. Christrup L. L. Andersen S. D. Arendt-Nielsen L. Drewes A. M. (2006). A comparative study of oxycodone and morphine in a multi-modal, tissue-differentiated experimental pain model. Pain 123 (1-2), 28–36. 10.1016/j.pain.2006.02.006 16600508

[B67] Sterne J. A. Sutton A. J. Ioannidis J. P. Terrin N. Jones D. R. Lau J. (2011). Recommendations for examining and interpreting funnel plot asymmetry in meta-analyses of randomised controlled trials. Bmj 343, d4002. 10.1136/bmj.d4002 21784880

[B68] Sterne J. A. C. Savović J. Page M. J. Elbers R. G. Blencowe N. S. Boutron I. (2019). RoB 2: a revised tool for assessing risk of bias in randomised trials. Bmj 366, l4898. 10.1136/bmj.l4898 31462531

[B69] Un B. Ceyhan D. Yelken B. (2011). Prevention of etomidate-related myoclonus in anesthetic induction by pretreatment with magnesium. J. Res. Med. Sci. Official J. Isfahan Univ. Med. Sci. 16 (11), 1490–1494. PMC3430068 22973352

[B70] Wang J. Li Q. B. Wu Y. Y. Wang B. N. Kang J. L. Xu X. W. (2018a). Efficacy and safety of opioids for the prevention of etomidate-induced myoclonus: a meta-analysis. Am. J. Ther. 25 (5), e517–e523. 10.1097/mjt.0000000000000404 26840341

[B71] Wang L. Wang Z. (2018). Effect of pretreatment with low dose Dexmedetomidine on Etomidate induced myoclonus in short-time surgery. Chin. J. Mod. Drug Appl. 12 (17), 3. 10.14164/j.cnki.cn11-5581/r.2018.17.005

[B72] Wang W. Lv J. Qian Y. Yu W. (2015). Effect of oxycodone or fentanyl on myoclonus induced by etomidate. J. Clin. Anesthesiol. 31 (7), 2.

[B73] Wang W. Lv J. Wang Q. Yang L. Yu W. (2018b). Oxycodone for prevention of etomidate-induced myoclonus: a randomized double-blind controlled trial. J. Int. Med. Res. 46 (5), 1839–1845. 10.1177/0300060518761788 29536782 PMC5991229

[B74] Wu G. N. Xu H. J. Liu F. F. Xian W. Zhou H. (2016). Low-dose ketamine pretreatment reduces the incidence and severity of myoclonus induced by etomidate: a randomized, double-blinded, controlled clinical trial. Medicine 95 (6), e2701. 10.1097/MD.0000000000002701 26871805 PMC4753901

[B75] Xie Y. Song D. Zhou Y. Liu G. (2016). Preventive effect of fentanyl and remifentanil on myoclonus induced by etomidate in elderly patients. Pract. Pharm. And Clin. Remedies 19 (2), 3. 10.14053/j.cnki.ppcr.201602015

[B76] Yan P. Xisheng S. Fuhai J. Ju Q. (2022). Infuence of low dose propofol prejection on the incidence of etomidate induced myoclonus during general anesthesia in elderly patients. Chin. J. Hemorheol. 32(2). 10.3969/j.issn.1009-881X.2022.02.005

[B77] Yu H. Fang L. Du R. Zhang W. Zhu D. (2009). Efficacy of pretreatment of vecuronium combined with dilution of etomidate on etomidate-induced myoclonus. WEST CHINA Med. J. 24 (8), 4.

[B78] Zhang J. Liu L. Liu H. Lv G. (2015a). Comparison of butorphanol or midazolam alone and combination of the two drugs in preventing etomidate-induced myoclonus during anesthesia induction. Chin. J. Anesthesiol. 35 (11), 1325–1327. 10.3760/cma.j.issn.0254-1416.2015.11.010

[B79] Zhang J. Liu L. Lv G. (2015b). Comparison of the effects of intravenous pre-treatment of Butorphanol and Dezocine on prevention of Etomidate-induced myoclonus. Tianjin Med. J. 43 (12), 1450–1453. 10.11958/j.issn.0253-9896.2015.12.028

[B80] Zhang K. D. Wang L. Y. Zhang D. X. Zhang Z. H. Wang H. L. (2022). Comparison of the effectiveness of various drug interventions to prevent etomidate-induced myoclonus: a bayesian network meta-analysis. Front. Med. (Lausanne) 9, 799156. 10.3389/fmed.2022.799156 35559341 PMC9086535

[B81] Zhang N. Zhang A. Wang R. Chen J. (2021). Effect of dexmedetomidine and lidocaine pretreatment on adverse reactions of etomidate during general anesthesia induction. Anhui Med Phar J 25 (1), 4. 10.3969/j.issn.1009-6469.2021.01.038

[B82] Zhou C. Zhu Y. Liu Z. Ruan L. (2017). Effect of pretreatment with midazolam on etomidate-induced myoclonus: a meta-analysis. J. Int. Med. Res. 45 (2), 399–406. 10.1177/0300060516682882 28415947 PMC5536644

[B83] Zhu Y. Yang Y. Zhou C. Bao Z. (2017). Using dezocine to prevent etomidate-induced myoclonus: a meta-analysis of randomized trials. Drug Des. Devel Ther. 11, 2163–2170. 10.2147/dddt.s137464 PMC5522665 28761332

[B84] Zhu Y. Zhou C. He Q. (2019). Butorphanol effectively prevents etomidate-induced myoclonus: a pooled analysis of 788 patients. Gut Liver 47 (1), 353–360. 10.1177/0300060518801457 PMC6384487 30497306

