# Supplementary material for: Comparative efficacy and safety of 20 intravenous pharmaceutical intervention for prevention of etomidate-induced myoclonus: a systematic review and Bayesian network meta-analysis
Source: Front Pharmacol. 2025 Jan 23;15:1507616. doi: 10.3389/fphar.2024.1507616 (PMC11799762; doi:10.3389/fphar.2024.1507616)
Supplement: Supplementary file 1 [file DataSheet1.pdf]

Supplementary Materials

Table S1 PRISMA NMA Checklist of Items to Include When Reporting a Systematic Review Involving a Network Meta-analysis

Section/Topic	Item #	Checklist Item	Reported on Page #
**TITLE**			
Title	1	Identify the report as a systematic review *incorporating a network meta-analysis (or related form of meta-analysis).*	1
**ABSTRACT**			
Structured summary	2	Provide a structured summary including, as applicable:<br>**Background:** main objectives<br>**Methods:** data sources; study eligibility criteria, participants, and interventions; study appraisal; and *synthesis methods, such as network meta-analysis.*<br>**Results:** number of studies and participants identified; summary estimates with corresponding confidence/credible intervals; *treatment rankings may also be discussed. Authors may choose to summarize pairwise comparisons against a chosen treatment included in their analyses for brevity.*<br>**Discussion/Conclusions:** limitations; conclusions and implications of findings.<br>**Other:** primary source of funding; systematic review registration number with registry name.	1-2
**INTRODUCTION**			
Rationale	3	Describe the rationale for the review in the context of what is already known*, including mention of why a network meta-analysis has been conducted.*	3
Objectives	4	Provide an explicit statement of questions being addressed, with reference to participants, interventions, comparisons, outcomes, and study design (PICOS).	3
**METHODS**			
Protocol and registration	5	Indicate whether a review protocol exists and if and where it can be accessed (e.g., Web address); and, if available, provide registration information, including registration number.	4
Eligibility criteria	6	Specify study characteristics (e.g., PICOS, length of follow-up) and report characteristics (e.g., years considered, language, publication status) used as criteria for eligibility, giving rationale. *Clearly describe eligible treatments included in the treatment network, and note whether any have been clustered or merged into the same node (with justification).*	4-5

Information sources	7	Describe all information sources (e.g., databases with dates of coverage, contact with study authors to identify additional studies) in the search and date last searched.	5
Search	8	Present full electronic search strategy for at least one database, including any limits used, such that it could be repeated.	Supplementary Material page 5-7
Study selection	9	State the process for selecting studies (i.e., screening, eligibility, included in systematic review, and, if applicable, included in the meta-analysis).	4-5
Data collection process	10	Describe method of data extraction from reports (e.g., piloted forms, independently, in duplicate) and any processes for obtaining and confirming data from investigators.	5
Data items	11	List and define all variables for which data were sought (e.g., PICOS, funding sources) and any assumptions and simplifications made.	6
**Geometry of the network**	**S1**	Describe methods used to explore the geometry of the treatment network under study and potential biases related to it. This should include how the evidence base has been graphically summarized for presentation, and what characteristics were compiled and used to describe the evidence base to readers.	6,8
Risk of bias within individual studies	12	Describe methods used for assessing risk of bias of individual studies (including specification of whether this was done at the study or outcome level), and how this information is to be used in any data synthesis.	6
Summary measures	13	State the principal summary measures (e.g., risk ratio, difference in means). *Also describe the use of additional summary measures assessed, such as treatment rankings and surface under the cumulative ranking curve (SUCRA) values, as well as modified approaches used to present summary findings from meta-analyses.*	6
Planned methods of analysis	14	Describe the methods of handling data and combining results of studies for each network meta-analysis. This should include, but not be limited to:<br>• *Handling of multi-arm trials;*<br>• *Selection of variance structure;*<br>• *Selection of prior distributions in Bayesian analyses; and*<br>• *Assessment of model fit.*	6-7
**Assessment of Inconsistency**	**S2**	Describe the statistical methods used to evaluate the agreement of direct and indirect evidence in the treatment network(s) studied. Describe efforts taken to address its presence when found.	6
Risk of bias across studies	15	Specify any assessment of risk of bias that may affect the cumulative evidence (e.g., publication bias, selective reporting within studies).	7
Additional analyses	16	Describe methods of additional analyses if done, indicating which were pre-specified. This may include, but not be limited to, the following:<br>• Sensitivity or subgroup analyses;<br>• Meta-regression analyses;	7

- *Alternative formulations of the treatment network; and*
- *Use of alternative prior distributions for Bayesian analyses (if applicable).*

RESULTS†

Study selection	17	Give numbers of studies screened, assessed for eligibility, and included in the review, with reasons for exclusions at each stage, ideally with a flow diagram.	7
**Presentation of network structure**	**S3**	Provide a network graph of the included studies to enable visualization of the geometry of the treatment network.	8
**Summary of network geometry**	**S4**	Provide a brief overview of characteristics of the treatment network. This may include commentary on the abundance of trials and randomized patients for the different interventions and pairwise comparisons in the network, gaps of evidence in the treatment network, and potential biases reflected by the network structure.	7-8
Study characteristics	18	For each study, present characteristics for which data were extracted (e.g., study size, PICOS, follow-up period) and provide the citations.	Supplementary Material page 8-14
Risk of bias within studies	19	Present data on risk of bias of each study and, if available, any outcome level assessment.	Supplementary Material page 15-16
Results of individual studies	20	For all outcomes considered (benefits or harms), present, for each study: 1) simple summary data for each intervention group, and 2) effect estimates and confidence intervals. *Modified approaches may be needed to deal with information from larger networks.*	9-13
Synthesis of results	21	Present results of each meta-analysis done, including confidence/credible intervals. *In larger networks, authors may focus on comparisons versus a particular comparator (e.g. placebo or standard care), with full findings presented in an appendix. League tables and forest plots may be considered to summarize pairwise comparisons.* If additional summary measures were explored (such as treatment rankings), these should also be presented.	Supplementary Material page 17-28
**Exploration for inconsistency**	**S5**	Describe results from investigations of inconsistency. This may include such information as measures of model fit to compare consistency and inconsistency models, *P* values from statistical tests, or summary of inconsistency estimates from different parts of the treatment network.	9-13
Risk of bias across studies	22	Present results of any assessment of risk of bias across studies for the evidence base being studied.	8<br>Supplementary Material page 41

Results of additional analyses	23	Give results of additional analyses, if done (e.g., sensitivity or subgroup analyses, meta-regression analyses*, alternative network geometries studied, alternative choice of prior distributions for Bayesian analyses,* and so forth).	13
**DISCUSSION**			
Summary of evidence	24	Summarize the main findings, including the strength of evidence for each main outcome; consider their relevance to key groups (e.g., healthcare providers, users, and policy-makers).	14-16
Limitations	25	Discuss limitations at study and outcome level (e.g., risk of bias), and at review level (e.g., incomplete retrieval of identified research, reporting bias). *Comment on the validity of the assumptions, such as transitivity and consistency. Comment on any concerns regarding network geometry (e.g., avoidance of certain comparisons).*	17
Conclusions	26	Provide a general interpretation of the results in the context of other evidence, and implications for future research.	17
**FUNDING**			
Funding	27	Describe sources of funding for the systematic review and other support (e.g., supply of data); role of funders for the systematic review. This should also include information regarding whether funding has been received from manufacturers of treatments in the network and/or whether some of the authors are content experts with professional conflicts of interest that could affect use of treatments in the network.	17

PICOS = population, intervention, comparators, outcomes, study design.
* Text in italics indicates wording specific to reporting of network meta-analyses that has been added to guidance from the PRISMA statement.

Table S2 Literature search strategy

PubMed

#1	etomidate [mesh]
#2	etomidate [text word]
#3	#1 OR #2
#4	myoclonus[mesh]
#5	myoclonus [text word]
#6	myoclonic movement [text word]
#7	#4 OR #5 OR #6
#8	randomized controlled trial [pt]
#9	controlled clinical trial [pt]
#10	randomized [tiab]
#11	placebo [tiab]
#12	clinical trials [mesh major topic]
#13	randomly [tiab]
#14	trial [ti]
#15	#8 OR #9 OR #10 OR #11 OR #12 OR #13 OR #14
#16	animals [mh] NOT humans [mh]
#17	#15 NOT #16
#18	#3 AND #7 AND #17

Embase

#1	'etomidate'/exp
#2	etomidate [ab, ti]
#3	etomidate
#4	#1 OR #2 OR #3
#5	'myoclonus'/exp
#6	myoclonus [ab, ti]
#7	myoclonus
#8	myoclonic movement [ab, ti]
#9	#5 OR #6 OR #7 OR #8
#10	randomized controlled trial [pt]
#11	controlled clinical trial [pt]
#12	randomized [ab,ti]
#13	placebo [ab,ti]
#14	clinical trials [exp]
#15	randomly [ab,ti]
#16	trial [ti]
#17	#10 OR #11 OR #12 OR #13 OR #14 OR #15 OR #16
#18	#4 AND #9 AND #17 AND humans/lim

Cochrane

#1	etomidate [explode all trees]
#2	etomidate[ti,ab,kw]
#3	etomidate
#4	#1 OR #2 OR #3
#5	myoclonus [explode all trees]
#6	myoclonus[ti,ab,kw]
#7	myoclonus
#8	myoclonic movement[ti,ab,kw]
#9	myoclonic movement
#10	#5 OR #6 OR #7 OR #8 OR #9
#11	randomized controlled trial [pt]
#12	controlled clinical trial [pt]
#13	randomized [ti,ab,kw]
#14	placebo [ti,ab,kw]
#15	clinical trials [ti,ab,kw]
#16	randomly [ti,ab,kw]
#17	#11 OR #12 OR #13 OR #14 OR #15 OR #16
#18	#4 AND #10 AND #17

Table S3 Basic characteristics of included studies

Study Year, country	Type of surgery	ASA-physical status	Gender T/C, Female/ %	Cases T/C	Age Mean ± SD T/C	Etomidate induction	Observation time	Active and control group	Reported outcomes
Miao [1] 2019, China	elective surgery	I-II	40%/ 34%	50/ 50	47.6±6.3/ 49.8±7.6	0.3mg/kg	3min	active group: dexmedetomidine 0.5 µg/kg; control group: NS	The risk and intensity of EIM. AEs.
Luan [2] 2015, China	elective surgery	I-II	57%/ 50%/ 47%	30/ 30/ 30	43±11/ 40±10/ 41±9	0.3mg/kg	1min	active group: dexmedetomidine 0.5 µg/kg; dexmedetomidine 1.0 µg/kg; control group: NS	The risk and intensity of EIM. AEs.
Patel [3] 2015, India	elective surgery	I-II	33%/ 37% 37%	30/ 30/ 30	34.8±11.16/ 34.0±11.24/ 38.43±11.85	0.3mg/kg	1min	active group: dexmedetomidine 1.0 µg/kg; midazolam 0.05 mg/kg; control group: NS	The risk and intensity of EIM. AEs.
Mizrak [4] 2010, Turkey	elective surgery	I-II	57%/ 63% 73%	30/ 30/ 30	42±13/ 37±11/ 40±15	0.3mg/kg	NG	active group: dexmedetomidine 0.5 µg/kg; thiopental 1mg/kg; control group: NS	The risk and intensity of EIM. AEs.
Wang [5] 2018[a], China	elective surgery	I-II	100%	15/ 15	39±11/37±9	0.3mg/kg	1min	active group: dexmedetomidine 0.2 µg/kg; control group: NS	The risk and intensity of EIM.
Chen [6] 2016, China	elective surgery	II-III	NG	30/ 30/ 30	65-85	0.2mg/kg	1min	active group: dexmedetomidine 0.2 µg/kg; dexmedetomidine 0.4 µg/kg; control group: NS	The risk and intensity of EIM. AEs.
Zhang [7] 2021, China	elective surgery	I-II	48%/ 46%/ 50%	48/ 48/ 48	41.5±8.4/ 40.2±8.5/ 49.1±9.5	0.3mg/kg	2min	active group: dexmedetomidine 0.5 µg/kg; lidocaine 1mg/kg; control group: NS	The risk and intensity of EIM. AEs.
Kwon [8] 2002, Korea	elective surgery	I-II	40%/ 40%	40/ 40	41.4 ± 10.7/ 40.4 ± 10.9	0.3mg/kg	2min	active group: midazolam 0.04 mg/kg； control group: NS	The risk and intensity of EIM. duration of myoclonus
Alipour [9] 2016, Iran	elective eye surgery	II-III	60%/ 32%	25/ 25	61.16±11.84/ 61.16±15.00	0.3mg/kg	2min	active group: sufentanil 0.2 µg/kg; midazolam 0.015 mg/kg	The frequency, intensity and duration of EIM
Lv [10]	elective	I-II	NG	43/	34.3±8.3/	0.3mg/kg	2min	active group:	The risk and intensity of

2018, China	hysteroscopy			43	33.2±6.9			sufentanil 0.1μg/kg;<br>control group: NS	EIM. AEs.
Ko [11]<br>2013, Korea	elective surgery	I-II	50%/<br>47%/<br>50%	30/<br>30/<br>30	68.9±4.6/<br>69.4±4.0/<br>69.6±4.5	0.2mg/kg	NG	active group:<br>remifentanil 1.0 μg/kg;<br>fentanyl 1.0 μg/kg;<br>control group: NS	The risk and intensity of EIM. AEs.
Lee [12]<br>2009, Korea	elective plastic surgery	I	50%/<br>47%/<br>53%	30/<br>30/<br>30	32.4±6.9/<br>34.5±13.1/<br>36.7±11.7	0.3mg/kg	NG	active group:<br>remifentanil 0.5 μg/kg;<br>remifentanil 1.0 μg/kg;<br>control group: NS	The risk, duration and intensity of EIM.
Ma [13]<br>2015, China	elective surgery	I-II	47%/<br>47%	30/<br>30	42.1±10.1/<br>42.2±15.2	0.3mg/kg	NG	active group:<br>remifentanil 0.5 μg/kg;<br>control group: NS	The risk and intensity of EIM
Hwang [14]<br>2008, Korea	elective surgery	I-II	NG	29/<br>30/<br>30	51±11/<br>52±12/<br>50±12	0.3mg/kg	2min	active group:<br>remifentanil 1.0 μg/kg;<br>midazolam 0.05 mg/kg;<br>control group: NS	The risk and intensity of EIM. AEs.
Xie [15]<br>2016, China	elective surgery	I-II	53%/<br>47%/<br>50%	30/<br>30/<br>30	72.13±4.35/<br>72.26±3.84/<br>71.33±5.38	0.2mg/kg	NG	active group:<br>fentanyl 3.0 μg/kg;<br>remifentanil 2.0 μg/kg;<br>control group: NS	The risk and intensity of EIM.
Chen [16]<br>2009, China	elective surgery	I-II	73%/<br>67%/<br>73%/<br>60%	15/<br>15/<br>15/<br>15	45±10/<br>48±11/<br>46±11/<br>40±13/	0.3mg/kg	1min	active group:<br>fentanyl 2.0 μg/kg;<br>remifentanil 1.0 μg/kg;<br>sufentanil 0.3 μg/kg;<br>control group: NS	The risk and intensity of EIM.
Wang [17]<br>2015, China	elective surgery	I-II	48%/<br>46%/<br>52%	56/<br>56/<br>56	49.2±7.7/<br>45.7±9.6/<br>48.3±8.9	0.3mg/kg	2min	active group:<br>fentanyl 1.0 μg/kg;<br>oxycodone 0.1 mg/kg;<br>control group: NS	The risk and intensity of EIM. AEs.
Chen [18]<br>2017, China	elective surgery	I-II	50%/<br>48%	48/<br>48	49.2±9.5/<br>40.3±8.6	0.3mg/kg	1min	active group:<br>sufentanil 0.3 μg/kg;<br>control group: NS	The risk and intensity of EIM. AEs.
Wang [19]<br>2018[b], China	elective surgery	I-II	46%/<br>48%/<br>54%	54/<br>54/<br>54	45.4±10.6/<br>46.4±11.5/<br>48.3±9.7	0.3mg/kg	2min	active group:<br>fentanyl 1.0 μg/kg;<br>oxycodone 0.1 mg/kg,<br>control group: NS	The risk and intensity of EIM. AEs.
An [20]<br>2017, China	elective surgery	I-II	62%/<br>60%	60/<br>60	53±9/<br>52±10	0.3mg/kg	2min	active group:<br>oxycodone 0.05 mg/kg;<br>control group: NS	The risk and intensity of EIM. AEs.

Cho [21] 2008, Korea	elective surgery	I-II	53%/ 47%/ 53%/ 53%	19/ 19/ 19/ 19	42.0 ± 14.0/ 39.0 ± 12.0/ 43.0 ±10.0/ 41.0 ± 16.0	0.3mg/kg	2min	active group: alfentanil 2.5 μg/kg; alfentanil 5.0 μg/kg; alfentanil 10.0 μg/kg; control group: NS	The risk, intensity, and duration of EIM. AEs.
Singh [22] 2014, India	elective surgery	I-II	92%/ 80%/ 92%	25/ 25/ 25	40.04±12.17/ 41.40±11.74/ 35.36±11.94	0.3mg/kg	1min	active group: 1 ml of 2% lidocaine (20mg); 1ml of midazolam (1 mg); control group: NS	The risk, intensity, and duration of EIM. AEs.
Gupta [23] 2018[a], Iran	elective surgery	I-II	62%/ 56%/ 58%/ 62%	50/ 50/ 50/ 50	39.12±11.52/ 38.74±13.05/ 38.98±12.43/ 40.2±11.796	0.3mg/kg	2min	active group: lidocaine 0.5 mg/kg; lidocaine 1.0 mg/kg; lidocaine 1.5 mg/kg; control group: NS	The risk of myoclonus at 1 min (EM1), 2 min (EM2), and intensity of EIM.
Gultop [24] 2010, Turkey	elective orthopedic and general surgical procedures	I-II	43%/ 53%	30/ 30	51.5±13/ 53.7±11	0.3mg/kg	1min	active group: 1 ml of 2% lidocaine (20mg); control group: NS	The risk and intensity of EIM.
He [25] 2014, China	elective surgery	I-II	57%/ 52%	54/ 54	44.8±11.0/ 47.3±12.5	0.3mg/kg	2min	active group: butorphanol 0.015 mg/kg; control group: NS	The risk and intensity of EIM. AEs.
Zhang [26] 2015[a], China	elective surgery	I-II	54%/ 58%	50/ 50	51.3±6.8/ 53.2±6.5	0.3mg/kg	2min	active group: butorphanol 0.015 mg/kg; control group: NS	The risk and intensity of EIM.
Ren [27] 2013, China	elective surgery	I-II	NG	50/ 50	20-65	0.3mg/kg	2min	active group: butorphanol 0.015 mg/kg; control group: NS	The risk and intensity of EIM.
Zhang [28] 2015[b], China	elective surgery	I-II	50%/ 47%/ 42%/	40/ 40/ 40	53±7/ 52±6/ 51±8	0.3mg/kg	2min	active group: butorphanol 0.015 mg/kg; midazolam 50 μg/kg; control group: NS	The risk and intensity of EIM.
Wu [29] 2016, China	elective surgery	I-II	50%/ 46%	52/ 52	41.6±13.0/ 44.3±14.2	0.3mg/kg	2min	active group: ketamine 0.5 mg/kg; control group: NS	The risk and intensity of EIM. AEs.
Guler [30] 2005, Turkey	elective surgery	I-III	56%/ 44%/ 48%/ 28%	25/ 25/ 25/ 25	42±14/ 48±19/ 50±18/ 51±18	0.2mg/kg	1min	active group: ketamine 0.2 mg/kg; ketamine 0.5 mg/kg; magnesium sulfate 2.48 mmol; control group: NS	The risk and intensity of EIM. AEs.

Un [31] 2011, Turkey	elective transurethral resection of prostate surgery	I-II	46%/ 44%	50/ 50	34.5±9.3/ 32.5±9.9	0.3mg/kg	1min	active group: magnesium sulphate 2.48 mmol; control group: NS	The risk of EIM.
Sedighinejad [32] 2016, Iran	elective orthopedic surgery	I-II	28%/ 22%/ 32%/ 18%	71/ 71/ 71/ 71	36.73±11.68/ 36.78±11.56/ 38.95±12.25/ 35.21±11.58	0.3mg/kg	1.5min	active group: midazolam 0.015 mg/kg; magnesium sulfate 30 mg/kg; remifentanil 1.0 μg/kg; low-dose etomidate 0.03 mg/kg	The risk and intensity of EIM
Aissaoui [33] 2006, Maroc	abdominal elective surgery	I-II	61%/ 52%	23/ 23	46±14/ 48±11	0.3mg/kg	1min	active group: low-dose etomidate 0.03 mg/kg; control group: NS	The risk and intensity of EIM. AEs.
Ren [34] 2019, China	Urological Surgery	I-II	56%/ 37%	32/ 32	44±9/ 45±9	0.3mg/kg	2min	active group: nalbuphine 0.2 mg/kg; control group: NS	The risk and intensity of EIM. AEs.
Gupta [35] 2018[b], India	elective surgery	I-II	80%/ 74%	50/ 50	42.3±13.4/ 39.6±13.2	0.3mg/kg	2min	active group: nalbuphine 0.2 mg/kg; control group: NS	The risk and intensity of EIM. AEs.
Choi [36] 2008, Korea	elective cardiac or pulmonary surgery	I-III	41%/ 37%	56/ 54	53.9±14.2/ 53.9±11.8	0.3mg/kg	1min	active group: rocuronium 0.06mg/kg; control group: NS	The risk and intensity of EIM. AEs.
Mullick [37] 2018, India	elective surgery	I-II	60%/ 59%	63/ 63	34.6 ±10.8/ 34.1 ±13.1	0.3mg/kg	3min	active group: low-dose etomidate 0.03 mg/kg; control group: No intervention	The risk and intensity of EIM
Fu [38] 2018, China	elective surgery	I-II	50%/ 57%/ 53%	30/ 30/ 30	45±15/ 47±17/ 45±15	0.3mg/kg	1min	active group: tramadol 1.0 mg/kg; tramadol 2.0 mg/kg; control group: NS	The risk and intensity of EIM. AEs.
Yu [39] 2009, China	elective surgery	I-II	45%/ 45%	20/ 20	51.2±14.7/ 51.5±12.1	0.3mg/kg	2min	active group: vecuronium 0.01 mg/kg; control group: NS	The risk and intensity of EIM. AEs.
Rautela [40] 2023, India	elective surgery	I-II	83%/ 80%	30/ 30	31.90±12.87/ 33.77±11.64	0.3mg/kg	1min	active group: butorphanol 0.015 mg/kg; dexmedetomidine 0.5 μg/kg	The risk and intensity of EIM.
Alipour [41] 2023, Iran	elective surgery	I-II	53%/ 62%/ 53%	32/ 32/ 32	39.25±12.03/ 39.25±1.53/ 38.63±10.61	0.3mg/kg	2min	active group: granisetron 40 μg/kg; sufentanil 0.2 μg/kg; control group: NS	The risk and intensity of EIM.

Shan [42] 2023, China	laparoscopic cholecystectomy	I-II	62%/ 72%/ 72%	50/ 50/ 50	54.6±11.4/ 55.1±12.9/ 57.0±10.7	0.3mg/kg	3min	active group: nalmefene 0.25 ug/kg; fentanyl 1.0 µg/kg; control group: NS	The risk and intensity of EIM. AEs.
Peng [43] 2022, China	elective surgery	I-II	36%/ 33%	45/ 45	69.0±5.2/ 66.7±4.9	0.3mg/kg	3min	active group: low-dose propofol 0.2 mg/kg; control group: NS	The risk and intensity of EIM. AEs.
Siddiqui [44] 2023[a], India	elective surgery	I-II	NR	30/30	39.80±10.68/ 42.1±9.63	0.3mg/kg	2min	active group: fentanyl 2.0 µg/kg; nalbuphine 0.2 mg/kg	The risk and intensity of EIM. AEs.
Rajkumar [45] 2023, India	upper abdominal surgery	I-II	71%/ 58%	52/ 52	35.52±11.73/ 36.63±11.49	0.3mg/kg	1.5min	active group: dexmedetomidine 0.5 µg/kg; lidocaine 1.0 mg/kg	The risk and intensity of EIM
Siddiqui [46] 2023[b], India	elective surgery	I-II	33%/ 37%	30/ 30	42.10±9.63/ 39.80±10.68	0.3mg/kg	2min	active group: nalbuphine 0.2 mg/kg; control group: NS	The risk and intensity of EIM. AEs.
Rani [47] 2023, India	elective surgery	I-II	78%/ 46%/ 73%/ 61%	41/ 41/ 41/ 41	31.71±9.41/ 31.91±9.91/ 34.21±11.21/ 34.51±12.51	0.3mg/kg	1min	active group: midazolam 0.015 mg/kg; midazolam 0.03 mg/kg; midazolam 0.05mg/kg; control group: NS	The risk and intensity of EIM
Agrawal [48] 2023, India	elective surgery	I-II	47.2%/ 45.3%	53/ 53	36.83±13.86/ 39.50±12.72	0.3mg/kg	2min	active group: lidocaine 1.5mg/kg; dexmedetomidine 1.0ug/kg	The risk and intensity of EIM. AEs.

ASA: American Society of Anesthesiologists; NS: normal saline; SD: standard deviation; AEs.: adverse events; NG: Not given. EIM: etomidate-induced myoclonus.

References:

1. Miao S, Zou L, Wang G, et al. Effect of dexmedetomidine on etomidate-induced myoclonus: a randomized, double-blind controlled trial. *Drug Des. Devel. Ther.* 2019; 13.
2. Luan HF, Zhao ZB, Feng JY, et al. Prevention of etomidate-induced myoclonus during anesthetic induction by pretreatment with dexmedetomidine. *Braz. J. Med. Biol. Res.* 2015; 48, 186-190.
3. Malay HP, Rajesh C, Monal NR, et al. A comparison of dexmedetomidine and midazolam for the prevention of myoclonic movements and pain following etomidate injection. *Research Journal of Pharmaceutical, Biological and Chemical Sciences* 2015; 6, 161-168.
4. Mizrak A, Koruk S, Bilgi M, et al. Pretreatment with dexmedetomidine or thiopental decreases myoclonus after etomidate: a randomized, double-blind controlled trial. *J. Surg. Res.* 2010; 159, e11-e16.
5. Wang L, Wang Z. Effect of pretreatment with low dose Dexmedetomidine on Etomidate induced myoclonus in short-time surgery *Chin J Mod Drug Appl* 2018; 12, 3.
6. Chen Z, Cheng C, Li D, et al. Efficacy of dexmedetomidine in reducing etomidate-induced myoclonus. *Jiangsu Med J* 2016; 42, 3.
7. Zhang N, Zhang A, Wang R, et al. Effect of dexmedetomidine and lidocaine pretreatment on adverse reactions of etomidate during general anesthesia induction. *Anhui Med and Phar J* 2021; 25, 4.
8. Kwon MS, Kim JH, Baik H. The Effect of Midazolam for Reducing Myoclonus after Etomidate. *Korean J. Anesthesiol.* 2002; 43, 395-400.

9. Alipour M, Tabari M, Azad AM. Comparative study evaluating efficacy of sufentanil versus midazolam in preventing myoclonic movements following etomidate. *Journal of Anaesthesiology Clinical Pharmacology* 2016; 32, 29-32.
10. Lv Y, He H, Xie J, et al. Effects of transcutaneous acupoint electrical stimulation combined with low-dose sufentanil pretreatment on the incidence and severity of etomidate-induced myoclonus: A randomized controlled trial. *Medicine (Baltimore)* 2018; 97, e10969.
11. Ko BJ, Oh JN, Lee JH, et al. Comparison of effects of fentanyl and remifentanil on hemodynamic response to endotracheal intubation and myoclonus in elderly patients with etomidate induction. *Korean J. Anesthesiol.* 2013; 64, 12-18.
12. Lee SW, Gill HJ, Park SC, et al. The effect of remifentanil for reducing myoclonus during induction of anesthesia with etomidate. *Korean J. Anesthesiol.* 2009; 57, 438-443.
13. Ma T, Wang W, Li G, et al. Effect of remifentanil pretreatment on myoclonus after etomidate injection. *Chin J Postgrad Med* 2015; 38, 3.
14. Hwang JY, Kim JH, Oh AY, et al. A comparison of midazolam with remifentanil for the prevention of myoclonic movements following etomidate injection. *J. Int. Med. Res.* 2008; 36, 17.
15. Xie Y, Song D, Zhou Y, et al. Preventive effect of fentanyl and remifentanil on myoclonus induced by etomidate in elderly patients. *Practical Pharmacy And Clinical Remedies* 2016; 19, 3.
16. Chen Y, Wang G, Yu Y. Effects of Different Opioids on Myoclonus and Bispectral Index during Etomidate Induction. *Tianjin Med J* 2009; 37, 3.
17. Wang W, Lv J, Qian Y, et al. Effect of oxycodone or fentanyl on myoclonus induced by etomidate. *J Clin Anesthesiol* 2015; 31, 2.
18. Chen L, Huang H, Xiong L, et al. Effect of sufentanil administration on myoclonus induced by etomidate. *Guangxi Med J* 2017; 39, 2.
19. Wang W, Lv J, Wang Q, et al. Oxycodone for prevention of etomidate-induced myoclonus: a randomized double-blind controlled trial. *J. Int. Med. Res.* 2018; 46, 1839-1845.
20. An X, Li C, Sahebally Z, et al. Pretreatment with Oxycodone Simultaneously Reduces Etomidate-Induced Myoclonus and Rocuronium-Induced Withdrawal Movements During Rapid-Sequence Induction. *Med. Sci. Monit.* 2017; 23, 4989-4994.
21. Cho SY, Jeon WJ, Nam YM, et al. The optimal dosage of alfentanil pretreatment for prevention of myoclonus after injection of etomidate. *Korean J. Anesthesiol.* 2008; 55, 320-325.
22. Singh K, Ruchi G, Singh A, et al. Efficacy of lignocaine versus midazolam in controlling etomidate-induced myoclonus: a randomized placebo-controlled study. *Ain-Shams Journal of Anaesthesiology* 2014; 7, 460.
23. Gupta P, Gupta M. Comparison of different doses of intravenous lignocaine on etomidate-induced myoclonus: A prospective randomised and placebo-controlled study. *Indian J. Anaesth.* 2018; 62, 121-126.
24. Gultop F, Akkaya T, Bedirli N, et al. Lidocaine pretreatment reduces the frequency and severity of myoclonus induced by etomidate. *J. Anesth.* 2010; 24, 300-302.
25. He L, Ding Y, Chen H, et al. Butorphanol pre-treatment prevents myoclonus induced by etomidate: a randomised, double-blind, controlled clinical trial. *Swiss Med. Wkly.* 2014; 144, w14042.
26. Zhang J, Liu L, Lv G. Comparison of the effects of intravenous pre-treatment of Butorphanol and Dezocine on prevention of Etomidate-induced myoclonus. *Tianjin Med J* 2015; 43, 1450-1453.
27. Ren J, Lan P, Yuan R. Effect of butorphanol pretreatment on myoclonus induced by etomidate during general anesthesia. *Shandong Med J* 2013; 53, 3.
28. Zhang J, Liu L, Liu H, et al. Comparison of butorphanol or midazolam alone and combination of the two drugs in preventing etomidate-induced myoclonus during anesthesia induction. *Chin J Anesthesiol* 2015; 35, 1325-1327.
29. Wu GN, Xu HJ, Liu FF, et al. Low-Dose Ketamine Pretreatment Reduces the Incidence and Severity of Myoclonus Induced by Etomidate: A Randomized, Double-Blinded, Controlled Clinical Trial. *Medicine* 2016; 95, e2701.
30. Guler A, Satilmis T, Akinci SB, et al. Magnesium sulfate pretreatment reduces myoclonus after etomidate. *Anesth. Analg.* 2005; 101, 705-709.
31. Un B, Ceyhan D, Yelken B. Prevention of etomidate-related myoclonus in anesthetic induction by pretreatment with magnesium. *Journal of Research in Medical Sciences the Official Journal of Isfahan University of Medical Sciences* 2011; 16, 1490-1494.
32. Sedighinejad A, Naderi Nabi B, Haghighi M, et al. Comparison of the Effects of Low-Dose Midazolam, Magnesium Sulfate, Remifentanil and Low-Dose Etomidate on Prevention of Etomidate-Induced Myoclonus in Orthopedic Surgeries. *Anesthesiology and pain medicine* 2016; 6, e35333.
33. Aissaoui Y, Belyamani L, Wali AE, et al. [Prevention of myoclonus after etomidate using a priming dose]. *Annales Franaises Danesthèsie Et De Rèanimation* 2006; 25, 1041-1045.
34. Ren Y, Shi W, Chen C, et al. Effect of preinjection of Nalbuphine on Etomidate - induced myoclonus during induction of general anesthesia. *CHINA MEDICAL HERALD* 2019; 16, 4.

35. Gupta M, Gupta P. Nalbuphine pretreatment for prevention of etomidate induced myoclonus: A prospective, randomized and double-blind study. *J. Anaesthesiol. Clin. Pharmacol.* 2018; 34, 200-204.
36. Choi JM, Choi IC, Yong BJ, et al. Pretreatment of rocuronium reduces the frequency and severity of etomidate-induced myoclonus. *J. Clin. Anesth.* 2008; 20, 601-604.
37. Mullick P, Talwar V, Aggarwal S, et al. Comparison of priming versus slow injection for reducing etomidate-induced myoclonus: a randomized controlled study. *Korean J. Anesthesiol.* 2018; 71, 305-310.
38. Fu X, Liu Z, Liu Y, et al. Tramadol Pretreatment Reduces Myoclonus Induced by Etomidate. *Med J of Wuhan University* 2018; 39, 4.
39. Yu H, Fang L, Du R, et al. Efficacy of Pretreatment of Vecuronium Combined with Dilution of Etomidate on Etomidate-induced Myoclonus. *WEST CHINA MED J* 2009; 24, 4.
40. Rautela RS, Gulabani M, Kumar P, Salhotra R, Mohta M, Verma K. Comparative assessment of dexmedetomidine and butorphanol for attenuation of etomidate-induced myoclonus: A double-blind, randomised controlled study. Indian J Anaesth. 2023;67:815-20.
41. Alipour M, Abdi N, Zaj P, Mashhadi L. Efficacy of Granisetron versus Sufentanil on Reducing Myoclonic Movements Following Etomidate: Double-blind, randomised clinical trial. Sultan Qaboos Univ Med J. 2023;23:380-6.
42. Shan G, Lu H, Dai F, Liu Y, Yin D, Cao H. Low-dose nalmefene pretreatment reduces etomidate-induced myoclonus: A randomized, double-blind controlled trial. Medicine. 2023;102:e35138.
43.Peng Y, Xisheng S, Fuhai J, Ju Q. Infuence of Low Dose Propofol Prejection on the Incidence of Etomidate Induced Myoclonus During General Anesthesia in Elderly Patients. Chinese Journal of Hemorheology. 2022;32.
44. Siddiqui A, Dhamnani R, Sinha J, Mandloi P. Comparison between Fentanyl and Nalbuphine Pretreatment in Prevention of Etomidate Induced Myoclonus International Journal of Pharmaceutical and Clinical Research. 2023;15:1137-43.
45. RAJKUMAR G, SHAMMY N, THOKCHOM RS, SINGH TH, DHAYANITHY M, DEVI KR, et al. Dexmedetomidine versus Lignocaine in the Prevention of Etomidate-induced Myoclonus-A Randomised Double-blinded Study. Journal of Clinical & Diagnostic Research. 2023;1
46. Siddiqui A, Agrawal A, Shaikh M, Siddiqui F. Efficacy of nalbuphine pre-treatment in attenuation of etomidate induced myoclonus: A placebo controlled trial from Malwa region. J Cardiovasc Dis Res. 2023;14:1385-9.
47. Rani A, Narwa Y, Arora G. Evaluating the Comparative Efficacy of Various Dosages of Midazolam in Preventing Etomidate-Induced Myoclonus: A Hospital based Study 2023;15:834-41.
48. Agrawal M, Tambey R, Patil V. A prospective randomized controlled study to compare effectiveness of dexmedetomidine and lignocaine pre-treatment for prevention of etomidate induced myoclonus J Cardiovasc Dis Res. 2023;14:321-31.

Table S4 Results of the risk of bias of included studies

Unique ID	Randomization process	Deviations from intended interventions	Missing outcome data	Measurement of the outcome	Selection of the reported result	Overall Bias
Aissaoui 2006	Low	Low	Low	Low	Low	Low
Alipour 2016	Low	Low	Low	Low	Low	Low
An 2017	Low	Low	Low	Low	Some concerns	Some concerns
Chen 2009	Low	Low	Low	Low	Low	Low
Chen 2016	Some concerns	Low	Low	High	Low	High
Chen 2017	Some concerns	Low	Low	Low	Low	Some concerns
Cho 2008	Low	Low	Low	Low	Low	Low
Choi 2008	Low	Low	Low	Low	Low	Low
Fu 2018	Some concerns	Low	Low	Low	Low	Some concerns
Guler 2005	Low	Low	Low	Low	Low	Low
Gultop 2010	Low	Low	Low	Low	Low	Low
Gupta 2018a	Low	Low	Low	Low	Low	Low
Gupta 2018[b]	Low	Low	Low	Low	Some concerns	Some concerns
He 2014	Low	Low	Low	Low	Low	Low
Hwang 2008	Low	Low	Low	Low	Low	Low
Ko 2013	Low	Some concerns	Low	Low	Low	Some concerns
Kwon 2002	Some concerns	Low	Low	High	Low	High
Lee 2009	Low	Low	Low	Low	Low	Low
Luan 2015	Some concerns	Low	Low	Low	Low	Some concerns
Lv 2018	Some concerns	Low	Low	Low	Low	Some concerns
Ma 2015	Low	Low	Low	Low	Low	Low
Miao 2019	Low	Low	Low	Low	Low	Low
Mizrark 2010	Low	Low	Low	Low	Low	Low
Mullick 2018	Low	Some concerns	Low	Low	Low	Some concerns
Patel 2015	Some concerns	Low	Low	High	Low	High
Ren 2013	Some concerns	Low	Low	High	Low	High
Ren 2019	Low	Low	Low	Some concerns	Low	Some concerns
Sedighinejad 2016	Low	Low	Low	Low	Low	Low

Singh 2014	Some concerns	Low	Low	High	Low	High
Un 2011	Low	Low	Low	Some concerns	Low	Some concerns
Wang 2015	Some concerns	Low	Low	Low	Low	Some concerns
Wang 2018[a]	Some concerns	Low	Low	High	Low	High
Wang 2018[b]	Low	Low	Low	Low	Low	Low
Wu 2016	Low	Low	Low	Low	Low	Low
Xie 2016	Some concerns	Low	Low	High	Low	High
Yu 2009	Low	Low	Low	Low	Low	Low
Zhang 2015[a]	Low	Low	Low	Low	Low	Low
Zhang 2015[b]	Some concerns	Low	Low	High	Low	High
Zhang 2021	Some concerns	Low	Low	High	Low	High
Agrawal 2023	Some concerns	Low	Low	Low	Low	Some concerns
Rautela 2023	Low	Low	Low	Low	Low	Low
Siddiqui 2023[a]	Some concerns	Low	Low	High	Low	High
Siddiqui 2023[b]	Some concerns	Low	Low	High	Low	High
Rajkumar 2023	Low	Low	Low	Low	Low	Low
Alipour 2023	Low	Low	Low	Low	Low	Low
Rani 2023	Some concerns	Low	Low	Low	Low	Some concerns
Peng 2022	Some concerns	Low	Low	High	Low	High
Shan 2023	Low	Low	Low	Low	Low	Low

Table S5A Results of traditional pairwise meta-analysis of overall risk of EIM (primary outcome)

			Pairwise OR (95% Cl)		Heterogeneity	
T1	T2	Studies	Fixed effects	Random effects	I^2	P-value
Dexmedetomidine	NS	7	0.15 (0.10, 0.22)	0.16 (0.09, 0.29)	48.10%	0.072
Remifentanil	NS	6	0.03 (0.02, 0.07)	0.04 (0.02, 0.07)	0.00%	0.918
Fentanyl	NS	6	0.15 (0.10, 0.23)	0.15 (0.10, 0.23)	0.00%	0.938
Midazolam	NS	6	0.14 (0.09, 0.23)	0.14 (0.07, 0.28)	49.60%	0.078
Lidocaine	NS	4	0.18 (0.11, 0.30)	0.15 (0.05, 0.50)	77.30%	0.004
Butorphanol	NS	4	0.05 (0.03, 0.08)	0.05 (0.03, 0.08)	0.00%	0.811
Oxycodone	NS	3	0.05 (0.03, 0.09)	0.02 (0.00, 0.41)	85.70%	0.001
Sufentanil	NS	4	0.06 (0.03, 0.11)	0.05 (0.01, 0.17)	65.40%	0.034
Ketamine	NS	2	0.25 (0.13, 0.48)	0.28 (0.04, 2.23)	88.80%	0.003
Magnesium sulfate	NS	2	0.22 (0.11, 0.43)	0.21 (0.10, 0.45)	8.10%	0.297
Low-dose etomidate	NS	2	0.19 (0.09, 0.39)	0.14 (0.03, 0.72)	72.20%	0.058
Nalbuphine	NS	3	0.08 (0.04, 0.16)	0.08 (0.04, 0.16)	0.00%	0.528
Thiopental	NS	1	0.34 (0.12, 0.96)	NA	NA	NA
Vecuronium	NS	1	0.18 (0.05, 0.71)	NA	NA	NA
Alfentanil	NS	1	0.02 (0.00, 0.11)	NA	NA	NA
Rocuronium	NS	1	0.20 (0.09, 0.44)	NA	NA	NA
Tramadol	NS	1	0.21 (0.05, 1.01)	NA	NA	NA
Low-dose propofol	NS	1	0.14 (0.06, 0.36)	NA	NA	NA
Nalmefene	NS	1	0.03 (0.01, 0.11)	NA	NA	NA
Granisetron	NS	1	0.01 (0.00, 0.04)	NA	NA	NA
Dexmedetomidine	Lidocaine	3	1.14 (0.64, 2.03)	1.12 (0.42, 2.98)	52.10%	0.124
Remifentanil	Fentanyl	3	0.20 (0.04, 0.97)	0.25 (0.06, 1.09)	0.00%	0.737
Oxycodone	Fentanyl	2	0.02 (0.00, 0.14)	0.02 (0.00, 0.14)	0.00%	0.944
Remifentanil	Midazolam	2	0.62 (0.33, 1.14)	0.62 (0.33, 1.15)	0.00%	0.394
Remifentanil	Magnesium sulfate	1	0.22 (0.10, 0.51)	NA	NA	NA
Remifentanil	Low-dose etomidate	1	1.87 (0.96, 3.64)	NA	NA	NA
Sufentanil	Remifentanil	1	5.74 (0.25, 130.37)	NA	NA	NA
Sufentanil	Fentanyl	1	0.62 (0.09, 4.34)	NA	NA	NA
Sufentanil	Midazolam	1	0.07 (0.02, 0.29)	NA	NA	NA
Sufentanil	Granisetron	1	4.20 (0.80, 22.06)	NA	NA	NA
Thiopental	Dexmedetomidine	1	1.16 (0.40, 3.35)	NA	NA	NA
Butorphanol	Dexmedetomidine	1	3.05 (1.05, 8.84)	NA	NA	NA
Magnesium sulfate	Ketamine	1	0.15 (0.05, 0.44)	NA	NA	NA
Magnesium sulfate	Low-dose etomidate	1	8.34 (3.68, 18.89)	NA	NA	NA
Magnesium sulfate	Midazolam	1	2.39 (1.03, 5.57)	NA	NA	NA
Low-dose etomidate	Midazolam	1	0.29 (0.14, 0.58)	NA	NA	NA
Lidocaine	Midazolam	1	2.02 (0.62, 6.56)	NA	NA	NA
Dexmedetomidine	Midazolam	1	0.75 (0.26, 2.15)	NA	NA	NA
Butorphanol	Midazolam	1	0.67 (0.19, 2.33)	NA	NA	NA
Nalmefene	Fentanyl	1	0.18 (0.06, 0.60)	NA	NA	NA
Nalbuphine	Fentanyl	1	0.29 (0.09, 0.90)	NA	NA	NA

Table S5B Results of traditional pairwise meta-analysis of risk of EIM at a mild intensity level (secondary outcome)

			Pairwise OR (95% Cl)		Heterogeneity	
T1	T2	Studies	Fixed effects	Random effects	I^2	P-value
Dexmedetomidine	NS	7	0.49 (0.31, 0.77)	0.53 (0.33, 0.84)	0.00%	0.441
Remifentanil	NS	6	0.25 (0.13, 0.48)	0.27 (0.12, 0.61)	21.10%	0.275
Fentanyl	NS	6	0.44 (0.25, 0.75)	0.45 (0.25, 0.78)	0.00%	0.620
Midazolam	NS	6	1.16 (0.73, 1.85)	1.09 (0.62, 1.91)	23.00%	0.261
Lidocaine	NS	4	0.60 (0.33, 1.10)	0.60 (0.23, 1.55)	42.70%	0.155
Butorphanol	NS	4	0.42 (0.22, 0.79)	0.43 (0.22, 0.81)	0.00%	0.655
Sufentanil	NS	4	0.30 (0.16, 0.56)	0.24 (0.06, 0.99)	68.20%	0.024
Oxycodone	NS	3	0.17 (0.08, 0.38)	0.15 (0.03, 0.69)	40.30%	0.187
Nalbuphine	NS	3	0.40 (0.19, 0.86)	0.40 (0.11, 1.43)	57.40%	0.096
Ketamine	NS	2	1.37 (0.65, 2.91)	1.35 (0.56, 3.27)	24.60%	0.250
Low-dose etomidate	NS	2	1.39 (0.63, 3.07)	1.39 (0.62, 3.11)	0.00%	0.369
Magnesium sulfate	NS	1	0.13 (0.01, 1.19)	NA	NA	NA
Thiopental	NS	1	0.82 (0.24, 2.81)	NA	NA	NA
Vecuronium	NS	1	1.00 (0.18, 5.67)	NA	NA	NA
Alfentanil	NS	1	0.19 (0.03, 1.26)	NA	NA	NA
Rocuronium	NS	1	0.55 (0.23, 1.28)	NA	NA	NA
Tramadol	NS	1	6.00 (1.29, 27.91)	NA	NA	NA
Granisetron	NS	1	0.04 (0.01, 0.20)	NA	NA	NA
Low-dose propofol	NS	1	0.44 (0.14, 1.40)	NA	NA	NA
Nalmefene	NS	1	0.40 (0.11, 1.38)	NA	NA	NA
Dexmedetomidine	Lidocaine	3	3.06 (1.40, 6.69)	2.89 (1.15, 7.29)	15%	0.308
Oxycodone	Fentanyl	2	0.10 (0.01, 0.83)	0.10 (0.01, 0.83)	0.00%	0.999
Remifentanil	Fentanyl	3	0.31 (0.06, 1.61)	0.37 (0.08, 1.64)	0.00%	0.962
Remifentanil	Midazolam	2	2.03 (0.82, 5.02)	1.70 (0.22, 12.98)	75.50%	0.044
Remifentanil	Magnesium sulfate	1	3.41 (1.04, 11.14)	NA	NA	NA
Remifentanil	Low-dose etomidate	1	1.00 (0.42, 2.41)	NA	NA	NA
Midazolam	Magnesium sulfate	1	0.74 (0.16, 3.43)	NA	NA	NA
Midazolam	Low-dose etomidate	1	0.22 (0.06, 0.81)	NA	NA	NA
Midazolam	Dexmedetomidine	1	1.41 (0.45, 4.45)	NA	NA	NA
Midazolam	Lidocaine	1	0.60 (0.15, 2.47)	NA	NA	NA
Midazolam	Butorphanol	1	1.29 (0.32, 5.19)	NA	NA	NA
Sufentanil	Remifentanil	1	0.31 (0.01, 8.28)	NA	NA	NA
Sufentanil	Fentanyl	1	0.31 (0.01, 8.28)	NA	NA	NA
Sufentanil	Midazolam	1	5.43 (0.25, 118.96)	NA	NA	NA
Thiopental	Dexmedetomidine	1	1.25 (0.34, 4.64)	NA	NA	NA
Magnesium sulfate	Ketamine	1	0.06 (0.01, 0.50)	NA	NA	NA
magnesium sulfate	low-dose etomidate	1	0.29 (0.09, 0.96)	NA	NA	NA
Butorphanol	Dexmedetomidine	1	0.46 (0.08, 2.75)	NA	NA	NA
Fentanyl	Nalbuphine	1	4.26 (0.81, 22.53)	NA	NA	NA
Fentanyl	Nalmefene	1	2.19 (0.61, 7.81)	NA	NA	NA
Granisetron	Sufentanil	1	0.36 (0.06, 2.01)	NA	NA	NA

Table S5C Results of traditional pairwise meta-analysis of the risk of EIM at moderate-to-severe intensity level (secondary outcome)

			Pairwise OR (95% Cl)		Heterogeneity	
T1	T2	Studies	Fixed effects	Random effects	I^2	P-value
Dexmedetomidine	NS	7	0.11 (0.06, 0.19)	0.14 (0.08, 0.26)	20.30%	0.215
Remifentanil	NS	6	0.01 (0.00, 0.05)	0.03 (0.01, 0.09)	27.60%	0.228
Fentanyl	NS	6	0.18 (0.12, 0.29)	0.19 (0.12, 0.30)	0.00%	0.738
Midazolam	NS	6	0.14 (0.09, 0.22)	0.13 (0.05, 0.30)	60.70%	0.026
Lidocaine	NS	4	0.23 (0.15, 0.37)	0.23 (0.09, 0.57)	59.70%	0.059
Butorphanol	NS	4	0.04 (0.02, 0.08)	0.04 (0.02, 0.08)	0.00%	0.632
Sufentanil	NS	4	0.14 (0.08, 0.26)	0.12 (0.03, 0.41)	61.40%	0.051
Oxycodone	NS	3	0.05 (0.02, 0.11)	0.03 (0.00, 0.49)	79.80%	0.007
Nalbuphine	NS	3	0.07 (0.03, 0.15)	0.08 (0.03, 0.17)	0.00%	0.614
Ketamine	NS	2	0.17 (0.09, 0.35)	0.18 (0.03, 0.99)	82.00%	0.018
Low-dose etomidate	NS	2	0.20 (0.11, 0.38)	0.16 (0.05, 0.56)	59.20%	0.118
Magnesium sulfate	NS	1	0.27 (0.08, 0.95)	NA	NA	NA
Thiopental	NS	1	0.30 (0.09, 1.00)	NA	NA	NA
Vecuronium	NS	1	0.14 (0.03, 0.65)	NA	NA	NA
Alfentanil	NS	1	0.05 (0.01, 0.18)	NA	NA	NA
Rocuronium	NS	1	0.09 (0.02, 0.41)	NA	NA	NA
Tramadol	NS	1	0.13 (0.04, 0.41)	NA	NA	NA
Granisetron	NS	1	0.04 (0.00, 0.69)	NA	NA	NA
Low-dose propofol	NS	1	0.16 (0.05, 0.47)	NA	NA	NA
Nalmefene	NS	1	0.01 (0.00, 0.14)	NA	NA	NA
Lidocaine	Dexmedetomidine	3	3.01 (1.27, 7.12)	2.80 (1.19, 6.58)	0.00%	0.513
Oxycodone	Fentanyl	2	0.03 (0.00, 0.19)	0.03 (0.00, 0.19)	0.00%	0.932
Remifentanil	Fentanyl	2	0.23 (0.02, 2.22)	0.23 (0.02, 2.22)	0.00%	0.789
Remifentanil	Midazolam	2	0.40 (0.21, 0.77)	0.89 (0.06, 12.51)	67.40%	0.080
Remifentanil	Magnesium sulfate	1	0.17 (0.08, 0.36)	NA	NA	NA
Remifentanil	Low-dose etomidate	1	2.03 (1.00, 4.15)	NA	NA	NA
Midazolam	Magnesium sulfate	1	0.51 (0.24, 1.10)	NA	NA	NA
Midazolam	Low-dose etomidate	1	6.14 (2.96, 12.75)	NA	NA	NA
Midazolam	Dexmedetomidine	1	1.00 (0.19, 5.40)	NA	NA	NA
Midazolam	Lidocaine	1	0.55 (0.12, 2.58)	NA	NA	NA
Midazolam	Butorphanol	1	2.05 (0.18, 23.59)	NA	NA	NA
Sufentanil	Remifentanil	1	5.74 (0.25, 130.37)	NA	NA	NA
Sufentanil	Fentanyl	1	1.00 (0.12, 8.21)	NA	NA	NA
Sufentanil	Midazolam	1	0.05 (0.01, 0.20)	NA	NA	NA
Sufentanil	Granisetron	1	5.33 (0.25, 115.50)	NA	NA	NA
Thiopental	Dexmedetomidine	1	1.00 (0.26, 3.89)	NA	NA	NA
Magnesium sulfate	Ketamine	1	0.64 (0.20, 2.05)	NA	NA	NA
Magnesium sulfate	Low-dose etomidate	1	11.99 (5.43, 26.47)	NA	NA	NA
Dexmedetomidine	Butorphanol	1	0.25 (0.08, 0.73)	NA	NA	NA
Nalbuphine	Fentanyl	1	0.51 (0.13, 1.95)	NA	NA	NA
Nalmefene	Fentanyl	1	0.05 (0.00, 0.88)	NA	NA	NA

Table S5D Results of traditional pairwise meta-analysis of risk of adverse events (secondary outcome)

			Pairwise OR (95% Cl)		Heterogeneity	
T1	T2	Studies	Fixed effects	Random effects	I^2	P-value
Dexmedetomidine	NS	6	0.69 (0.42, 1.12)	0.86 (0.17, 4.36)	80.90%	0.002
Oxycodone	NS	3	6.64 (1.17, 37.65)	6.59 (1.16, 37.45)	0.00%	0.983
Sufentanil	NS	2	1.00 (0.38, 2.64)	1.14 (0.24, 5.33)	27.40%	0.241
Fentanyl	NS	3	1.65 (0.78, 3.49)	1.51 (0.72, 3.18)	0.00%	0.478
Midazolam	NS	2	0.29 (0.13, 0.64)	0.28 (0.05, 1.38)	72.00%	0.059
Lidocaine	NS	2	0.34 (0.14, 0.84)	0.35 (0.14, 0.88)	0.00%	0.480
Ketamine	NS	2	1.95 (0.66, 5.80)	1.97 (0.66, 5.82)	0.00%	0.717
Nalbuphine	NS	3	0.89 (0.46, 1.72)	0.89 (0.46, 1.73)	0.00%	0.602
Butorphanol	NS	1	12.11 (0.65, 224.67)	NA	NA	NA
Remifentanil	NS	1	8.06 (0.40, 163.21)	NA	NA	NA
Low-dose etomidate	NS	1	0.34 (0.10, 1.13)	NA	NA	NA
Magnesium sulfate	NS	1	0.53 (0.15, 1.93)	NA	NA	NA
Thiopental	NS	1	0.44 (0.16, 1.25)	NA	NA	NA
Vecuronium	NS	1	0.63 (0.09, 4.24)	NA	NA	NA
Alfentanil	NS	1	8.62 (0.48, 154.44)	NA	NA	NA
Rocuronium	NS	1	0.44 (0.12, 1.57)	NA	NA	NA
Tramadol	NS	1	35.65 (2.08, 611.65)	NA	NA	NA
Nalmefene	NS	1	0.14 (0.05, 0.37)	NA	NA	NA
Low-dose propofol	NS	1	1.00 (0.06, 16.50)	NA	NA	NA
Lidocaine	Dexmedetomidine	2	0.88 (0.32, 2.40)	0.87 (0.31, 2.42)	0.00%	0.445
Oxycodone	Fentanyl	2	1.26 (0.33, 4.83)	1.26 (0.33, 4.85)	0.00%	0.760
Remifentanil	Midazolam	1	8.06 (0.40, 163.21)	NA	NA	NA
Midazolam	Dexmedetomidine	1	1.53 (0.54, 4.36)	NA	NA	NA
Midazolam	Lidocaine	1	1.38 (0.45, 4.20)	NA	NA	NA
Thiopental	Dexmedetomidine	1	0.87 (0.32, 2.42)	NA	NA	NA
Magnesium sulfate	Ketamine	1	0.24 (0.07, 0.82)	NA	NA	NA
Nalmefene	Fentanyl	1	0.11 (0.04, 0.29)	NA	NA	NA
Nalbuphine	Fentanyl	1	1.00 (0.34, 2.93)	NA	NA	NA

Table S5E The duration of EIM (secondary outcome)

					Heterogeneity	
**T1**	**T2**	**Studies**	**Patients**	**MD (95% Cl)**	**I^2**	**P-value**
Remifentanil	NS	1	60	57.80 (-81.18, -34.42)	NA	NA
Midazolam	NS	2	130	-8.58 (-33.88, 16.72)	71.60%	0.061
Lidocaine	NS	1	50	1.74 (-3.31, 6.79)	NA	NA
Alfentanil	NS	1	38	-37.20 (-69.94, -4.46)	NA	NA
Sufentanil	Midazolam	1	50	-63.20 (-82.64, -43.76)	NA	NA
Midazolam	Lidocaine	1	50	0.41 (-4.85, 5.67)	NA	NA

Abbreviation: NS: normal saline; OR: odds ratio; MD: mean difference; Cl: confidence interval; EIM: etomidate-induced myoclonus; NA: not applicable;

Table S6A Results of Bayesian network meta-analysis of the risk of EIM at a mild intensity level (secondary outcome)

NS	**0.10 (0.02, 0.37)**	0.62 (0.27, 1.44)	0.60 (0.29, 1.20)	**0.36 (0.15, 0.80)**	**0.30 (0.10, 0.88)**	0.60 (0.30, 1.18)	0.79 (0.12, 4.95)	**0.38 (0.16, 0.92)**	**0.38 (0.15, 0.99)**	1.53 (0.39, 5.90)	**0.18 (0.04, 0.75)**	0.98 (0.32, 2.97)	**0.29 (0.09, 0.91)**	0.54 (0.08, 3.39)	0.18 (0.01, 2.22)	7.07 (0.80, 93.26)	0.99 (0.08, 12.50)	**0.05 (0.00, 0.42)**	0.32 (0.05, 2.03)	0.40 (0.05, 3.12)
	Oxycodone	6.09 (1.46, 34.91)	5.89 (1.38, 32.11)	3.52 (0.78, 20.39)	2.95 (0.54, 19.75)	5.91 (1.40, 32.37)	7.84 (0.83, 89.28)	3.78 (0.81, 22.28)	3.76 (0.77, 23.04)	15.05 (2.40, 117.70)	1.73 (0.23, 14.16)	9.58 (1.78, 66.01)	2.88 (0.54, 19.93)	5.34 (0.60, 62.12)	1.78 (0.09, 35.24)	70.77 (5.76, 1492.70)	9.87 (0.61, 188.17)	0.51 (0.03, 7.12)	3.09 (0.32, 36.17)	4.01 (0.37, 53.26)
		Fentanyl	0.97 (0.31, 2.73)	0.58 (0.18, 1.69)	0.48 (0.12, 1.86)	0.97 (0.33, 2.80)	1.26 (0.17, 9.35)	0.62 (0.18, 2.05)	0.62 (0.17, 2.15)	2.47 (0.49, 11.70)	0.28 (0.05, 1.47)	1.57 (0.40, 6.11)	0.47 (0.13, 1.66)	0.88 (0.11, 6.46)	0.29 (0.02, 4.24)	11.44 (1.06, 165.95)	1.60 (0.12, 23.02)	0.08 (0.01, 0.79)	0.51 (0.07, 3.30)	0.65 (0.07, 5.83)
			Midazolam	0.60 (0.22, 1.63)	0.50 (0.14, 1.78)	1.01 (0.40, 2.61)	1.31 (0.18, 9.48)	0.64 (0.22, 1.85)	0.64 (0.21, 2.00)	2.54 (0.57, 11.87)	0.29 (0.06, 1.38)	1.62 (0.48, 5.80)	0.49 (0.13, 1.90)	0.91 (0.12, 6.70)	0.30 (0.02, 4.25)	11.84 (1.16, 174.53)	1.65 (0.13, 22.85)	0.09 (0.01, 0.80)	0.53 (0.07, 3.94)	0.67 (0.08, 6.22)
				Remifentanil	0.83 (0.22, 3.35)	1.69 (0.59, 5.12)	2.19 (0.30, 16.91)	1.07 (0.32, 3.66)	1.06 (0.31, 3.91)	4.27 (0.90, 21.30)	0.49 (0.10, 2.39)	2.71 (0.78, 9.92)	0.82 (0.20, 3.40)	1.51 (0.21, 12.07)	0.50 (0.03, 7.33)	20.04 (1.89, 289.13)	2.76 (0.21, 41.09)	0.15 (0.01, 1.45)	0.88 (0.11, 6.86)	1.14 (0.13, 10.64)
					Sufentanil	2.02 (0.57, 7.34)	2.66 (0.30, 22.24)	1.29 (0.31, 5.25)	1.27 (0.30, 5.45)	5.09 (0.90, 28.88)	0.59 (0.08, 3.69)	3.28 (0.68, 15.24)	0.99 (0.20, 4.69)	1.83 (0.21, 15.38)	0.59 (0.03, 9.22)	23.99 (2.07, 391.39)	3.33 (0.22, 53.65)	0.18 (0.01, 1.48)	1.06 (0.12, 9.43)	1.36 (0.14, 13.39)
						Dexmedetomidine	1.30 (0.20, 8.39)	0.64 (0.24, 1.61)	0.63 (0.21, 1.89)	2.51 (0.57, 11.58)	0.29 (0.05, 1.44)	1.61 (0.44, 5.91)	0.49 (0.13, 1.83)	0.90 (0.13, 6.55)	0.29 (0.02, 4.23)	11.71 (1.17, 169.32)	1.64 (0.12, 22.43)	0.09 (0.01, 0.79)	0.52 (0.07, 3.91)	0.67 (0.08, 5.96)
							Thiopental	0.49 (0.07, 3.64)	0.49 (0.06, 3.87)	1.94 (0.20, 18.73)	0.22 (0.02, 2.32)	1.24 (0.15, 10.33)	0.38 (0.04, 3.33)	0.69 (0.05, 9.76)	0.22 (0.01, 5.37)	9.11 (0.50, 218.99)	1.25 (0.06, 30.11)	0.07 (0.00, 1.14)	0.39 (0.03, 5.68)	0.52 (0.03, 8.46)
								Lidocaine	0.99 (0.28, 3.64)	4.00 (0.80, 20.46)	0.45 (0.08, 2.51)	2.53 (0.61, 10.62)	0.76 (0.18, 3.28)	1.40 (0.19, 11.17)	0.46 (0.03, 6.98)	18.43 (1.75, 285.05)	2.58 (0.19, 37.23)	0.13 (0.01, 1.37)	0.82 (0.10, 6.52)	1.06 (0.12, 10.00)
									Butorphanol	4.02 (0.75, 21.18)	0.46 (0.07, 2.65)	2.55 (0.58, 11.00)	0.77 (0.17, 3.40)	1.43 (0.18, 11.55)	0.47 (0.03, 6.96)	18.52 (1.72, 286.85)	2.61 (0.18, 38.45)	0.14 (0.01, 1.34)	0.83 (0.10, 6.93)	1.07 (0.11, 10.29)
										Ketamine	0.12 (0.02, 0.67)	0.64 (0.12, 3.47)	0.19 (0.03, 1.12)	0.35 (0.04, 3.47)	0.12 (0.01, 2.03)	4.70 (0.35, 80.58)	0.65 (0.04, 10.92)	0.03 (0.00, 0.44)	0.21 (0.02, 2.12)	0.27 (0.02, 3.17)
											Magnesium sulfate	5.55 (1.12, 31.51)	1.67 (0.26, 12.02)	3.06 (0.30, 35.82)	1.02 (0.05, 20.86)	40.91 (2.91, 843.09)	5.68 (0.33, 115.21)	0.29 (0.02, 4.28)	1.80 (0.16, 20.74)	2.35 (0.18, 31.69)
												Low-dose etomidate	0.30 (0.06, 1.46)	0.55 (0.06, 4.83)	0.18 (0.01, 2.99)	7.28 (0.63, 118.49)	1.00 (0.07, 16.23)	0.05 (0.00, 0.60)	0.32 (0.04, 2.91)	0.42 (0.04, 4.40)
													Nalbuphine	1.83 (0.21, 16.54)	0.61 (0.03, 9.82)	24.07 (2.03, 401.65)	3.36 (0.22, 57.40)	0.17 (0.01, 1.96)	1.07 (0.12, 9.44)	1.37 (0.13, 14.74)
														Rocuronium	0.32 (0.01, 7.74)	13.32 (0.73, 305.50)	1.82 (0.09, 41.21)	0.10 (0.00, 1.56)	0.58 (0.04, 8.33)	0.76 (0.05, 11.89)
															Alfentanil	41.20 (1.32, 1673.45)	5.55 (0.16, 227.22)	0.29 (0.01, 8.98)	1.81 (0.07, 49.18)	2.31 (0.09, 68.20)
																Tramadol	0.14 (0.00, 4.01)	0.01 (0.00, 0.16)	0.04 (0.00, 0.82)	0.06 (0.00, 1.21)
																	Vecuronium	0.05 (0.00, 1.37)	0.31 (0.01, 7.14)	0.41 (0.02, 10.13)
																		Granisetron	6.06 (0.36,134.32)	7.86 (0.40, 198.93)
																			Nalmefene	1.30 (0.08, 21.09)
																				Low-dose propofol

Effect sizes represent summary odds ratios (OR) and 95% confidence intervals. OR<1 indicate that the intervention specified in the column got more prophylactic effect than that specified in the row, which values are in bold. Abbreviation: NS: normal saline; EIM: etomidate-induced myoclonus.

Table S6B Results of Bayesian network meta-analysis of the risk of EIM at moderate-to-severe intensity level (secondary outcome)

NS	**0.01 (0.00, 0.08)**	**0.16 (0.05, 0.47)**	**0.12 (0.04, 0.31)**	**0.02 (0.00, 0.07)**	**0.06 (0.01, 0.21)**	**0.07 (0.02, 0.18)**	0.14 (0.01, 1.73)	**0.19 (0.06, 0.61)**	**0.06 (0.02, 0.20)**	0.18 (0.03, 1.13)	0.21 (0.03, 1.18)	**0.05 (0.01, 0.22)**	**0.05 (0.01, 0.25)**	0.07 (0.00, 1.33)	**0.04 (0.00, 0.75)**	0.11 (0.01, 1.83)	0.12 (0.01, 2.37)	**0.00 (0.00, 0.01)**	**0.00 (0.00, 0.01)**	0.14 (0.01, 2.26)
	oxycodone	11.59 (1.80,108.14)	8.86 (1.23, 87.08)	1.33 (0.14, 14.83)	4.25 (0.49, 50.08)	4.92 (0.67, 49.77)	10.68 (0.51, 286.15)	13.93 (1.75, 155.23)	4.13 (0.48, 47.73)	13.29 (1.08, 221.74)	15.45 (1.34, 233.49)	3.63 (0.37, 48.01)	3.99 (0.41, 51.14)	5.23 (0.15, 203.69)	3.26 (0.12, 117.67)	8.34 (0.31, 288.38)	9.23 (0.30, 365.19)	0.00 (0.00, 0.30)	0.00 (0.00, 0.10)	10.70 (0.42, 358.47)
		fentanyl	0.76 (0.17, 3.26)	0.11 (0.02, 0.60)	0.36 (0.07, 1.92)	0.42 (0.09, 1.89)	0.91 (0.06, 14.40)	1.19 (0.24, 6.09)	0.35 (0.06, 1.92)	1.14 (0.13, 9.91)	1.32 (0.16, 10.36)	0.31 (0.05, 1.99)	0.34 (0.06, 1.87)	0.45 (0.02, 10.54)	0.28 (0.01, 5.97)	0.71 (0.03, 14.63)	0.79 (0.03, 18.85)	0.00 (0.00, 0.02)	0.00 (0.00, 0.01)	0.91 (0.05, 18.04)
			midazolam	0.15 (0.03, 0.66)	0.48 (0.11, 2.23)	0.55 (0.15, 2.16)	1.19 (0.08, 17.65)	1.57 (0.38, 6.92)	0.47 (0.10, 2.23)	1.49 (0.19, 12.01)	1.73 (0.27, 11.29)	0.41 (0.08, 2.20)	0.45 (0.07, 2.81)	0.59 (0.02, 13.57)	0.37 (0.02, 7.71)	0.93 (0.05, 18.77)	1.04 (0.04, 23.97)	0.00 (0.00, 0.03)	0.00 (0.00, 0.01)	1.19 (0.06, 23.18)
				remifentanil	3.19 (0.53, 23.41)	3.70 (0.71, 23.11)	8.02 (0.47, 154.72)	10.50 (1.84, 74.34)	3.11 (0.50, 22.94)	9.99 (1.11, 108.11)	11.61 (1.65, 93.02)	2.73 (0.46, 19.05)	3.01 (0.39, 26.36)	3.93 (0.13, 114.41)	2.45 (0.11, 65.52)	6.27 (0.29, 160.11)	6.93 (0.27,205.00)	0.00 (0.00, 0.22)	0.00 (0.00, 0.07)	8.02 (0.38, 199.83)
					sufentanil	1.16 (0.22, 6.11)	2.51 (0.14, 42.37)	3.29 (0.57, 19.36)	0.97 (0.15, 6.14)	3.13 (0.32, 30.59)	3.63 (0.41, 31.45)	0.85 (0.12, 6.21)	0.94 (0.12, 7.09)	1.23 (0.04, 31.40)	0.77 (0.03, 17.94)	1.96 (0.09, 43.97)	2.17 (0.08, 56.01)	0.00 (0.00, 0.07)	0.00 (0.00, 0.02)	2.50 (0.12, 54.44)
						dexmedetomidine	2.15 (0.17, 26.94)	2.83 (0.78, 10.50)	0.84 (0.19, 3.71)	2.69 (0.33, 22.47)	3.12 (0.41, 23.34)	0.73 (0.12, 4.48)	0.81 (0.12, 5.09)	1.06 (0.04, 24.21)	0.66 (0.03, 13.74)	1.68 (0.09, 33.60)	1.86 (0.08, 43.03)	0.00 (0.00, 0.06)	0.00 (0.00, 0.02)	2.15 (0.11, 41.69)
							thiopental	1.32 (0.09, 20.63)	0.39 (0.02, 6.52)	1.25 (0.06, 29.37)	1.45 (0.07, 31.61)	0.34 (0.02, 6.53)	0.38 (0.02, 7.29)	0.49 (0.01, 24.32)	0.31 (0.01, 14.15)	0.78 (0.02, 35.07)	0.86 (0.02, 43.51)	0.00 (0.00, 0.03)	0.00 (0.00, 0.01)	1.00 (0.02, 43.41)
								lidocaine	0.30 (0.05, 1.59)	0.95 (0.11, 8.52)	1.10 (0.13, 8.81)	0.26 (0.04, 1.73)	0.29 (0.04, 1.96)	0.37 (0.01, 8.97)	0.23 (0.01, 5.11)	0.59 (0.03, 12.56)	0.66 (0.03, 15.89)	0.00 (0.00, 0.02)	0.00 (0.00, 0.01)	0.76 (0.04, 15.49)
									butorphanol	3.21 (0.34, 30.98)	3.74 (0.42, 32.22)	0.88 (0.12, 6.30)	0.97 (0.12, 7.17)	1.26 (0.04, 31.87)	0.78 (0.03, 18.37)	2.01 (0.09, 44.67)	2.22 (0.08, 57.08)	0.00 (0.00, 0.07)	0.00 (0.00, 0.02)	2.57 (0.12, 55.13)
										ketamine	1.16 (0.13, 10.28)	0.27 (0.03, 2.77)	0.30 (0.03, 3.31)	0.39 (0.01, 12.97)	0.25 (0.01, 7.38)	0.63 (0.02, 17.97)	0.69 (0.02, 22.94)	0.00 (0.00, 0.02)	0.00 (0.00, 0.01)	0.80 (0.03, 22.42)
											magnesium sulfate	0.24 (0.03, 1.86)	0.26 (0.02, 2.73)	0.34 (0.01, 10.92)	0.21 (0.01, 6.19)	0.54 (0.02, 15.07)	0.60 (0.02, 19.19)	0.00 (0.00, 0.02)	0.00 (0.00, 0.01)	0.69 (0.03, 18.74)
												low-dose etomidate	1.11 (0.12, 9.47)	1.43 (0.04, 40.08)	0.90 (0.04, 22.97)	2.29 (0.10, 54.83)	2.55 (0.09, 71.57)	0.00 (0.00, 0.05)	0.00 (0.00, 0.02)	2.94 (0.13, 70.31)
													nalbuphine	1.30 (0.04, 38.29)	0.81 (0.03, 21.50)	2.09 (0.08, 51.61)	2.30 (0.08, 67.99)	0.00 (0.00, 0.05)	0.00 (0.00, 0.02)	2.67 (0.11, 65.88)
														rocuronium	0.63 (0.01, 44.15)	1.60 (0.03, 105.74)	1.77 (0.03, 131.30)	0.00 (0.00, 0.04)	0.00 (0.00, 0.01)	2.05 (0.03, 133.88)
															alfentanil	2.56 (0.05, 138.34)	2.82 (0.04, 172.95)	0.00 (0.00, 0.06)	0.00 (0.00, 0.02)	3.27 (0.06, 173.82)
																tramadol	1.11 (0.02, 64.82)	0.00 (0.00, 0.02)	0.00 (0.00, 0.01)	1.28 (0.02, 66.91)
																	vecuronium	0.00 (0.00, 0.02)	0.00 (0.00, 0.01)	1.16 (0.02, 71.02)
																		granisetron	—	—
																			nalmefene	—
																				low-dose propofol

Effect sizes represent summary odds ratios (OR) and 95% confidence intervals. OR<1 indicate that the intervention specified in the column got more prophylactic effect than that specified in the row, which values are in bold.

Abbreviation: NS: normal saline; EIM: etomidate-induced myoclonus.

Table S6C Results of Bayesian network meta-analysis of the duration of EIM (secondary outcome)

NS	-58.04 (-134.21, 17.89)	-70.49 (-167.54, 17.20)	-7.14 (-64.66, 39.37)	-2.18 (-71.75, 62.57)	-37.95 (-116.91, 37.69)
	Remifentanil	-13.24 (-137.53, 105.97)	50.39 (-43.74, 141.54)	55.16 (-46.99, 156.35)	19.75 (-87.89, 129.64)
		Sufentanil	63.13 (-9.39, 138.93)	67.21 (-28.95, 172.90)	32.90 (-84.48, 156.67)
			Midazolam	4.26 (-61.27, 75.63)	-29.76 (-120.96, 64.58)
				Lidocaine	-34.80 (-135.24, 65.13)
					Alfentanil

Effect sizes represent summary MD (mean difference) and 95% confidence intervals (CI). When 95% CI include null (0) indicate no statistical significance.

Abbreviation: NS: normal saline; EIM: etomidate-induced myoclonus.

Table S7A Node-splitting analysis of the overall risk of EIM (primary outcome)

Name	Direct Effect	Indirect Effect	Overall	P-Value
A, C	-1.99 (-2.94, -1.07)	-1.37 (-4.01, 1.22)	-1.71 (-2.56, -0.88)	0.65
A, D	-2.04 (-2.98, -1.13)	-1.66 (-3.17, -0.05)	-2.01 (-2.77, -1.26)	0.66
A, E	-3.64 (-4.76, -2.56)	-2.51 (-4.35, -0.67)	-3.24 (-4.19, -2.36)	0.29
A, F	-3.09 (-4.30, -1.90)	-4.92 (-7.53, -2.41)	-3.35 (-4.45, -2.28)	0.19
A, G	-2.06 (-2.96, -1.21)	-2.52 (-4.03, -1.01)	-2.12 (-2.86, -1.39)	0.60
A, I	-1.93 (-3.07, -0.79)	-2.57 (-4.31, -0.82)	-1.97 (-2.88, -1.05)	0.53
A, J	-3.08 (-4.13, -2.03)	-0.74 (-3.01, 1.50)	-2.61 (-3.57, -1.65)	0.06
A, L	-1.82 (-3.39, -0.26)	-1.07 (-2.95, 0.79)	-1.66 (-2.84, -0.50)	0.53
A, M	-2.02 (-3.61, -0.44)	-3.77 (-5.50, -2.03)	-2.82 (-4.03, -1.62)	0.14
A, N	-2.64 (-4.01, -1.33)	-3.07 (-5.60, -0.54)	-2.75 (-3.91, -1.60)	0.77
B, C	3.38 (1.59, 5.52)	2.10 (0.40, 4.01)	2.50 (1.08, 4.18)	0.23
C, E	-2.15 (-4.47, -0.29)	-1.16 (-2.55, 0.22)	-1.52 (-2.71, -0.39)	0.38
C, F	-0.67 (-3.57, 2.11)	-1.93 (-3.40, -0.50)	-1.63 (-2.96, -0.31)	0.41
C, N	-1.31 (-3.62, 1.00)	-0.89 (-2.48, 0.73)	-1.03 (-2.31, 0.28)	0.77
D, E	-0.40 (-1.93, 1.14)	-1.97 (-3.45, -0.54)	-1.23 (-2.34, -0.19)	0.14
D, F	-2.75 (-5.19, -0.36)	-0.86 (-2.29, 0.57)	-1.34 (-2.58, -0.13)	0.18
D, G	-0.27 (-2.56, 1.96)	-0.11 (-1.19, 0.98)	-0.11 (-1.11, 0.87)	0.90
D, I	0.68 (-1.55, 2.95)	-0.17 (-1.46, 1.08)	0.04 (-1.07, 1.14)	0.51
D, J	-0.57 (-2.88, 1.74)	-0.48 (-1.84, 0.85)	-0.60 (-1.78, 0.57)	0.95
D, L	0.90 (-1.21, 3.05)	-0.09 (-1.80, 1.61)	0.35 (-0.96, 1.65)	0.45
D, M	-1.33 (-3.41, 0.67)	0.05 (-1.69, 1.77)	-0.81 (-2.14, 0.49)	0.31
E, F	1.14 (-1.93, 4.24)	-0.35 (-1.84, 1.17)	-0.10 (-1.46, 1.31)	0.36
E, L	1.60 (-0.54, 3.77)	1.12 (-0.64, 2.92)	1.59 (0.23, 2.96)	0.73
E, M	-0.59 (-2.61, 1.41)	1.32 (-0.49, 3.11)	0.42 (-0.92, 1.84)	0.15
G, I	-0.31 (-1.54, 1.00)	1.01 (-0.36, 2.38)	0.15 (-0.82, 1.13)	0.16
G, J	1.15 (-0.95, 3.23)	-1.07 (-2.36, 0.22)	-0.49 (-1.63, 0.63)	0.08
K, L	-1.86 (-4.12, 0.33)	0.16 (-1.90, 2.20)	-0.64 (-2.35, 0.99)	0.17
L, M	-2.13 (-4.19, -0.12)	-0.23 (-2.18, 1.76)	-1.16 (-2.67, 0.37)	0.16

Table S7B Node-splitting analysis of the risk of EIM at a mild intensity level (secondary outcome)

Name	Direct Effect	Indirect Effect	Overall	P-Value
A, C	-0.79 (-1.73, 0.11)	0.64 (-1.97, 3.54)	-0.48 (-1.31, 0.37)	0.31
A, D	0.03 (-0.73, 0.78)	-2.84 (-4.79, -1.18)	-0.51 (-1.25, 0.19)	**0.00**
A, E	-1.52 (-2.55, -0.62)	0.53 (-1.14, 2.20)	-1.03 (-1.91, -0.22)	**0.03**
A, F	-1.54 (-2.74, -0.44)	23.19 (1.14, 81.60)	-1.21 (-2.30, -0.13)	**0.00**
A, G	-0.81 (-1.59, -0.06)	0.56 (-0.88, 2.01)	-0.51 (-1.19, 0.17)	0.09
A, I	-0.57 (-1.64, 0.44)	-2.08 (-3.69, -0.59)	-0.96 (-1.86, -0.08)	0.10
A, J	-0.89 (-1.94, 0.17)	-1.43 (-4.16, 1.06)	-0.96 (-1.94, -0.02)	0.70
A, L	-2.44 (-6.13, 0.17)	-1.34 (-3.27, 0.52)	-1.75 (-3.39, -0.29)	0.50
A, M	0.18 (-1.41, 1.69)	-0.30 (-2.06, 1.48)	-0.02 (-1.21, 1.09)	0.69
A, N	-0.98 (-2.28, 0.26)	-2.20 (-4.95, 0.22)	-1.22 (-2.34, -0.07)	0.39
B, C	1.93 (0.08, 4.14)	1.89 (0.22, 3.85)	1.80 (0.34, 3.54)	0.97
C, E	-1.66 (-3.90, 0.17)	0.09 (-1.20, 1.33)	-0.54 (-1.72, 0.54)	0.11
C, F	-33.31 (-108.54, -1.03)	-0.63 (-2.04, 0.78)	-0.73 (-2.14, 0.59)	**0.05**
C, N	-1.61 (-4.22, 0.73)	-0.37 (-1.91, 1.21)	-0.73 (-2.04, 0.54)	0.38
D, E	0.45 (-0.97, 1.85)	-1.28 (-2.60, -0.03)	-0.51 (-1.53, 0.48)	0.07
D, F	23.31 (1.76, 68.16)	-1.15 (-2.54, 0.17)	-0.70 (-1.98, 0.56)	**0.00**
D, G	-0.33 (-2.40, 1.70)	0.07 (-0.95, 1.13)	0.01 (-0.92, 0.94)	0.72
D, I	0.39 (-1.71, 2.49)	-0.71 (-1.95, 0.53)	-0.44 (-1.53, 0.65)	0.35
D, J	-0.21 (-2.46, 2.02)	-0.60 (-1.95, 0.69)	-0.44 (-1.58, 0.70)	0.74
D, L	-0.29 (-2.41, 1.89)	-2.20 (-5.88, 0.49)	-1.24 (-2.89, 0.31)	0.28
D, M	0.96 (-0.95, 3.02)	0.73 (-0.96, 2.44)	0.49 (-0.76, 1.73)	0.85
E, F	-55.80 (-131.16, -3.91)	-0.09 (-1.48, 1.37)	-0.20 (-1.56, 1.21)	**0.01**
E, L	-1.05 (-3.21, 0.95)	-1.75 (-5.37, 0.98)	-0.73 (-2.40, 0.85)	0.69
E, M	0.20 (-1.78, 2.10)	1.24 (-0.53, 3.02)	0.99 (-0.26, 2.30)	0.41
G, I	-1.30 (-2.55, -0.10)	0.66 (-0.65, 1.95)	-0.44 (-1.40, 0.49)	**0.03**
G, J	-0.87 (-3.66, 1.65)	-0.35 (-1.61, 0.92)	-0.45 (-1.57, 0.67)	0.72
K, L	-3.18 (-7.07, -0.57)	-1.67 (-4.14, 0.62)	-2.17 (-4.14, -0.45)	0.40
L, M	1.41 (-0.67, 3.54)	1.98 (-0.17, 4.28)	1.74 (0.07, 3.46)	0.67

Table S7C Node-splitting analysis of the risk of EIM at moderate-to-severe intensity level (secondary outcome)

Name	Direct Effect	Indirect Effect	Overall	P-Value
A, C	-2.06 (-3.35, -0.87)	-2.33 (-5.81, 1.09)	-1.84 (-2.98, -0.75)	0.89
A, D	-2.42 (-3.65, -1.27)	-1.07 (-3.08, 0.87)	-2.11 (-3.15, -1.17)	0.23
A, E	-5.36 (-7.89, -3.47)	-2.67 (-5.06, -0.35)	-4.00 (-5.60, -2.70)	0.08
A, F	-2.31 (-3.77, -0.93)	-5.62 (-8.69, -2.71)	-2.85 (-4.21, -1.55)	**0.05**
A, G	-2.51 (-3.77, -1.38)	-3.48 (-5.41, -1.59)	-2.70 (-3.75, -1.72)	0.39
A, I	-1.73 (-3.21, -0.30)	-1.87 (-4.29, 0.49)	-1.66 (-2.86, -0.50)	0.92
A, J	-3.43 (-4.89, -2.03)	-1.01 (-3.98, 1.89)	-2.88 (-4.20, -1.61)	0.14
A, M	-1.95 (-3.86, -0.07)	-4.54 (-6.90, -2.30)	-3.01 (-4.55, -1.52)	0.08
A, N	-3.05 (-4.99, -1.29)	-2.53 (-5.69, 0.58)	-2.91 (-4.53, -1.40)	0.78
B, C	3.60 (1.34, 6.44)	1.54 (-0.78, 4.05)	2.45 (0.58, 4.68)	0.13
C, E	-45.96 (-142.82, -4.26)	-1.67 (-3.63, 0.19)	-2.17 (-4.04, -0.51)	**0.01**
C, F	-0.20 (-3.58, 3.08)	-1.25 (-3.18, 0.63)	-1.01 (-2.68, 0.65)	0.58
C, N	-0.72 (-3.71, 2.23)	-1.26 (-3.51, 0.90)	-1.07 (-2.83, 0.63)	0.77
D, E	-0.51 (-2.41, 1.54)	-59.78 (-164.85, -6.39)	-1.90 (-3.54, -0.42)	**0.00**
D, F	-3.21 (-6.04, -0.43)	0.18 (-1.50, 1.87)	-0.74 (-2.25, 0.80)	**0.04**
D, G	-0.01 (-3.07, 3.02)	-0.73 (-2.19, 0.77)	-0.59 (-1.92, 0.77)	0.66
D, I	0.70 (-2.27, 3.72)	0.33 (-1.30, 2.04)	0.45 (-0.98, 1.93)	0.82
D, J	-1.42 (-5.56, 2.07)	-0.48 (-2.20, 1.28)	-0.76 (-2.32, 0.80)	0.63
D, L	0.70 (-2.06, 3.42)	0.27 (-2.61, 3.19)	0.55 (-1.31, 2.42)	0.83
D, M	-1.90 (-4.37, 0.61)	0.32 (-1.77, 2.43)	-0.90 (-2.55, 0.79)	0.17
E, F	2.14 (-1.55, 6.17)	0.96 (-1.04, 3.08)	1.16 (-0.64, 3.15)	0.55
E, L	1.88 (-0.85, 4.55)	2.14 (-0.89, 5.34)	2.45 (0.50, 4.53)	0.90
E, M	-0.68 (-3.19, 1.83)	2.34 (0.03, 4.83)	1.01 (-0.77, 2.95)	0.08
G, I	0.71 (-1.03, 2.48)	1.68 (-0.17, 3.54)	1.04 (-0.24, 2.35)	0.44
G, J	1.44 (-1.28, 4.15)	-0.88 (-2.65, 0.85)	-0.18 (-1.67, 1.31)	0.16
K, L	-0.45 (-3.29, 2.37)	0.42 (-2.72, 3.44)	0.15 (-2.07, 2.33)	0.67
L, M	-2.54 (-5.11, 0.04)	-0.01 (-2.55, 2.63)	-1.45 (-3.49, 0.62)	0.15

Table S7E Node-splitting analysis of the risk of adverse events (secondary outcome)

Name	Direct Effect	Indirect Effect	Overall	P-Value
A, C	1.78 (-0.56, 5.23)	-0.01 (-4.24, 4.23)	1.26 (-0.55, 3.87)	0.41
A, G	0.08 (-1.46, 1.90)	-1.01 (-5.67, 3.46)	-0.02 (-1.35, 1.63)	0.62
A, I	-1.15 (-3.91, 1.48)	-0.30 (-4.36, 3.86)	-0.69 (-2.65, 1.44)	0.69
A, N	-0.01 (-2.18, 2.14)	1.90 (-2.21, 7.11)	0.33 (-1.42, 2.28)	0.38
B, C	-0.25 (-2.84, 2.30)	-17.68 (-46.27, -2.58)	-1.24 (-3.87, 1.12)	**0.01**
C, N	0.00 (-3.71, 3.76)	-1.97 (-6.37, 1.10)	-0.94 (-3.62, 1.17)	0.38
D, G	-0.53 (-3.54, 2.64)	1.57 (-0.72, 4.22)	0.62 (-1.59, 3.07)	0.23
D, I	-0.32 (-4.03, 3.35)	0.17 (-3.25, 3.87)	-0.05 (-2.53, 2.54)	0.82
G, I	-0.16 (-2.83, 2.75)	-1.02 (-4.94, 2.56)	-0.67 (-2.77, 1.30)	0.68

Significant values ($P \leq 0.05$) are in bold, indicating a significant inconsistency between the direct effect and indirect effects.

EIM: etomidate-induced myoclonus. A: Normal saline; B: Oxycodone; C: Fentanyl; D: Midazolam; E: Remifentanil; F: Sufentanil; G: Dexmedetomidine; H: Thiopental; I: Lidocaine; J: Butorphanol; K: Ketamine; L: Magnesium sulfate M: Low-dose etomidate; N: Nalbuphine; O: Rocuronium; P: Alfentanil; Q: Tramadol; R: Vecuronium; S: Granisetron; T: Nalmefene; U: Low-dose propofol.

Table S8 Results of SUCRA analysis of the different outcomes

Treatment	The overall risk of EIM	The risk of EIM at a mild intensity level	The risk of EIM at moderate-to-severe intensity level
	SUCRA	SUCRA	SUCRA
NS	1.9	20.4	2
Oxycodone	89.7	89.2	81.5
Fentanyl	29.9	39.2	29.8
Midazolam	38.7	40.5	36.8
Remifentanil	74.9	60.3	79.7
Sufentanil	76.5	65.8	56.7
Dexmedetomidine	42.9	40.2	53.5
Thiopental	29.5	34.9	35.4
Lidocaine	37.7	58.0	25.7
Butorphanol	58.2	57.7	57.5
Ketamine	16.3	15.3	29.2
Magnesium sulfate	29.5	78.6	25.5
Low-dose etomidate	63.5	24.8	60.2
Nalbuphine	61.5	65.9	57.7
Rocuronium	32.8	45.6	49.8
Alfentanil	82.8	72.0	58.9
Tramadol	36.1	2.9	40.8
Vecuronium	36.4	31.5	39.1
Granisetron	94.4	93.1	96.8
Nalmefene	75.8	61.1	97.6
Low-dose propofol	40.8	53.0	36

The SUCRA cumulative probabilities was 100 when intervention was certain to be the best.

Abbreviation: NS: normal saline; EIM: etomidate-induced myoclonus. SUCRA: surface under the cumulative ranking curve.

Table S9 The overall risk of EIM (primary outcome): SUCRA analysis when trials at high risk of bias were excluded

Treatment	SUCRA	PrBest	MeanRank
NS	2.7	0.0	19.5
Oxycodone	89.6	17.3	3.0
Fentanyl	34.0	0.0	13.5
Midazolam	42.5	0.0	11.9
Remifentanil	73.3	0.4	6.1
Sufentanil	75.0	0.5	5.8
Dexmedetomidine	34.1	0.0	13.5
Thiopental	28.1	0.1	14.7
Lidocaine	32.0	0.0	13.9
Butorphanol	50.0	0.1	10.5
Ketamine	19.3	0.0	16.3
Magnesium sulfate	33.2	0.0	13.7
Low-dose etomidate	63.3	0.3	8.0
Nalbuphine	62.3	0.9	8.2
Rocuronium	35.3	0.3	13.3
Alfentanil	81.2	19.9	4.6
Tramadol	38.1	1.0	12.8
Vecuronium	38.5	0.7	12.7
Granisetron	93.1	51.7	2.3
Nalmefene	74.5	6.9	5.8

The SUCRA cumulative probabilities was 100 when intervention was certain to be the best.

Abbreviation: NS: normal saline; EIM: etomidate-induced myoclonus. SUCRA: surface under the cumulative ranking curve.

Table S10 The information of the drug-related adverse events

Interventions	Nausea/vomiting	Dizziness	Coughing	Headache	Injection pain	Respiratory depression	Bradycardia	hypotension	Myalgia
oxycodone		√							
alfentanil			√			√	√	√	
fentanyl	√	√	√				√	√	
sufentanil	√	√			√	√			
remifentanil							√		
midazolam	√			√	√	√			√
lidocaine	√			√	√		√	√	√
ketamine	√				√	√			
nalbuphine	√				√		√	√	
nalmefene	√	√							
butorphanol	√	√							
dexmedetomidine	√		√	√	√		√	√	
low-dose etomidate					√				
low-dose propofol					√				
magnesium sulfate					√	√			
thiopental	√		√	√					
vecuronium					√				
rocuronium					√				
tramadol	√	√				√			

Table S11 Results of the meta-regression analysis

Covariates	Ages	Country	Sample size	Female ratio	Doses of etomidate for induction of anesthesia
Coef.	-0.052	0.084	0.009	-0.007	0.35
Std. Err.	0.031	0.138	0.005	0.013	0.59
p >|t|	0.08	0.54	0.11	0.58	0.56

Coef., coefficient; Std. Err., standard error

Table S12 GRADE for the primary outcomes

Comparison	Risk of bias	Indirectness	Imprecision	Inconsistency		Publication bias	GRADE
				Heterogeneity	Incoherence		
	Mixed evidence						
Butorphanol: Dexmedetomidine	Downgrade	No downgrade	Downgrade	No downgrade	No downgrade	No downgrade	Low
Butorphanol: Midazolam	Downgrade	No downgrade	Downgrade	No downgrade	No downgrade	No downgrade	Low
Butorphanol: NS	Downgrade	No downgrade	No downgrade	No downgrade	No downgrade	No downgrade	Moderate
Dexmedetomidine: Lidocaine	Downgrade	No downgrade	Downgrade	Downgrade	No downgrade	No downgrade	Very low
Dexmedetomidine: Midazolam	Downgrade	No downgrade	Downgrade	No downgrade	No downgrade	No downgrade	Low
Dexmedetomidine: NS	Downgrade	No downgrade	No downgrade	No downgrade	No downgrade	No downgrade	Moderate
Dexmedetomidine: Thiopental	No downgrade	No downgrade	Downgrade	No downgrade	No downgrade	No downgrade	Moderate
Fentanyl: NS	Downgrade	No downgrade	No downgrade	No downgrade	No downgrade	No downgrade	Moderate
Fentanyl: Nalbuphine	Downgrade	No downgrade	Downgrade	Downgrade	No downgrade	No downgrade	Very low
Fentanyl: Nalmefene	No downgrade	No downgrade	Downgrade	Downgrade	No downgrade	No downgrade	Low
Fentanyl: Oxycodone	Downgrade	No downgrade	Downgrade	Downgrade	No downgrade	No downgrade	Very low
Fentanyl: Remifentanil	Downgrade	No downgrade	No downgrade	Downgrade	No downgrade	No downgrade	Low
Fentanyl: Sufentanil	Downgrade	No downgrade	No downgrade	Downgrade	No downgrade	No downgrade	Low
Ketamine: Magnesium sulfate	No downgrade	No downgrade	Downgrade	Downgrade	No downgrade	No downgrade	Low
Ketamine: NS	No downgrade	No downgrade	Downgrade	Downgrade	No downgrade	No downgrade	Low
Lidocaine: Midazolam	Downgrade	No downgrade	Downgrade	No downgrade	No downgrade	No downgrade	Low
Lidocaine: NS	Downgrade	No downgrade	No downgrade	Downgrade	No downgrade	No downgrade	Low
Low-dose etomidate: Magnesium sulfate	No downgrade	No downgrade	Downgrade	Downgrade	No downgrade	No downgrade	Low
Low-dose etomidate: Midazolam	No downgrade	No downgrade	Downgrade	No downgrade	No downgrade	No downgrade	Moderate
Low-dose etomidate: NS	Downgrade	No downgrade	No downgrade	Downgrade	No downgrade	No downgrade	Low
Low-dose etomidate: Remifentanil	No downgrade	No downgrade	Downgrade	No downgrade	No downgrade	No downgrade	Moderate
Magnesium sulfate: Midazolam	No downgrade	No downgrade	Downgrade	No downgrade	No downgrade	No downgrade	Moderate
Magnesium sulfate: NS	No downgrade	No downgrade	No downgrade	Downgrade	No downgrade	No downgrade	Moderate
Magnesium sulfate: Remifentanil	No downgrade	No downgrade	No downgrade	Downgrade	No downgrade	No downgrade	Moderate
Midazolam: NS	Downgrade	No downgrade	No downgrade	Downgrade	No downgrade	No downgrade	Low
Midazolam: Remifentanil	No downgrade	No downgrade	No downgrade	Downgrade	No downgrade	No downgrade	Moderate

Midazolam: Sufentanil	Downgrade	No downgrade	No downgrade	Downgrade	No downgrade	No downgrade	Low
Nalbuphine: NS	Downgrade	No downgrade	No downgrade	No downgrade	No downgrade	No downgrade	Moderate
NS: Oxycodone	Downgrade	No downgrade	No downgrade	Downgrade	No downgrade	No downgrade	Low
NS: Remifentanil	Downgrade	No downgrade	No downgrade	No downgrade	No downgrade	No downgrade	Moderate
NS: Sufentanil	Downgrade	No downgrade	No downgrade	Downgrade	No downgrade	No downgrade	Low
Remifentanil: Sufentanil	No downgrade	No downgrade	Downgrade	No downgrade	No downgrade	No downgrade	Moderate
	**Direct evidence**						
Granisetron: NS	No downgrade	No downgrade	Downgrade	No downgrade	No downgrade	No downgrade	Moderate
Alfentanil: NS	No downgrade	No downgrade	Downgrade	No downgrade	No downgrade	No downgrade	Moderate
Low-dose propofol: NS	Downgrade	No downgrade	Downgrade	Downgrade	No downgrade	No downgrade	Very low
NS: Rocuronium	No downgrade	No downgrade	Downgrade	Downgrade	No downgrade	No downgrade	Low
NS: Tramadol	Downgrade	No downgrade	Downgrade	No downgrade	No downgrade	No downgrade	Low
NS: Vecuronium	No downgrade	No downgrade	Downgrade	No downgrade	No downgrade	No downgrade	Moderate
NS: Thiopental	No downgrade	No downgrade	Downgrade	Downgrade	No downgrade	No downgrade	Low
Nalmefene: NS	No downgrade	No downgrade	Downgrade	No downgrade	No downgrade	No downgrade	Moderate
Granisetron: Sufentanil	No downgrade	No downgrade	Downgrade	No downgrade	No downgrade	No downgrade	Moderate
	**Indirect evidence**						
Alfentanil: Butorphanol	Downgrade	Downgrade	Downgrade	No downgrade	No downgrade	No downgrade	Very low
Alfentanil: Dexmedetomidine	Downgrade	Downgrade	Downgrade	No downgrade	No downgrade	No downgrade	Very low
Alfentanil: Fentanyl	No downgrade	Downgrade	Downgrade	No downgrade	No downgrade	No downgrade	Low
Alfentanil: Granisetron	No downgrade	Downgrade	Downgrade	No downgrade	No downgrade	No downgrade	Low
Alfentanil: Ketamine	No downgrade	Downgrade	No downgrade	Downgrade	No downgrade	No downgrade	Low
Alfentanil: Lidocaine	Downgrade	Downgrade	Downgrade	No downgrade	No downgrade	No downgrade	Very low
Alfentanil: Low-dose etomidate	No downgrade	Downgrade	Downgrade	No downgrade	No downgrade	No downgrade	Low
Alfentanil: Low-dose propofol	Downgrade	Downgrade	Downgrade	No downgrade	No downgrade	No downgrade	Very low
Alfentanil: Magnesium sulfate	No downgrade	Downgrade	Downgrade	Downgrade	No downgrade	No downgrade	Very low
Alfentanil: Midazolam	Downgrade	Downgrade	Downgrade	No downgrade	No downgrade	No downgrade	Very low
Alfentanil: Nalbuphine	Downgrade	Downgrade	Downgrade	No downgrade	No downgrade	No downgrade	Very low
Alfentanil: Nalmefene	No downgrade	Downgrade	Downgrade	No downgrade	No downgrade	No downgrade	Low
Alfentanil: Oxycodone	No downgrade	Downgrade	Downgrade	No downgrade	No downgrade	No downgrade	Low

Alfentanil: Remifentanil	No downgrade	Downgrade	Downgrade	No downgrade	No downgrade	No downgrade	Low
Alfentanil: Rocuronium	No downgrade	Downgrade	Downgrade	No downgrade	No downgrade	No downgrade	Low
Alfentanil: Sufentanil	No downgrade	Downgrade	Downgrade	No downgrade	No downgrade	No downgrade	Low
Alfentanil: Thiopental	No downgrade	Downgrade	Downgrade	No downgrade	No downgrade	No downgrade	Low
Alfentanil: Tramadol	Downgrade	Downgrade	Downgrade	No downgrade	No downgrade	No downgrade	Very low
Alfentanil: Vecuronium	No downgrade	Downgrade	Downgrade	No downgrade	No downgrade	No downgrade	Low
Butorphanol: Fentanyl	Downgrade	Downgrade	Downgrade	No downgrade	No downgrade	No downgrade	Very low
Butorphanol: Granisetron	Downgrade	Downgrade	Downgrade	Downgrade	No downgrade	No downgrade	Very low
Butorphanol: Ketamine	No downgrade	Downgrade	No downgrade	Downgrade	No downgrade	No downgrade	Low
Butorphanol: Lidocaine	Downgrade	Downgrade	Downgrade	No downgrade	No downgrade	No downgrade	Very low
Butorphanol: Low-dose etomidate	Downgrade	Downgrade	Downgrade	No downgrade	No downgrade	No downgrade	Very low
Butorphanol: Low-dose propofol	Downgrade	Downgrade	Downgrade	No downgrade	No downgrade	No downgrade	Very low
Butorphanol: Magnesium sulfate	Downgrade	Downgrade	Downgrade	No downgrade	No downgrade	No downgrade	Very low
Butorphanol: Nalbuphine	Downgrade	Downgrade	Downgrade	No downgrade	No downgrade	No downgrade	Very low
Butorphanol: Nalmefene	Downgrade	Downgrade	Downgrade	No downgrade	No downgrade	No downgrade	Very low
Butorphanol: Oxycodone	Downgrade	Downgrade	Downgrade	No downgrade	No downgrade	No downgrade	Very low
Butorphanol: Remifentanil	Downgrade	Downgrade	Downgrade	No downgrade	No downgrade	No downgrade	Very low
Butorphanol: Rocuronium	Downgrade	Downgrade	Downgrade	No downgrade	No downgrade	No downgrade	Very low
Butorphanol: Sufentanil	Downgrade	Downgrade	Downgrade	No downgrade	No downgrade	No downgrade	Very low
Butorphanol: Thiopental	No downgrade	Downgrade	Downgrade	No downgrade	No downgrade	No downgrade	Low
Butorphanol: Tramadol	Downgrade	Downgrade	Downgrade	No downgrade	No downgrade	No downgrade	Very low
Butorphanol: Vecuronium	Downgrade	Downgrade	Downgrade	No downgrade	No downgrade	No downgrade	Very low
Dexmedetomidine: Fentanyl	Downgrade	Downgrade	Downgrade	No downgrade	No downgrade	No downgrade	Very low
Dexmedetomidine: Granisetron	Downgrade	Downgrade	No downgrade	No downgrade	No downgrade	No downgrade	Low
Dexmedetomidine: Ketamine	Downgrade	Downgrade	Downgrade	No downgrade	No downgrade	No downgrade	Very low
Dexmedetomidine: Low-dose etomidate	Downgrade	Downgrade	Downgrade	No downgrade	No downgrade	No downgrade	Very low
Dexmedetomidine: Low-dose propofol	Downgrade	Downgrade	Downgrade	No downgrade	No downgrade	No downgrade	Very low
Dexmedetomidine: Magnesium sulfate	Downgrade	Downgrade	Downgrade	No downgrade	No downgrade	No downgrade	Very low
Dexmedetomidine: Nalbuphine	Downgrade	Downgrade	Downgrade	No downgrade	No downgrade	No downgrade	Very low
Dexmedetomidine: Nalmefene	Downgrade	Downgrade	Downgrade	No downgrade	No downgrade	No downgrade	Very low

Dexmedetomidine: Oxycodone	Downgrade	Downgrade	Downgrade	No downgrade	No downgrade	No downgrade	Very low
Dexmedetomidine: Remifentanil	Downgrade	Downgrade	No downgrade	Downgrade	No downgrade	No downgrade	Very low
Dexmedetomidine: Rocuronium	Downgrade	Downgrade	Downgrade	No downgrade	No downgrade	No downgrade	Very low
Dexmedetomidine: Sufentanil	Downgrade	Downgrade	No downgrade	Downgrade	No downgrade	No downgrade	Very low
Dexmedetomidine: Tramadol	Downgrade	Downgrade	Downgrade	No downgrade	No downgrade	No downgrade	Very low
Dexmedetomidine: Vecuronium	Downgrade	Downgrade	Downgrade	No downgrade	No downgrade	No downgrade	Very low
Fentanyl: Granisetron	No downgrade	Downgrade	No downgrade	No downgrade	No downgrade	No downgrade	Moderate
Fentanyl: Ketamine	No downgrade	Downgrade	Downgrade	No downgrade	No downgrade	No downgrade	Low
Fentanyl: Lidocaine	Downgrade	Downgrade	Downgrade	No downgrade	No downgrade	No downgrade	Very low
Fentanyl: Low-dose etomidate	Downgrade	Downgrade	Downgrade	No downgrade	No downgrade	No downgrade	Very low
Fentanyl: Low-dose propofol	Downgrade	Downgrade	Downgrade	No downgrade	No downgrade	No downgrade	Very low
Fentanyl: Magnesium sulfate	Downgrade	Downgrade	Downgrade	No downgrade	No downgrade	No downgrade	Very low
Fentanyl: Midazolam	Downgrade	Downgrade	Downgrade	No downgrade	No downgrade	No downgrade	Very low
Fentanyl: Rocuronium	No downgrade	Downgrade	Downgrade	No downgrade	No downgrade	No downgrade	Low
Fentanyl: Thiopental	No downgrade	Downgrade	Downgrade	No downgrade	No downgrade	No downgrade	Low
Fentanyl: Tramadol	Downgrade	Downgrade	Downgrade	No downgrade	No downgrade	No downgrade	Very low
Fentanyl: Vecuronium	No downgrade	Downgrade	Downgrade	No downgrade	No downgrade	No downgrade	Low
Granisetron: Ketamine	No downgrade	Downgrade	No downgrade	No downgrade	No downgrade	No downgrade	Moderate
Granisetron: Lidocaine	No downgrade	Downgrade	No downgrade	No downgrade	No downgrade	No downgrade	Moderate
Granisetron: Low-dose etomidate	No downgrade	Downgrade	Downgrade	No downgrade	No downgrade	No downgrade	Low
Granisetron: Low-dose propofol	Downgrade	Downgrade	No downgrade	Downgrade	No downgrade	No downgrade	Very low
Granisetron: Magnesium sulfate	No downgrade	Downgrade	No downgrade	No downgrade	No downgrade	No downgrade	Moderate
Granisetron: Midazolam	Downgrade	Downgrade	No downgrade	No downgrade	No downgrade	No downgrade	Low
Granisetron: Nalbuphine	Downgrade	Downgrade	Downgrade	No downgrade	No downgrade	No downgrade	Very low
Granisetron: Nalmefene	No downgrade	Downgrade	Downgrade	No downgrade	No downgrade	No downgrade	Low
Granisetron: Oxycodone	No downgrade	Downgrade	Downgrade	No downgrade	No downgrade	No downgrade	Low
Granisetron: Remifentanil	No downgrade	Downgrade	Downgrade	No downgrade	No downgrade	No downgrade	Low
Granisetron: Rocuronium	No downgrade	Downgrade	No downgrade	Downgrade	No downgrade	No downgrade	Low
Granisetron: Thiopental	No downgrade	Downgrade	No downgrade	No downgrade	No downgrade	No downgrade	Moderate
Granisetron: Tramadol	Downgrade	Downgrade	No downgrade	Downgrade	No downgrade	No downgrade	Very low

Granisetron: Vecuronium	No downgrade	Downgrade	No downgrade	Downgrade	No downgrade	No downgrade	Low
Ketamine: Lidocaine	No downgrade	Downgrade	Downgrade	No downgrade	No downgrade	No downgrade	Low
Ketamine: Low-dose etomidate	No downgrade	Downgrade	No downgrade	Downgrade	No downgrade	No downgrade	Low
Ketamine: Low-dose propofol	Downgrade	Downgrade	Downgrade	No downgrade	No downgrade	No downgrade	Very low
Ketamine: Midazolam	Downgrade	Downgrade	Downgrade	No downgrade	No downgrade	No downgrade	Very low
Ketamine: Nalbuphine	Downgrade	Downgrade	No downgrade	Downgrade	No downgrade	No downgrade	Very low
Ketamine: Nalmefene	No downgrade	Downgrade	No downgrade	Downgrade	No downgrade	No downgrade	Low
Ketamine: Oxycodone	No downgrade	Downgrade	No downgrade	Downgrade	No downgrade	No downgrade	Low
Ketamine: Remifentanil	No downgrade	Downgrade	No downgrade	Downgrade	No downgrade	No downgrade	Low
Ketamine: Rocuronium	No downgrade	Downgrade	Downgrade	No downgrade	No downgrade	No downgrade	Low
Ketamine: Sufentanil	No downgrade	Downgrade	No downgrade	Downgrade	No downgrade	No downgrade	Low
Ketamine: Thiopental	No downgrade	Downgrade	Downgrade	No downgrade	No downgrade	No downgrade	Low
Ketamine: Tramadol	Downgrade	Downgrade	Downgrade	No downgrade	No downgrade	No downgrade	Very low
Ketamine: Vecuronium	No downgrade	Downgrade	Downgrade	No downgrade	No downgrade	No downgrade	Low
Lidocaine: Low-dose etomidate	Downgrade	Downgrade	Downgrade	No downgrade	No downgrade	No downgrade	Very low
Lidocaine: Low-dose propofol	Downgrade	Downgrade	Downgrade	No downgrade	No downgrade	No downgrade	Very low
Lidocaine: Magnesium sulfate	Downgrade	Downgrade	Downgrade	No downgrade	No downgrade	No downgrade	Very low
Lidocaine: Nalbuphine	Downgrade	Downgrade	Downgrade	No downgrade	No downgrade	No downgrade	Very low
Lidocaine: Nalmefene	Downgrade	Downgrade	Downgrade	No downgrade	No downgrade	No downgrade	Very low
Lidocaine: Oxycodone	Downgrade	Downgrade	Downgrade	No downgrade	No downgrade	No downgrade	Very low
Lidocaine: Remifentanil	Downgrade	Downgrade	No downgrade	Downgrade	No downgrade	No downgrade	Very low
Lidocaine: Rocuronium	Downgrade	Downgrade	Downgrade	No downgrade	No downgrade	No downgrade	Very low
Lidocaine: Sufentanil	Downgrade	Downgrade	No downgrade	Downgrade	No downgrade	No downgrade	Very low
Lidocaine: Thiopental	No downgrade	Downgrade	Downgrade	No downgrade	No downgrade	No downgrade	Low
Lidocaine: Tramadol	Downgrade	Downgrade	Downgrade	No downgrade	No downgrade	No downgrade	Very low
Lidocaine: Vecuronium	Downgrade	Downgrade	Downgrade	No downgrade	No downgrade	No downgrade	Very low
Low-dose etomidate: Low-dose propofol	Downgrade	Downgrade	Downgrade	No downgrade	No downgrade	No downgrade	Very low
Low-dose etomidate: Nalbuphine	Downgrade	Downgrade	Downgrade	No downgrade	No downgrade	No downgrade	Very low
Low-dose etomidate: Nalmefene	No downgrade	Downgrade	Downgrade	No downgrade	No downgrade	No downgrade	Low
Low-dose etomidate: Oxycodone	Downgrade	Downgrade	Downgrade	No downgrade	No downgrade	No downgrade	Very low

Low-dose etomidate: Rocuronium	No downgrade	Downgrade	Downgrade	No downgrade	No downgrade	No downgrade	Low
Low-dose etomidate: Sufentanil	No downgrade	Downgrade	Downgrade	No downgrade	No downgrade	No downgrade	Low
Low-dose etomidate: Thiopental	No downgrade	Downgrade	Downgrade	No downgrade	No downgrade	No downgrade	Low
Low-dose etomidate: Tramadol	Downgrade	Downgrade	Downgrade	No downgrade	No downgrade	No downgrade	Very low
Low-dose etomidate: Vecuronium	No downgrade	Downgrade	Downgrade	No downgrade	No downgrade	No downgrade	Low
Low-dose propofol: Magnesium sulfate	Downgrade	Downgrade	Downgrade	No downgrade	No downgrade	No downgrade	Very low
Low-dose propofol: Midazolam	Downgrade	Downgrade	Downgrade	No downgrade	No downgrade	No downgrade	Very low
Low-dose propofol: Nalbuphine	Downgrade	Downgrade	Downgrade	No downgrade	No downgrade	No downgrade	Very low
Low-dose propofol: Nalmefene	Downgrade	Downgrade	Downgrade	No downgrade	No downgrade	No downgrade	Very low
Low-dose propofol: Oxycodone	Downgrade	Downgrade	Downgrade	No downgrade	No downgrade	No downgrade	Very low
Low-dose propofol: Remifentanil	Downgrade	Downgrade	Downgrade	No downgrade	No downgrade	No downgrade	Very low
Low-dose propofol: Rocuronium	Downgrade	Downgrade	Downgrade	No downgrade	No downgrade	No downgrade	Very low
Low-dose propofol: Sufentanil	Downgrade	Downgrade	Downgrade	No downgrade	No downgrade	No downgrade	Very low
Low-dose propofol: Thiopental	Downgrade	Downgrade	Downgrade	No downgrade	No downgrade	No downgrade	Very low
Low-dose propofol: Tramadol	Downgrade	Downgrade	Downgrade	No downgrade	No downgrade	No downgrade	Very low
Low-dose propofol: Vecuronium	Downgrade	Downgrade	Downgrade	No downgrade	No downgrade	No downgrade	Very low
Magnesium sulfate: Nalbuphine	Downgrade	Downgrade	Downgrade	No downgrade	No downgrade	No downgrade	Very low
Magnesium sulfate: Nalmefene	No downgrade	Downgrade	Downgrade	Downgrade	No downgrade	No downgrade	Very low
Magnesium sulfate: Oxycodone	Downgrade	Downgrade	Downgrade	Downgrade	No downgrade	No downgrade	Very low
Magnesium sulfate: Rocuronium	No downgrade	Downgrade	Downgrade	No downgrade	No downgrade	No downgrade	Low
Magnesium sulfate: Sufentanil	No downgrade	Downgrade	No downgrade	Downgrade	No downgrade	No downgrade	Low
Magnesium sulfate: Thiopental	No downgrade	Downgrade	Downgrade	No downgrade	No downgrade	No downgrade	Low
Magnesium sulfate: Tramadol	Downgrade	Downgrade	Downgrade	No downgrade	No downgrade	No downgrade	Very low
Magnesium sulfate: Vecuronium	No downgrade	Downgrade	Downgrade	No downgrade	No downgrade	No downgrade	Low
Midazolam: Nalbuphine	Downgrade	Downgrade	Downgrade	No downgrade	No downgrade	No downgrade	Very low
Midazolam: Nalmefene	Downgrade	Downgrade	Downgrade	No downgrade	No downgrade	No downgrade	Very low
Midazolam: Oxycodone	Downgrade	Downgrade	Downgrade	Downgrade	No downgrade	No downgrade	Very low
Midazolam: Rocuronium	Downgrade	Downgrade	Downgrade	No downgrade	No downgrade	No downgrade	Very low
Midazolam: Thiopental	Downgrade	Downgrade	Downgrade	No downgrade	No downgrade	No downgrade	Very low
Midazolam: Tramadol	Downgrade	Downgrade	Downgrade	No downgrade	No downgrade	No downgrade	Very low

Midazolam: Vecuronium	Downgrade	Downgrade	Downgrade	No downgrade	No downgrade	No downgrade	Very low
Nalbuphine: Nalmefene	Downgrade	Downgrade	Downgrade	No downgrade	No downgrade	No downgrade	Very low
Nalbuphine: Oxycodone	Downgrade	Downgrade	Downgrade	No downgrade	No downgrade	No downgrade	Very low
Nalbuphine: Remifentanil	Downgrade	Downgrade	Downgrade	No downgrade	No downgrade	No downgrade	Very low
Nalbuphine: Rocuronium	Downgrade	Downgrade	Downgrade	No downgrade	No downgrade	No downgrade	Very low
Nalbuphine: Sufentanil	Downgrade	Downgrade	Downgrade	No downgrade	No downgrade	No downgrade	Very low
Nalbuphine: Thiopental	Downgrade	Downgrade	Downgrade	No downgrade	No downgrade	No downgrade	Very low
Nalbuphine: Tramadol	Downgrade	Downgrade	Downgrade	No downgrade	No downgrade	No downgrade	Very low
Nalbuphine: Vecuronium	Downgrade	Downgrade	Downgrade	No downgrade	No downgrade	No downgrade	Very low
Nalmefene: Oxycodone	No downgrade	Downgrade	Downgrade	No downgrade	No downgrade	No downgrade	Low
Nalmefene: Remifentanil	No downgrade	Downgrade	Downgrade	No downgrade	No downgrade	No downgrade	Low
Nalmefene: Rocuronium	No downgrade	Downgrade	Downgrade	No downgrade	No downgrade	No downgrade	Low
Nalmefene: Sufentanil	No downgrade	Downgrade	Downgrade	No downgrade	No downgrade	No downgrade	Low
Nalmefene: Thiopental	No downgrade	Downgrade	Downgrade	No downgrade	No downgrade	No downgrade	Low
Nalmefene: Tramadol	Downgrade	Downgrade	Downgrade	No downgrade	No downgrade	No downgrade	Very low
Nalmefene: Vecuronium	No downgrade	Downgrade	Downgrade	No downgrade	No downgrade	No downgrade	Low
Oxycodone: Remifentanil	Downgrade	Downgrade	Downgrade	No downgrade	No downgrade	No downgrade	Very low
Oxycodone: Rocuronium	No downgrade	Downgrade	Downgrade	No downgrade	No downgrade	No downgrade	Low
Oxycodone: Sufentanil	Downgrade	Downgrade	Downgrade	No downgrade	No downgrade	No downgrade	Very low
Oxycodone: Thiopental	Downgrade	Downgrade	Downgrade	No downgrade	No downgrade	No downgrade	Very low
Oxycodone: Tramadol	Downgrade	Downgrade	Downgrade	No downgrade	No downgrade	No downgrade	Very low
Oxycodone: Vecuronium	No downgrade	Downgrade	Downgrade	No downgrade	No downgrade	No downgrade	Low
Remifentanil: Rocuronium	No downgrade	Downgrade	Downgrade	No downgrade	No downgrade	No downgrade	Low
Remifentanil: Thiopental	No downgrade	Downgrade	Downgrade	No downgrade	No downgrade	No downgrade	Low
Remifentanil: Tramadol	Downgrade	Downgrade	Downgrade	No downgrade	No downgrade	No downgrade	Very low
Remifentanil: Vecuronium	No downgrade	Downgrade	Downgrade	No downgrade	No downgrade	No downgrade	Low
Rocuronium: Sufentanil	No downgrade	Downgrade	Downgrade	No downgrade	No downgrade	No downgrade	Low
Rocuronium: Thiopental	No downgrade	Downgrade	Downgrade	No downgrade	No downgrade	No downgrade	Low
Rocuronium: Tramadol	Downgrade	Downgrade	Downgrade	No downgrade	No downgrade	No downgrade	Very low
Rocuronium: Vecuronium	No downgrade	Downgrade	Downgrade	No downgrade	No downgrade	No downgrade	Low

Sufentanil: Thiopental	No downgrade	Downgrade	Downgrade	Downgrade	No downgrade	No downgrade	Very low
Sufentanil: Tramadol	Downgrade	Downgrade	Downgrade	No downgrade	No downgrade	No downgrade	Very low
Sufentanil: Vecuronium	No downgrade	Downgrade	Downgrade	No downgrade	No downgrade	No downgrade	Low
Thiopental: Tramadol	Downgrade	Downgrade	Downgrade	No downgrade	No downgrade	No downgrade	Very low
Thiopental: Vecuronium	No downgrade	Downgrade	Downgrade	No downgrade	No downgrade	No downgrade	Low
Tramadol: Vecuronium	Downgrade	Downgrade	Downgrade	No downgrade	No downgrade	No downgrade	Very low

We GRADE each network estimate according to the following criteria.

(1) Risk of bias: We used a weighted average score for each relative effect estimate according to the percentage contribution of studies at each bias level. We down graded by one level when the average score was contributions from some concerns or high RoB comparisons.

(2) Imprecision: We considered a clinically meaningful threshold for OR to be 0.80 or 1.25 and downgraded the estimate if the confidence interval (CI) fully inters ects with the clinical threshold difference (OR point estimate is 1 or more and the lower limit of its CI is below 0.80; or if the OR point estimate is less than 1 and t he upper limit of its CI is above 1.25). Additionally, although the estimate and CI were far from the clinically meaningful threshold, downgraded when confidence i nterval is relatively too large compared to NS or other active drugs.

(3) Inconsistency: We rated two concepts, heterogeneity and inconsistency, in this domain. For heterogeneity, we looked at the common tau^2 (0.58) and found that i t is low compared to the expected value as reported in the literature[1], so we did not downgrade the overall network estimate for heterogeneity. However, downgrad ed for heterogeneity if the results of the NMA confidence intervals and prediction intervals were inconsistent for each comparison. For inconsistency, we looked at the results of node splitting (Table S 7A) and downgraded the comparisons with important inconsistency ($p<0.05$). If the main results of the NMA were only from t he direct or indirect comparisons, the inconsistency may be considered as little impact on the NMA results, and then no downgraded for this domain.

(4) Indirectness: Considering that most comparisons include few studies, the review team decided to downgrade when the result was solely derived from indirect c omparisons because the evaluation of transitivity for such comparison is unclear.

(5) Publication bias: The comparison-adjusted funnel plot and Egger's test (p=0.08) for the overall risk of etomidate-induced myoclonus (Figure S 5A) did not sug gest presence of overall publication bias. Additionally, considering that there were not enough studies for each comparison to make funnel plot, the review team de cided not to downgrade for publication bias.

Reference

1. Turner, R. M., Davey, J., Clarke, M. J., Thompson, S. G., & Higgins, J. P.. (2012). Predicting the extent of heterogeneity in meta-analysis, using empirical data from th e cochrane database of systematic reviews. International Journal of Epidemiology, 41(3), 818-827. 10.1093/ije/dys041.

A.

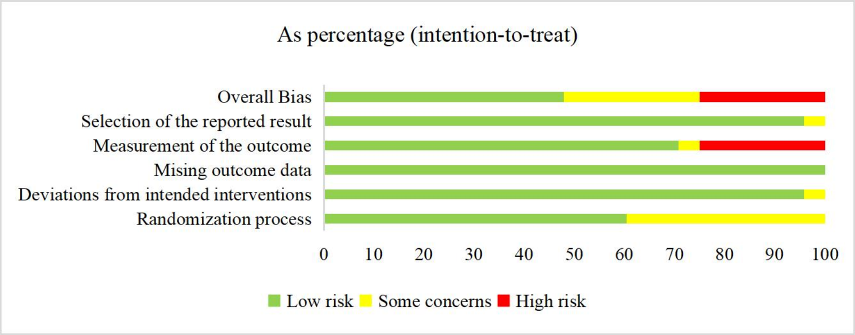

B.

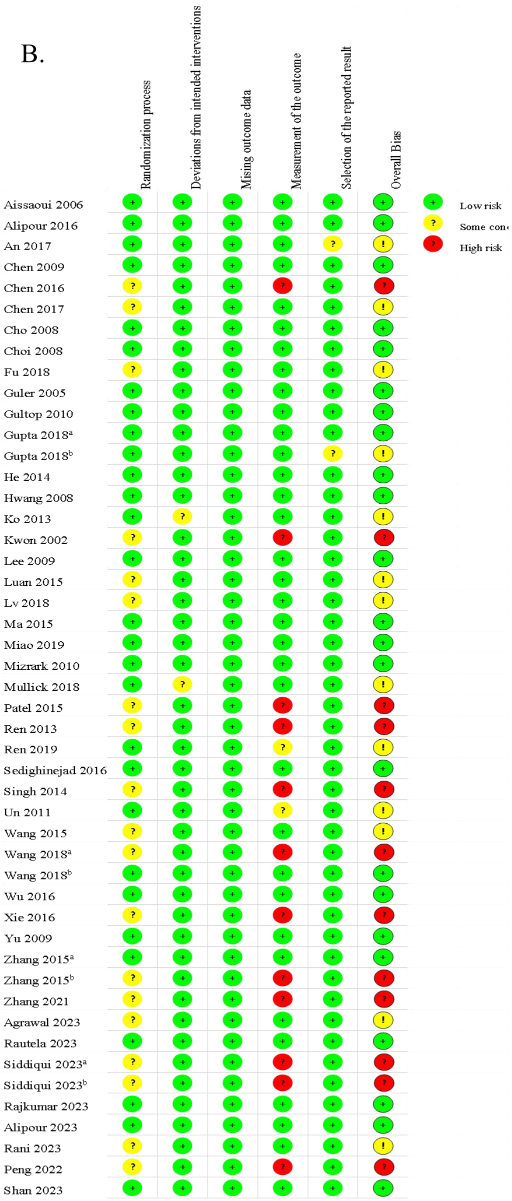

Figure S1 Summary risk of bias of the included studies. (A) Risk of bias graph. (B) Risk of b ias summary

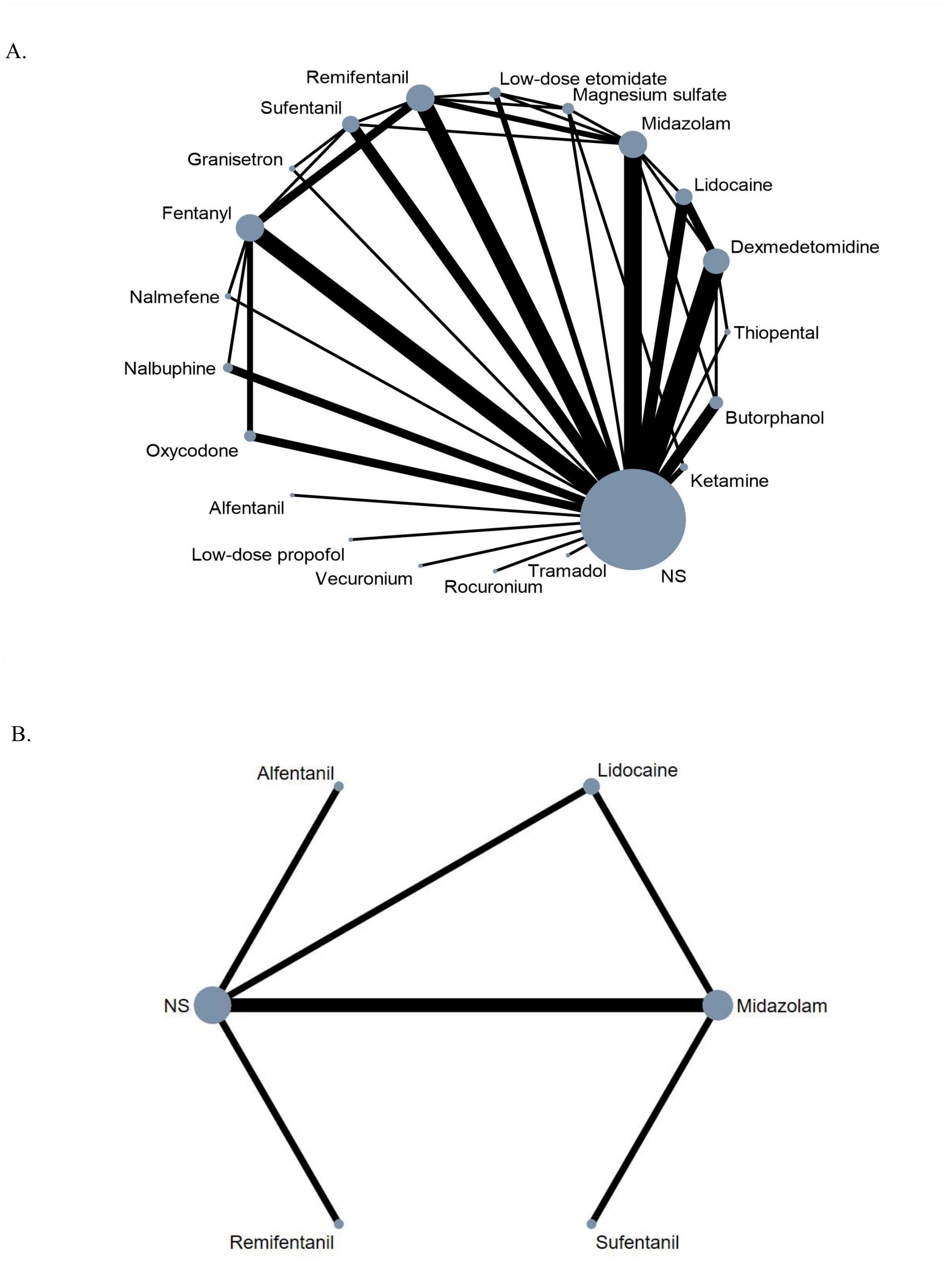

Figure S2 Network diagrams of comparisons on different outcomes. (A). Comparisons on the intensity of the EIM. (B) Comparisons on the duration of the EIM. EIM: etomidate-induced myoclonus; NS: normal saline

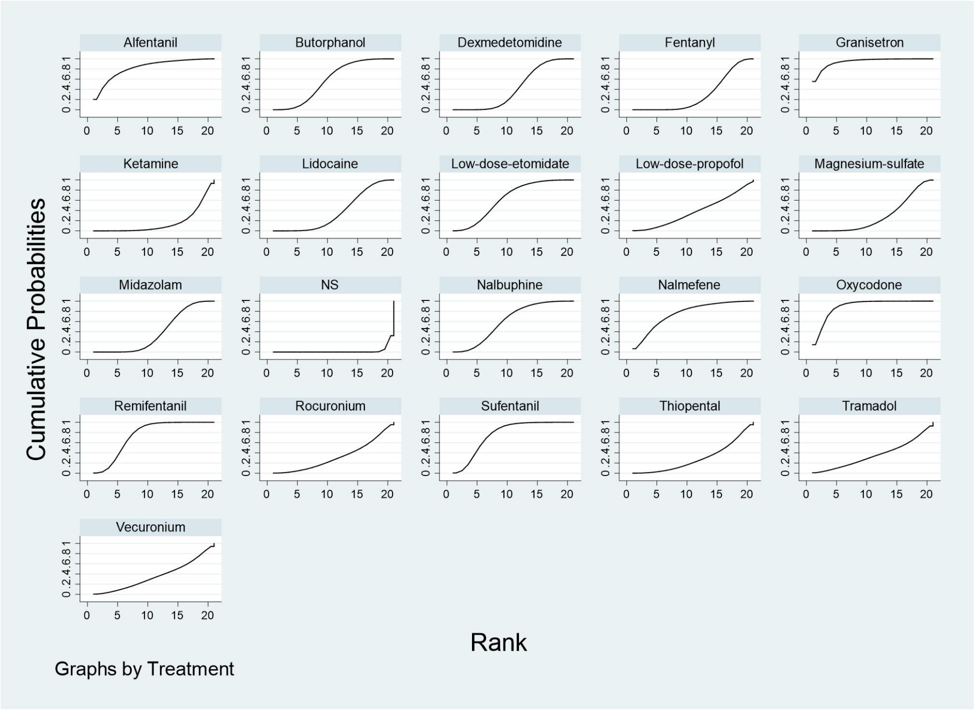

Figure S3A SUCRA and cumulative probability plots: The overall risk of EIM (prim ary outcome)

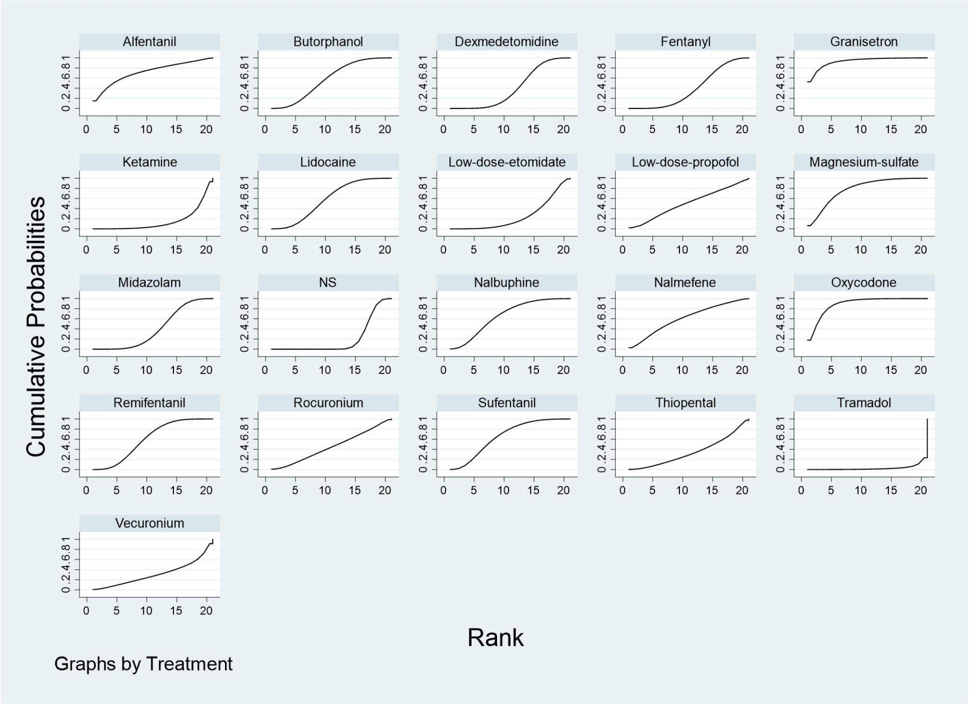

Figure S3B SUCRA and cumulative probability plots: The risk of EIM at a mild inte nsity level (secondary outcome)

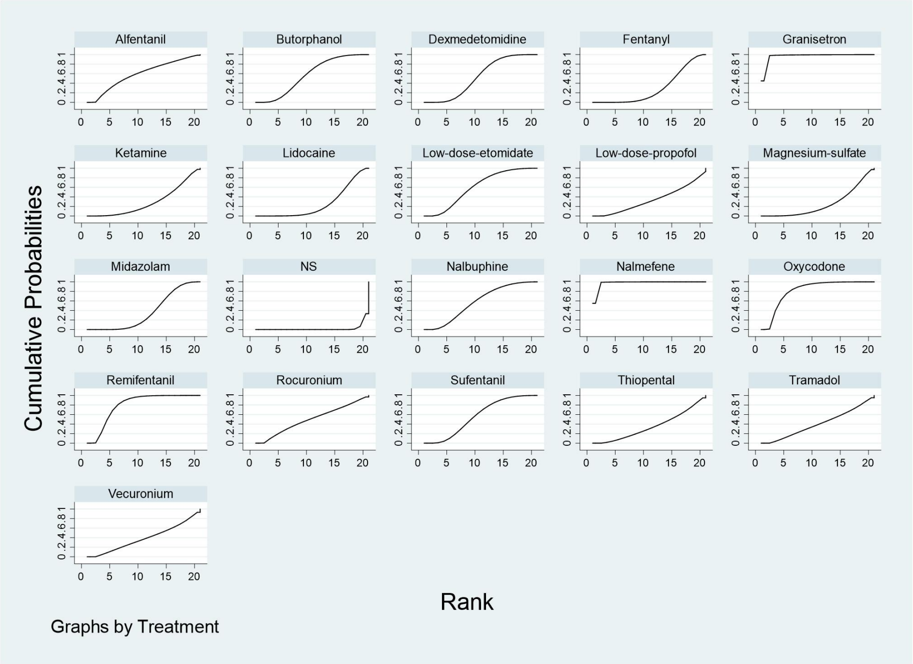

Figure S3C SUCRA and cumulative probability plots: The risk of EIM at moderate-to-severe intensity level (secondary outcome)

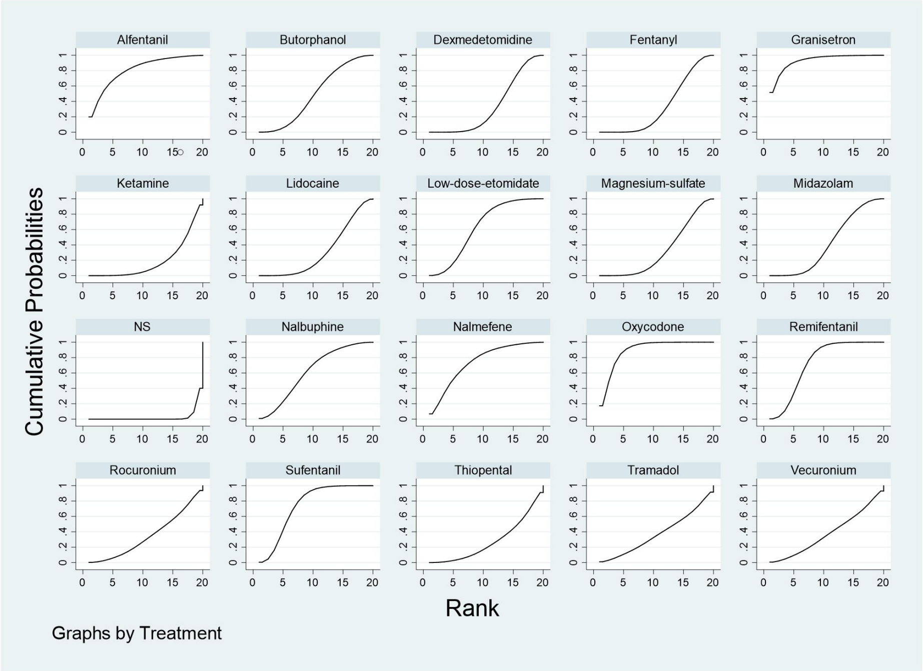

Figure S3D SUCRA cumulative probability plots: The overall risk of EIM (primary outcome) when trials at high risk of bias were excluded.

EIM: etomidate-induced myoclonus; NS: normal saline.

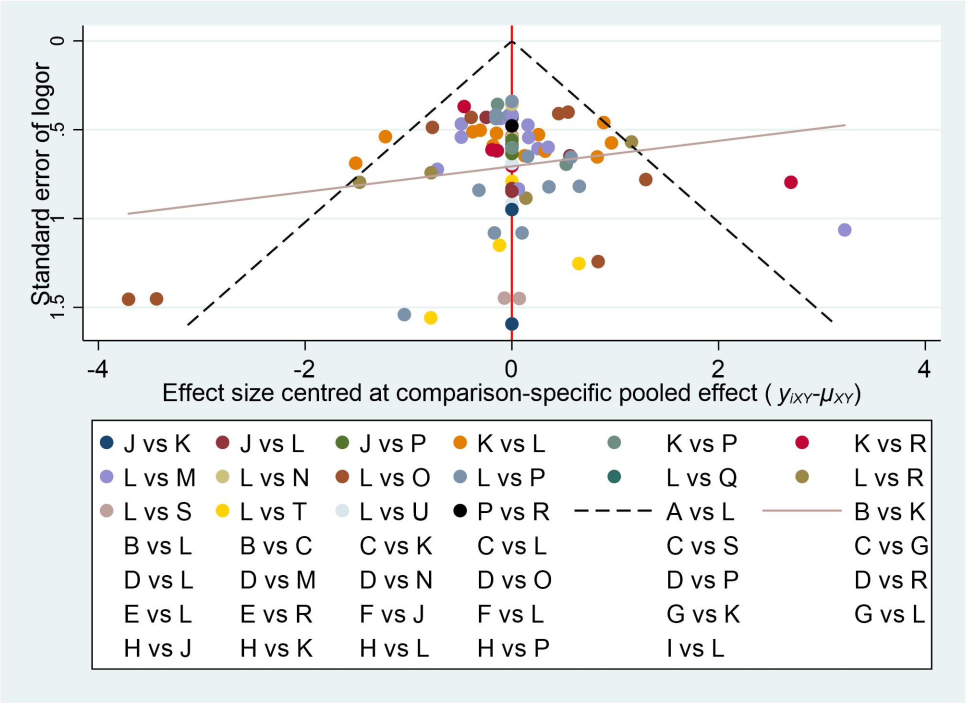

Figure S4A The overall risk of EIM (primary outcome): comparison-adjusted funne l plot

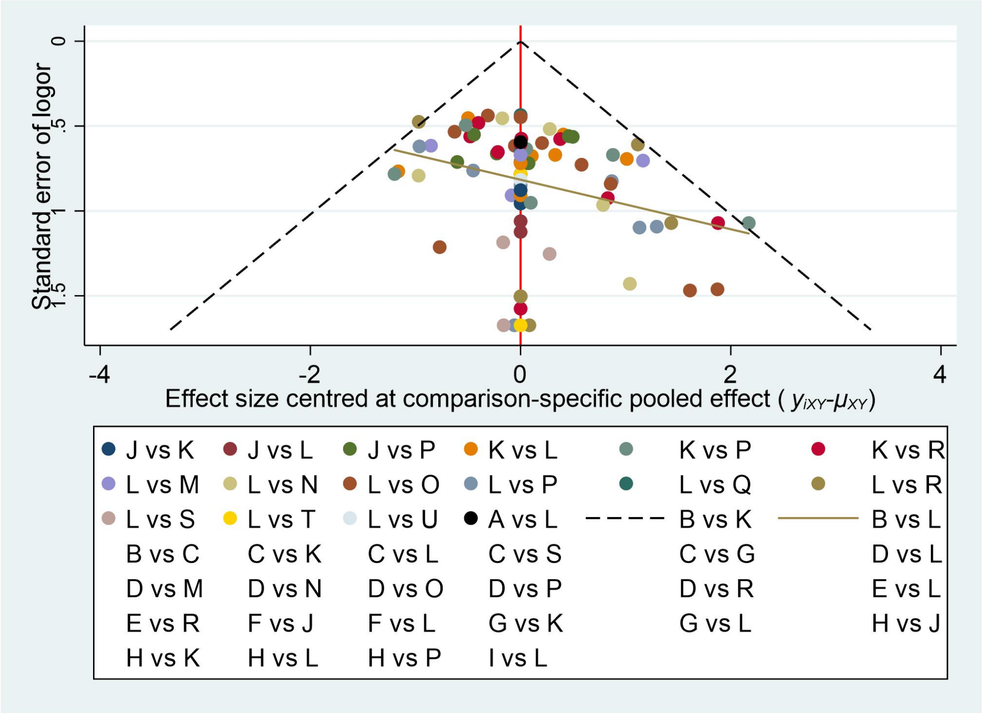

Figure S4B The risk of EIM at a mild intensity level (secondary outcome): comparis on-adjusted funnel plot

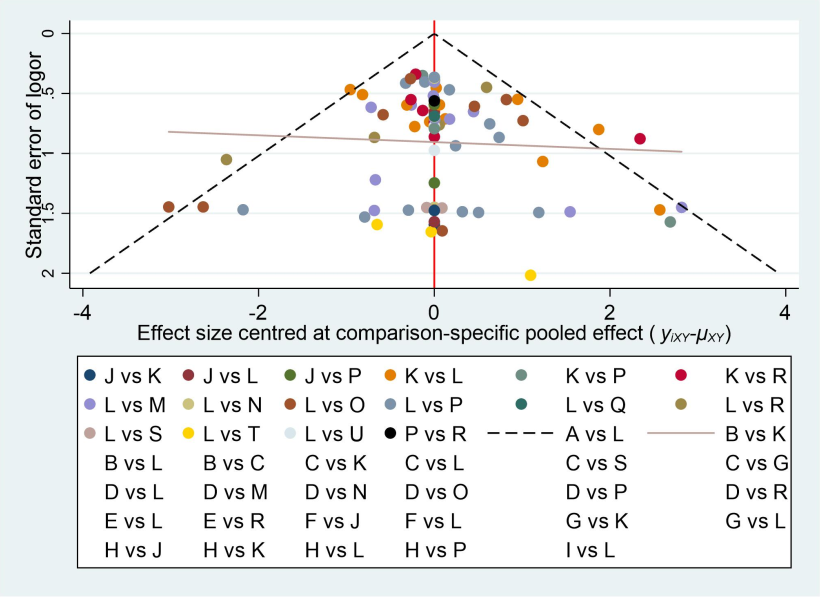

Figure S4C The risk of EIM at moderate-to-severe intensity level (secondary outcome): comparison-adjusted funnel plot

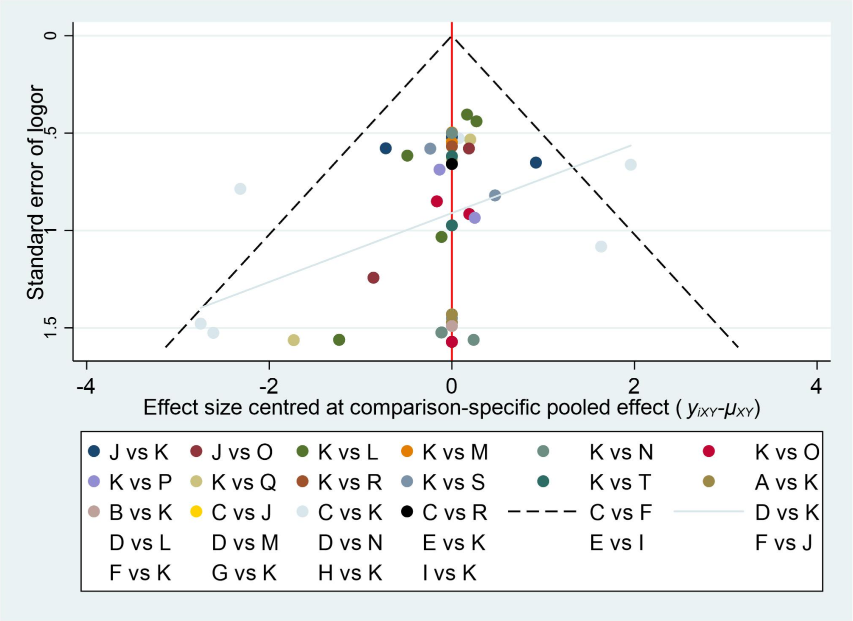

Figure S4D The risk of adverse events (secondary outcome): comparison-adjusted funnel plot

EIM: etomidate-induced myoclonus. A: Alfentanil; B: Butorphanol; C: Dexmedetomidine; D: Fentanyl; E: Granisetron; F: Ketamine; G: Lidocaine; H: Low-dose etomidate; I: Low-dose propofol; J: Magnesium sulfate; K: Midazolam; L: Normal saline; M: Nalbuphine; N: Nalmefene; O: Oxycodone; P: Remifentanil; Q: Rocuronium; R: Sufentanil; S: Thiopental; T: Tramadol; U: Vecuronium.

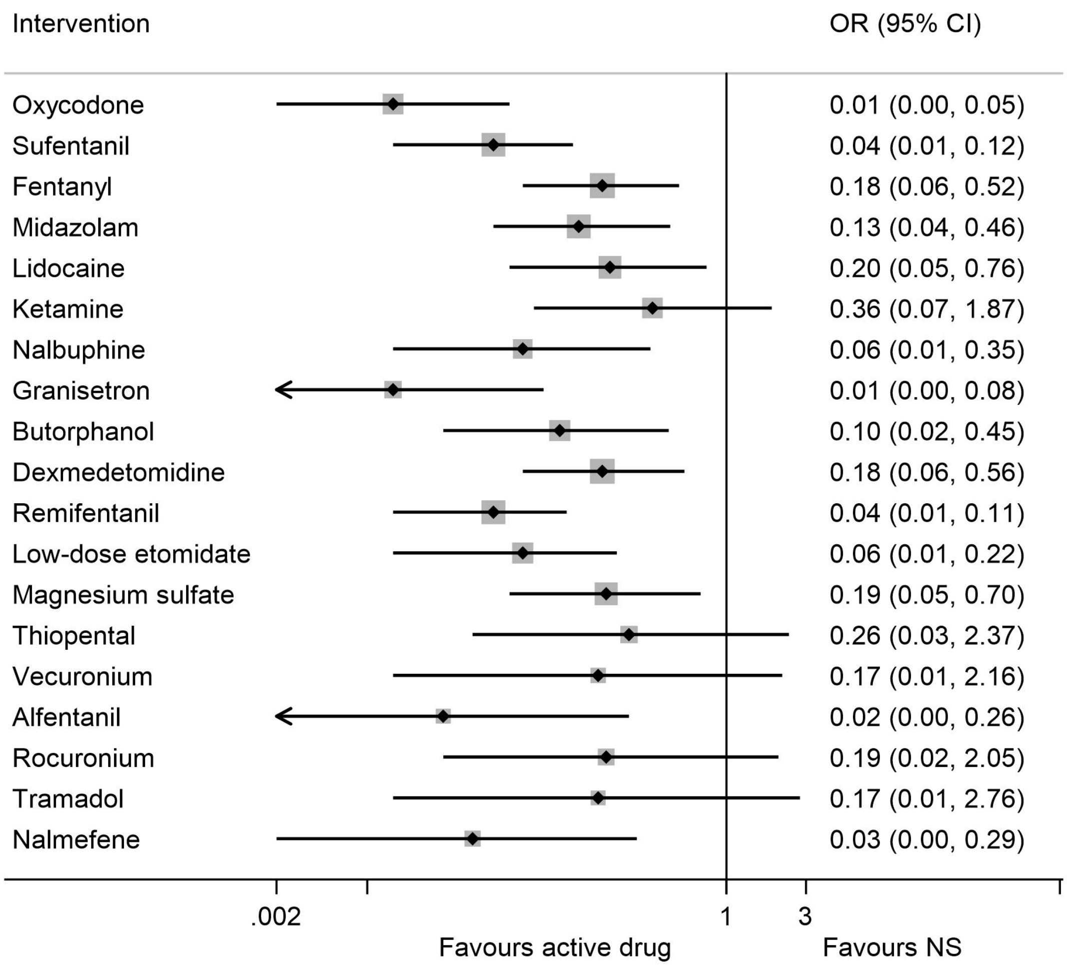

Figure S5 The forest of the overall risk of EIM when trials at high risk of bias were e xcluded. NS: Normal saline
